# MESSA: MEta-Server for protein Sequence Analysis

**DOI:** 10.1186/1741-7007-10-82

**Published:** 2012-10-02

**Authors:** Qian Cong, Nick V Grishin

**Affiliations:** 1 Departments of Biophysics and Biochemistry, University of Texas Southwestern Medical Center, 5223 Harry Hines Boulevard, Dallas, TX 75390-9050, USA; 2 Howard Hughes Medical Institute, University of Texas Southwestern Medical Center, 5223 Harry Hines Boulevard, Dallas, TX 75390-9050, USA

## Abstract

**Background:**

Computational sequence analysis, that is, prediction of local sequence properties, homologs, spatial structure and function from the sequence of a protein, offers an efficient way to obtain needed information about proteins under study. Since reliable prediction is usually based on the consensus of many computer programs, meta-severs have been developed to fit such needs. Most meta-servers focus on one aspect of sequence analysis, while others incorporate more information, such as PredictProtein for local sequence feature predictions, SMART for domain architecture and sequence motif annotation, and GeneSilico for secondary and spatial structure prediction. However, as predictions of local sequence properties, three-dimensional structure and function are usually intertwined, it is beneficial to address them together.

**Results:**

We developed a MEta-Server for protein Sequence Analysis (MESSA) to facilitate comprehensive protein sequence analysis and gather structural and functional predictions for a protein of interest. For an input sequence, the server exploits a number of select tools to predict local sequence properties, such as secondary structure, structurally disordered regions, coiled coils, signal peptides and transmembrane helices; detect homologous proteins and assign the query to a protein family; identify three-dimensional structure templates and generate structure models; and provide predictive statements about the protein's function, including functional annotations, Gene Ontology terms, enzyme classification and possible functionally associated proteins. We tested MESSA on the proteome of *Candidatus *Liberibacter asiaticus. Manual curation shows that three-dimensional structure models generated by MESSA covered around 75% of all the residues in this proteome and the function of 80% of all proteins could be predicted.

**Availability:**

MESSA is free for non-commercial use at http://prodata.swmed.edu/MESSA/

## Background

It is very beneficial to start a research project on a protein from computational analysis of its sequence. On the one hand, well-designed sequence analysis is an efficient way to obtain predictive information. Because a common evolutionary origin leaves distinct imprints on the sequences, structures and function of protein molecules, comparative computational methods supported by the accumulating biomolecular data often offer shortcuts to obtaining valuable hypotheses about a protein that cut the cost and time associated with experimental work. On the other hand, computational analysis of sequence can prevent potential misinterpretation of experimental data. The widely known argument about the report of a plant G-protein coupled receptor [1], which subsequently was suggested to be a cytoplasmic lanthionine synthetase-like protein by both computational analysis and experimental verification [2,3], illustrates the value of sequence analysis.

Many tools have been developed to serve the growing need for computational analysis of protein sequence. Such tools typically predict certain local sequence property, spatial structure or function of a query sequence. However, consensus-based meta-predictors usually produce better results than the individual tools they include [4,5]. In addition, when independent predictions are combined, errors in certain prediction can be revealed, leading to even better performance. For instance, in the recent ninth Critical Assessment of Structure Prediction (CASP9) experiment, even the top performing three dimensional structure predictors did not detect and remove the signal peptides in the target sequences [5], resulting in lower prediction quality as a hydrophobic signal peptide is likely to be incorrectly packed in the hydrophobic core. Therefore, to generate a reliable hypothesis on the basis of computational analysis, one needs to consult many predictors and integrate their results, making comprehensive analysis of a given protein sequence a non-trivial task.

Meta-severs have been developed to reduce such difficulty by combining various tools, integrating and displaying their results. Most meta-servers focus on one aspect of predictive analysis, for instance, Jpred on secondary structure [6], metaPrDOS on disordered regions [7], metaTM on transmembrane topology [8], Pcons [9] and 3D-Jury [10] on three-dimensional structure, and CombFunc [11]and ProKnow [12] on function. Other meta-servers incorporate more information to further accelerate sequence analysis, such as PredictProtein [13] for predictions of local sequence properties, SMART [14] for domain annotation and sequence motif prediction and GeneSilico [15] that focuses on secondary and spatial structure predictions.

However, as predictions of local sequence properties, spatial structure and function are usually interconnected, more accurate conclusions can be derived by addressing these questions together. Presence of transmembrane helixes or signal peptides, identification of conserved domains in the protein and predicted three-dimensional structure provide essential clues for function interpretation. At the same time, the predicted three-dimensional structure and function of a protein can validate transmembrane helix or signal peptide prediction to prevent false positives. Thus we developed a MEta-Server for protein Sequence Analysis (MESSA), which balances these predictions, integrates and outputs results about subcellular localization (secondary structure, disordered region, transmembrane, signal peptide, coiled coil and positional conservation prediction), function, three-dimensional structure and domain architecture. We tested MESSA on the proteome of a citrus pathogen, *Candidatus *Liberibacter asiaticus [16] and the results showed that MESSA provides structural and functional characterization for the majority of *Ca*. L. asiaticus proteins, which facilitates further understanding of these proteins and will aid in the experimental study of this bacterium.

## Results and discussion

### Interpretation of results from MESSA

MESSA utilizes a number of well-established programs, integrates their results and returns both a full web page with important information about and links to results of all the predictors and a summary page displaying consensus-based final predictions. The full version offers extensive information and is designed for careful manual analysis of a protein. The summary page is significantly simplified and provides predictions and their confidence that could be directly used by non-expert users.

### Description of the full output

The full output presents important information from all programs and provides links to the original results [17]. It contains the following seven sections:

#### Section I. Prediction of local sequence features

Local sequence property predictions, such as secondary structure and disordered region, are helpful for predicting three-dimensional structure, whereas signal peptide and transmembrane helix predictions are suggestive of the protein localization and function. This section summarizes the predictions of secondary structure, low-complexity regions, disordered regions, coiled coils, transmembrane helices and signal peptides. The programs used for each prediction and the explanation of their results are described in detail in Table 1. The result from each predictor is represented as one sting reporting each residue's predicted status. These strings are all aligned to the original protein sequence for the ease of comparison.

**Table 1:** Programs used in MESSA for prediction of local sequence features and their interpretation

Feature	Meaning	Programs used	Output
**Secondary structure**	Assist three-dimensional structure and domain boundary prediction.	PSIPRED (v2.0) [49] SSPRO (v4.0) [50] DISEMBL (v1.5) [51], coils	PSIPRED and SSPRO predict 3-states secondary structures (H: α-helix, E: β-strand, C: coils); DISEMBL predict coils (lower-case letters highlighted in pink)

**Disordered and flexible region**	Assist three-dimensional structure prediction.	DISEMBL (v1.5) [51], hot loops	Loops that are likely to have high B factors in the X-ray crystallography (lower-case letters highlighted in pink)
		
		DISEMBL (v1.5) [51], missing DISPRO (v1.0) [52] DISOPRED (v2.0) [53] IsUnstruct (v2.02) [54]	Residues without a defined structure (represented by star marks and highlighted in red)

**Transmembrane helix and Signal Peptide**	Predict subcellular localization and transmembrane, reveal topology of transmembrane proteins and provide hints to the protein function.	TMHMM (v2.0) [55] TOPPRED^a ^(v2.0) [56] HMMTOP^a ^(v2.0) [57] MEMSAT (v3.0) [58]	H: transmembrane helix (colored in blue); h: not confidently predicted transmembrane helix; o: periplasmic loop, i: cytoplasmic loop. x: loop region (not specified as periplasmic or cytoplasmic).
		
		MEMSATSVM [59] Phobius [60]	H: transmembrane helix (colored in blue); S: signal peptide (colored in green); h: unconfident transmembrane helix; o: periplasmic loop, i: cytoplasmic loop.
		
		SignalP (v3.0) [61] (HMM mode) SignalP (v3.0) [61] (NN mode)	S: signal peptide (highlighted in green) o: periplasmic region; x: do not have signal peptide

**Low-complexity region**	Reveal false positive hits of homology search caused by matching of low-complexity region.	SEG [62]	The part with low diversity in amino acid composition (highlighted in pink), likely to be disordered or fold as α helices, such as coiled coil

**Coiled coil**	Assist three-dimensional structure prediction.	COILS [63]	x: coiled coils, highlighted in yellow

**Conservation index**	Reveal essential residues for the folding and function of a protein.	BLAST (hits filtered by > 40% coverage and < 90% identity are included in the profile), AL2CO (calculate conservation indices based on profile) [64]	Sequence highlighted by the conservation (highlighted from white, through yellow to dark red as conservation increases)

^a^TOPPRED and HMMTOP are mainly designed to predict the topology of a given membrane protein rather than distinguish transmembrane proteins from cytoplasmic ones. Thus they may recognize the hydrophobic buried helices in cytoplasmic proteins as transmembrane helices, leading to a high false positive rate.

#### Section II. Close homologs for annotation transfer

Close homologs and orthologs usually preserve the function inherited from the common ancestor. MESSA shows the 10 closest confident homologs in the Swiss-Prot [18] and non-redundant (NR) databases detected by BLAST [19] (e-value cut-off: 0.001). The function annotations for homologs from the Swiss-Prot database are shown. As the Swiss-Prot annotations are of high quality [20], they offer a basis for function prediction by annotation transfer.

#### Section III. Prediction of gene ontology terms

Gene ontology (GO [21]) terms are the standard representation of protein attributes and they are widely used by researchers. MESSA predicts the GO terms associated with the query using the AMIGO server [22]. The 10 closest homologs in the GO databases detected by AMIGO and their associated GO terms are provided. Many of these GO terms could be directly transferred to the query.

#### Section IV. Prediction of enzyme commission number

Enzyme commission (EC) numbers describe the types of reactions enzymes catalyze and they are essential for understanding the function of proteins in the context of metabolic pathways. This section contains EC number predictions by three methods: transfer from close homologs in the Swiss-Prot database; and *de novo *prediction by the Ezypred server [23] and by the Enzyme Function Inference by a Combined Approach (EFICAz; version 2.5) software package [24,25]. For the first approach, the closely related Swiss-Prot entries and their assigned EC numbers are shown, while for the other two approaches, the predicted EC numbers and their definitions in the ENZYME nomenclature database [26] are listed.

#### Section V. Identification of functionally associated proteins

This section shows proteins that may function together with the query. The prediction mostly relies on the STRING database [27] that assigns functional associations between proteins by multiple criteria, such as physical interaction, expression pattern and genomic context. Moreover, when the query comes from a user-specified organism with complete genome sequence available, MESSA will provide a link to National Center for Biotechnology Information (NCBI) Gene database to show the genomic context of the query.

#### Section VI: Homologous protein families

Protein classification and the extensive information about each protein family in several databases [28-33] greatly assist in functional annotation. In this section, we provide ranked lists of top-scoring homologous protein families and conserved domains identified by RPS-BLAST [34] (e-value cut-off: 0.005) and HHpred server [35,36] (probability cut-off: 90%) in the NCBI Conserved Domain database. For each confidently detected domain, the relevant information and the alignment to the query are shown. This section allows users to explore rich information available for the related protein families, and is another useful resource for function prediction.

#### Section VII. Homologous structures and structure domains

Spatial structure prediction is an important aspect of sequence analysis. The predicted structure is indicative of protein function: the presence of conserved active sites and binding surfaces is useful in providing hypotheses about the function. As three-dimensional structure is usually more conserved among homologous proteins than function, a reliable structure prediction is achievable for most proteins [37], including many cases for which confident function predictions are not feasible. This section shows homologous structures in the Protein Data Bank (PDB) [38] and structure domains in the Structure Classification Of Protein (SCOP) database [39] detected by BLAST (e-value below 0.001), RPS-BLAST (e-value below 0.001) and HHpred server (probability higher than 80%). For each detected protein and protein domain, the alignment and the corresponding structure displayed by Jmol [40]) can be retrieved. The conservation of protein structures among homologs allows these structures, in most cases, to represent the general fold of the query protein and to be suitable templates for structure modeling. For structure domains detected in SCOP, we provide their classification hierarchy to highlight the evolutionary history and suggest similarities to other proteins.

### Description of the summary page

By integrating results from different methods, we generate the consensus-based final predictions for local sequence features, three-dimensional structure and function. We present these predictions as a summary page, which contains three sections:

#### Section I. Consensus-based prediction of local sequence properties (Figure 1A)

This section contains predictions of secondary structure, disordered regions, transmembrane helices, signal peptides, coiled coils and positional conservation indices. Except the last two, the predictions are based on the consensus between multiple predictors (described in **Methods**).

#### Section II. Function prediction (Figure 1B)

The predicted function annotation, GO terms and EC numbers (if the query is an enzyme) are shown in this section. Predictions are ranked by their confidence scores (details in **Methods**) assigned by MESSA. In addition, a confidence level ('very confident', 'confident' or 'probable') is provided for each prediction.

#### Section III. Spatial structural prediction (Figure 1C)

This section displays the three-dimensional structure models in Jmol for the query if a MODELLER key [41] is provided to enable homology modeling by MODELLER [42,43]. Otherwise, the templates selected by MESSA, their alignments to the query and confidence levels (details in **Methods**) will be listed.

### User-friendly interface

Users are required to input a query sequence (no less than 30 amino acids and no more than 4,000 amino acids) in FASTA or plain-text format and provide a non-commercial email address to initiate a MESSA job. Users are encouraged to select the organism name and organism type (such as eukaryote, Gram-negative and Gram-positive) from which the input sequence comes. This information is needed for signal peptide prediction, reciprocal BLAST and mapping the protein into its genomic locus. Once a job is submitted, MESSA will redirect the users first to a web page that summarizes the input information and later to a web page showing the status of the job. It generally takes about 30 minutes for a job to complete. For proteins from very large families, it may take several hours for the whole process to complete. While a job is in progress, MESSA can integrate and display available intermediate results upon user's request, allowing users to view results from fast programs in time. The users will be notified by email once the job is completed.

### Features of MESSA and comparison to other similar meta-servers

The most important feature of MESSA is a broad and balanced incorporation of predictions about local sequence features, domain architecture, three-dimensional structure and function. Another advanced feature is that MESSA integrates results from multiple predictors and generates consensus-based final predictions. These final predictions summarize the most important information and are very convenient for non-expert users. In addition, MESSA presents the results in a user-friendly way. For instance, the local sequence feature predictions are represented as single lines and aligned to the sequence. Detected structure templates can be directly and interactively visualized on the results page. Finally, MESSA relies on confident homology inferred by sequence and profile similarity for structure and function prediction. On the one hand, structure and function prediction without experimentally studied homologs, such as *de novo *folding, remains highly challenging, while the conservative homology-based approach ensures confident predictions in most cases. On the other hand, the rapid growth in the numbers of experimentally studied proteins and available protein three-dimensional structures has greatly increased the capability of homology-based structure-function annotation and ensures reasonable prediction coverage.

Widely used web servers similar to MESSA include PredictProtein, SMART and GeneSilico. These meta-servers utilize many programs and aim to facilitate highly integrated sequence analysis. PredictProtein offers rich information about the local sequence features of a protein, such as the secondary structure, transmembrane helices, protein sorting signals and functional sites. Unlike MESSA, PredictProtein does not offer detection of related protein families and pays less attention to three-dimensional structure prediction and function prediction. Moreover, it does not integrate results from different tools to provide a final prediction. Finally, due to the high volume of usage, PredictProtein only offers three free queries for academic users per year. SMART is specialized in annotating domain architecture. It offers predictions of signal peptides, transmembrane helices, low complexity regions and homologous structures detectable by BLAST. Compared with SMART, MESSA has a broader incorporation of programs and the ability to predict three-dimensional structure, predict function and to integrate results from multiple predictors. We consider GeneSilico to be the most similar to MESSA. Although GeneSilico is mainly a fold recognition meta-server for three-dimensional structure prediction, it offers information about related protein families and prediction of transmembrane helices as well. As opposed to GeneSilico's emphasis on three-dimensional structure prediction, MESSA aims to offer a well-balanced set of sequence-derived data to support comprehensive analysis of protein local sequence features, three-dimensional structures and function. As a result, MESSA limits tools for structural template identification to those few that are known to perform best. In addition, MESSA includes prediction of signal peptides, positional conservation, function annotation, GO terms and EC numbers, which are all helpful for function interpretation.

### Application of MESSA

**Figure 1:**
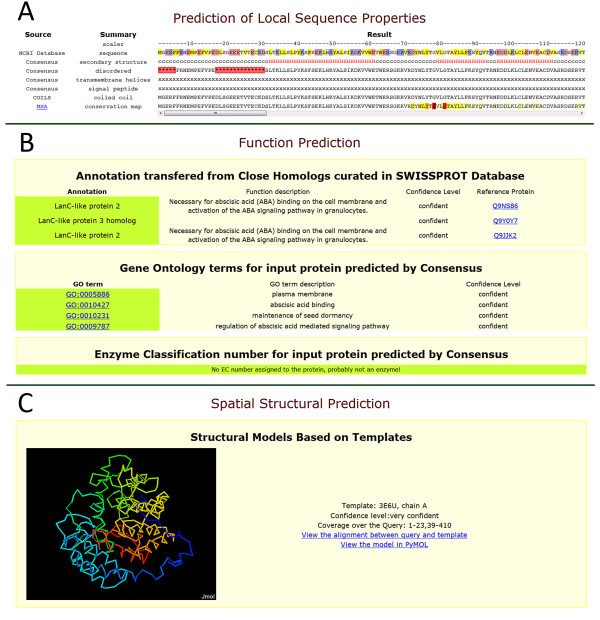
****Example of MESSA assisting with experimental data interpretation**. (A) **Local sequence predictions. **(B) **Function prediction. **(C) **Structure prediction.

The extensive information obtained by MESSA can help researchers to acquire knowledge and suggest hypotheses about a protein, and interpret experimental results. For instance, part of the result produced by MESSA for the purported G-protein coupled receptor by Liu *et al*. [1] (discussed in **Introduction**, refseq ID: NP_175700) is shown in Figure 1. The consensus-based prediction shows no transmembrane helices in this protein. The function prediction suggests that it is a homolog of lanthionine synthetase, which is not a transmembrane protein. Moreover, the predicted three-dimensional structure shows that the protein has 14 helices arranged as a toroid of two helical layers. Although the seven helices buried in the middle of the structure appear to be hydrophobic, the surface of the protein is largely hydrophilic. MESSA definitively suggests a potential error in the function proposed by Liu *et al*. [1], which was discovered later by both computational and experimental studies [2,3]. The evidence easily obtained from MESSA could assist with experimental data interpretation and help prevent false conclusions in such cases.

In addition, we tested MESSA on the proteome of *Ca*. L. asiaticus, a Gram-negative bacterium suggested to be the pathogen causing citrus greening disease. The results, together with information about this genome from other databases were assembled as a website [44]. In the genome sequence of *Ca*. L. asiaticus, the gene prediction pipeline from NCBI and the SEED detected 1,233 protein coding genes, with 1,046 in common. In addition, 58 protein coding genes that are identified by a single gene prediction pipeline display confident homology to other proteins in the NR database. We consider these 1,104 hypothetical protein coding genes to be confidently predicted. The remaining 128 inconsistently predicted genes encode products that are of a relatively small size (usually less than 60 residues), include low complexity sequences, and lack similarity to any known protein. A large portion of them may represent falsely predicted open reading frames and were not considered in the analysis.

**Figure 2:**
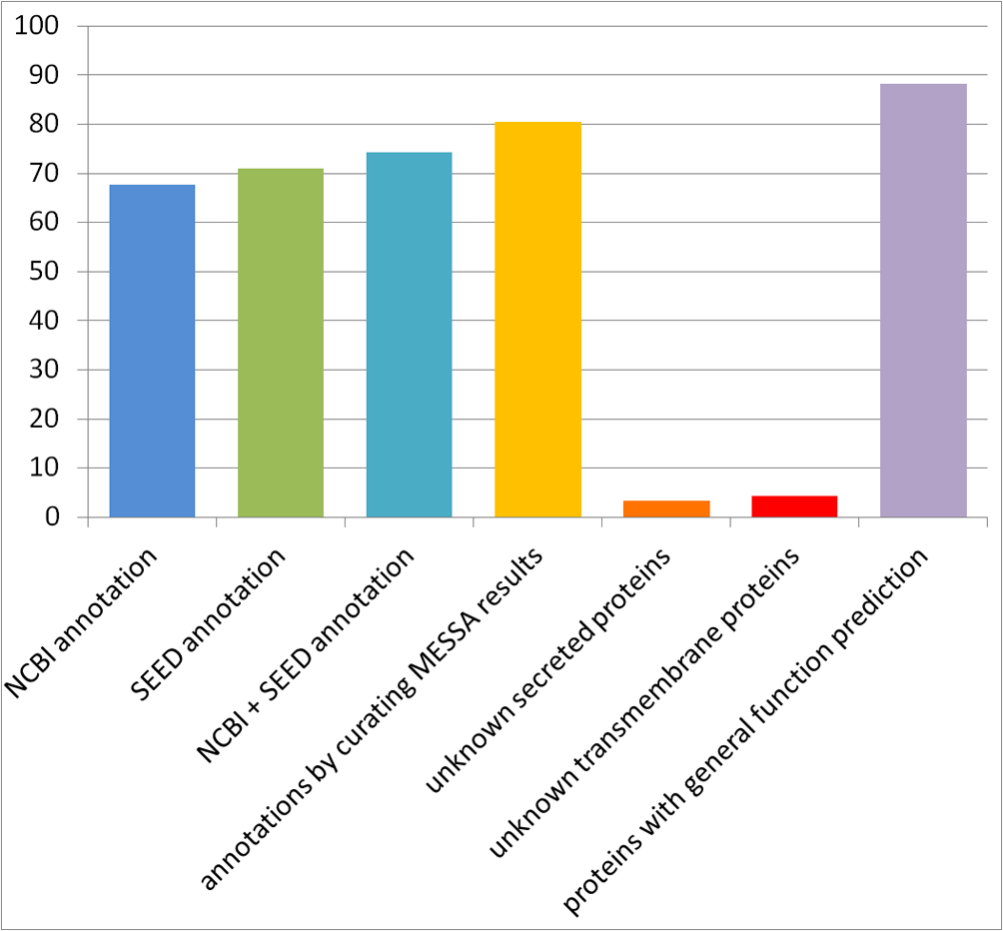
**Fractions of proteins in *Ca*. L. asiaticus that are annotated by different methods**.

Based on the MESSA output, we manually analyzed all 1,104 proteins encoded by the confidently predicted genes to predict their subcellular localization, three-dimensional structure and function. As shown in Figure 2, confidently identified homology to known proteins or protein families allows us to predict the function for 80.2% of these proteins, while NCBI and SEED annotated 67.7% and 71.0% of them, respectively. Moreover, the additional information collected by MESSA allows us to revise 32 annotations by the SEED and 44 by NCBI to different or more specific function predictions. Out of the 219 proteins without function predictions, 39 are predicted to have a signal peptide and thus likely function in either periplasmic or extracellular space while 49 are likely to be transmembrane proteins. These proteins take up 40.2% of the unknown proteins and their subcellular localization indicates their general function in communicating with the environment. As this bacterium is a plant pathogen, these periplasmic or extracellular proteins might be virulence factors whose homologs become hard to detect due to accelerated evolution. (All function annotations are listed in Table S1 in Additional file 1).

Moreover, MESSA detects homologous structures for template-based structure modeling of *Ca*. L. asiaticus proteins. The confident structure templates identified by MESSA (HHsearch probability above 90%, PSI-BLAST or RPS-BLAST e-value below 0.005) and verified manually cover 74.3% of all residues in the *Ca*. L. asiaticus proteome. In addition, some of the sequence regions without confidently identified structure templates are predicted to be disordered by no less than two predictors and tend to appear at the boundaries of protein domains. These regions count for another 5.8% of all residues. At a protein level, 65.9% of all *Ca*. L. asiaticus proteins exhibit greater than 80% coverage by the confident structure templates and predicted disordered regions. It is important to note that we adopted conservative criteria for selecting structure templates, which may underestimate the number of proteins in a bacterial genome that can be confidently predicted by homology modeling. In summary, our results indicate that MESSA can help biologists to efficiently gain understanding of proteins and will be useful to suggest hypotheses for experimental pursuit.

### Integration of several approaches enhances the quality of sequence analysis

To illustrate how comprehensive information can be integrated for more confident predictions, we carried out a pilot study to identify proteins that can be secreted to the periplasm through the Sec protein secretion pathway in *Ca*. L. asiaticus. These proteins are of particular interest, as some of them could be virulence factors of this pathogenic bacterium. Proteins secreted by the Sec machinery are characterized by a signal peptide at their N-termini, which could be predicted by the well-established algorithms included in MESSA. Out of the 1,104 proteins in *Ca*. L. asiaticus, 217 are predicted to have signal peptides by at least one algorithm. However, signal peptide prediction by itself is not enough to suggest the subcellular localization due to false predictions and the fact that some transmembrane proteins also possess signal peptides [45].

We manually examined all these 217 candidates with predicted signal peptides. In addition, we briefly curated all other proteins that are predicted to have transmembrane helices to identify possible false negatives, as some signal peptides might be falsely predicted as transmembrane helices, especially when the translation initiation sites are mispredicted. Predictions and supporting evidence for each protein are listed in Table S2 in Additional file 2. As a result, we hypothesize that 84 proteins in this bacterium are secreted to periplasm though the Sec machinery. The consensus between different predictors is the main indicator of prediction confidence, and most of these 84 verified proteins and their orthologs have signal peptides that can be consistently identified by at least two methods out of four. In addition to simple consensus, other evidence provided by MESSA was essential to ensure reliable predictions.

In one case, the hypothetical ribosomal protein L35 (locus: CLIBASIA_01020; gi: 254780319) [46] is predicted to have a signal peptide by three out of four predictors. However, all the closely related proteins and protein families identified by MESSA support its function of being associated with the ribosome, as opposed to being secreted. Additionally, the gene encoding this protein is located within an operon containing other predicted ribosome proteins coding genes. In the three-dimensional structure of the ribosome complex (PDB id: 3BBO) [47], the N-terminus of ribosomal protein L35 is buried in the complex, which more likely accounts for the hydrophobic segment that is falsely predicted as a signal peptide.

Many proteins from the initial list of 217 candidates were excluded due to the following reasons: the signal peptide cannot be consistently predicted (predicted by only one out of four methods); the protein has multiple transmembrane helices, such as the sensory box/GGDEF family protein (locus: CLIBASIA_01765; gi: 254780468); the confidently predicted function of the protein suggests that the protein is located in the inner membrane or cytoplasm; close homologs lack signal peptides. It is important to note that multispan transmembrane helical proteins with N-terminal signal peptides do exist, although not common in bacteria [48]. However, they will be localized in the membrane by other transmembrane helices regardless of whether the signal peptides will be cleaved or not.

In summary, the signal peptide predictors provided the initial candidates of secreted proteins. Starting from these 217 candidates, integration of additional information collected by MESSA, such as the consensus between different predictors, other sequence features (transmembrane helices), features of the close homologs, the predicted function and spatial structures, allows us to propose a more confident list of 84 proteins that are likely secreted by the Sec pathway. Comprehensive information collected by MESSA allows us to correct the mistakes by computer programs and generate more reliable hypothesis about a protein. Due to the limited information available for some proteins and the limitation that we only curated proteins with predicted signal peptides or transmembrane helices, it is possible that incorrect predictions still exist even after careful manual curation.

## Conclusions

We developed MESSA, a web server that integrates the results of a dozen state-of-the-art sequence analysis tools to provide predictions on local sequence properties, three-dimensional structure and function of a given protein. MESSA offers a user-friendly interface and display the results in a manner convenient for navigation. Our benchmark study showed that MESSA was able to offer extensive information for most of the proteins in a genome. We hope MESSA can help biologists to gain insights about proteins under study.

## Methods

### Assemble computational sequence analysis tools and integrate their results

For a given protein sequence, MESSA carries out the following analyses:

First, MESSA uses multiple predictors (listed in Table 1) [49-64] with default parameters to predict secondary structure, disordered regions, low-complexity regions, transmembrane helices, signal peptides, coiled coils and positional conservation indices. The results from multiple tools for each local feature are then combined to get a final prediction. At each sequence position, the final prediction is based on votes from individual methods. Most methods have a single vote, while PSIPRED for secondary structure prediction and Phobius for signal peptide prediction are counted as two votes due to their documented high accuracy [60,65].

Second, homologs (e-value cut-off: 0.001) from NR and Swiss-Prot databases are identified. For each confident hit from the NR database, its taxonomy information is obtained through NCBI Entrez Programming Utilities (E-utilities). For hits from Swiss-Prot, the homology relationships to the query are further evaluated by the statistic from BLAST and whether the query and a certain hit are the reciprocal best BLAST hits of each other [66,67]. The details of these criteria are shown in Table 2, and each hit will be assigned a confidence score (ranging from 0 to 12 points) to evaluate its similarity to the query. A confidence level is assigned to a hit based on this confidence score, with no less than 10 points, 8 points and 6 points to be 'very confident', 'confident' and 'probable', respectively. In addition, the EC numbers annotated for hits from the Swiss-Prot database are obtained from Swiss-Prot and the ENZYME nomenclature databases. These EC numbers and their confidence levels are used to predict the EC number of the query.

**Table 2:** Confidence score of homologs from Swiss-Prot database.

Evaluation method	Criteria	Points
**BLAST e-value**	< 0.001	1

**Sequence identity between the query and the hit**	identity 30% to 50%, coverage > 40%	1
	identity 50% to 70%, coverage > 40%	2
	
	identity 70% to 90%, coverage > 40%	3
	
	identity 90% to 99%, coverage > 40%	4
	
	identity > 99%, coverage > 40%	5

**BLAST alignment coverage for both query and hit**	60% to 80%	1
	
	80% to 100%	2

**The query against the proteome associated with the hit**	Best hit	2
	
	N/A	1

**The hit against the proteome associated with the query**	Best hit	2
	
	N/A	1

Third, evolutionarily related proteins with assigned GO terms are identified by querying the AMIGO server. The GO terms associated with these hits are candidates to transfer to the query. Their relevance to the query is evaluated by the similarity between the hits and the query, consensus in GO terms annotated for different hits and the evidence used to assign these GO terms to the hits in the GO database (details in Table 3). Each GO term will receive a confidence score ranging from 0 to 12 points to evaluate its relevance to the query. In addition, as GO terms are hierarchical, once a GO term is assigned to a protein, its parents should be automatically assigned. We uses the SUPERFAMILY database [68] to obtain the parent terms of each GO term, and a parent GO term will get the highest confidence score of its offspring. Similar to the criteria used before, GO terms with confidence scores no less than 10 points, 8 points and 6 points are considered to be 'very confident', 'confident' and 'probable', respectively.

**Table 3:** Confidence score of predicted gene ontology terms

Evaluation method	Criteria	Points
**BLAST e-value**	0.001	1

**Sequence identity between the query and the hit**	identity 30% to 50%, coverage > 40%	1
	
	identity 50% to 70%, coverage > 40%	2
	
	identity 70% to 90%, coverage > 40%	3
	
	identity 90% to 100%, coverage > 40%	4

**Alignment coverage for query and hit**	60% to 80%	1
	
	80% to 100%	2

**Evidence code of the GO term assigned to the hit**	EXP, IDA	3
	
	IPI, IMP, IGI, IEP, ISO, TAS	2
	
	ISS, ISA, ISM, IGC, IBA, IBD, IKR, IRD, RCA, NAS, IC, IEA	1

**Consensus bonus**	Associated with no less than three hits	2

EXP: inferred from experiment; GO: Gene Ontology; IBA: inferred from biological aspect of ancestor; IBD: inferred from biological aspect of descendant; IC: inferred by curator; IDA: inferred from direct assay; IEA: inferred from electronic annotation; IEP: inferred from expression pattern; IGC: inferred from genomic context; IGI: inferred from genetic interaction; IKR: inferred from key residues; IMP: inferred from mutant phenotype; IPI: inferred from physical interaction; IRD: inferred from rapid divergence; ISA: inferred from sequence alignment; ISM: inferred from sequence model; ISO: inferred from sequence orthology; ISS: inferred from sequence or structural similarity; NAS: non-traceable author statement; RCA: inferred from reviewed computational analysis; TAS: traceable author statement.

Fourth, MESSA assembles three tools to predict whether a query is an enzyme and the EC number of a query that is predicted to be an enzyme. In addition to transferring EC numbers from closely related Swiss-Prot entries, MESSA utilizes the EFICAz (version 2.5) package and the Ezypred server to distinguish enzymes from non-enzymes and to directly predict the EC numbers. EFICAz predict all four layers of EC number (such as 1.1.1.1) while Ezypred predicts only the first two layers (such as 1.1.-.-). The predictions from these three resources are combined to generate a confidence score for each predicted EC number, as detailed in Table 4. Predictions with confidence score no less than 7, 5 and 3 are considered to be 'very confident', 'confident' and 'probable', respectively.

**Table 4:** Confidence score of predicted Enzyme Commission numbers

Evaluation method	Criteria	Points
**Confidence score of homologous Swiss-Prot hit for EC number transfer**	≥ 6 and < 8	1
	
	≥ 8 and < 10	2
	
	≥ 10	3

**Consensus bonus**	If the EC number is assigned for at least three different Swiss-Prot hits	1

**Ezypred prediction (no confidence assigned to prediction)**	If the EC number agrees with the prediction of Ezypred	2

**EFICAz prediction confidence**	Low confidence prediction	2
	
	0.6 to 0.7	2.5
	
	0.7 to 0.8	3
	
	0.8 to 0.9	3.5
	
	0.9 to 1	4

Fifth, when the query sequence is from a user-specified organism with available complete genome sequence, MESSA will map the query to its genomic locus by BLAST and show the genomic context of the query through NCBI E-utilities. Moreover, MESSA sends the query sequence to the STRING server to predict the functionally associated proteins.

Sixth, homologous protein families are detected from the Conserved Domain database by RPS-BLAST (e-value cutoff: 0.005) and HHpred server (probability cutoff: 90%). These protein families and protein domains are mapped to the query sequence.

Seventh, to detect evolutionarily related proteins with available three-dimensional structures and reveal domain architectures, we use three protocols: first, BLAST against PDB (e-value cut-off: 0.001); second, RPS-BLAST (e-value cut-off: 0.01); and third, HHpred server (probability cut-off: 80%) against the 70% sequence identity representatives of all PDB and SCOP (version 1.75) entries. These homologs are used to select templates for homology modeling. All templates are ranked by a confidence score described in Table 5. From the top of this ranking list, we select non-redundant templates, requiring each new template to cover at least 30 additional residues that are not covered by selected higher-ranked templates. Since the MODELLER software license requires the users to have a MODELLER key, homology modeling based on these templates will be performed only if such a key is provided by a user.

**Table 5:** Evaluation of homology modeling templates

Evaluation method	Criteria	Points
**Sequence identity for BLAST, RPS-BLAST and HHSearch**	20% to 40%	1
	
	40% to 60%	2
	
	60% to 80%	3
	
	80% to 90%	4
	
	90% to100%	5

**HHsearch probability**	80% to 85%	1
	
	85% to 90%	2
	
	90% to 99%	3
	
	99% to 99.99%	4
	
	99.99% to 100%	5

**BLAST and RPS-BLAST e-value**	1e-6 to 1e-2	1
	
	1e-6 to 1e-18	2
	
	1e-18 to 1e-54	3
	
	< 1e-54	4

**Consensus bonus**	Predicted by two methods	1
	
	Predicted by three methods	2

Finally, the results from all these procedures are parsed and presented as two web pages: the first one presents all the original results; the second one contains the final consensus-based predictions by integrating the results from different predictors.

### Application of MESSA to the proteome of *Ca*. L. asiaticus and manual curation

All the sequences of *Ca*. L. asiaticus proteins predicted by NCBI gene prediction pipeline [69] were downloaded from the GenBank database [70] and additional proteins that were detected by the SEED (Genome annotation web service on the basis subsystems) [71,72] but missed by NCBI were added. The relevant information about these proteins was obtained from NCBI [73], the SEED and Kyoto Encyclopedia of Genes and Genomes (KEGG) [74,75]. Computational analysis by MESSA was performed on each protein and the results were constructed as a website [45].

Based on the information from this website, we manually curated the functional assignment, predicted the subcellular localization and selected structure templates for each protein. Functional annotations were mainly based on their close relationship to certain protein families or a certain reviewed entry in the Swiss-Prot database. This relationship was verified on the one hand by the statistical significance, coverage and alignment quality between the *Ca*. L. asiaticus protein and the identified proteins or families, and on the other hand by the consensus between different methods. In cases where agreement between methods was lacking or statistical support was marginal, identification of conserved sequence motifs, inspection of predicted structure and clustering of homologous proteins were applied to obtain function predictions.

## Availability and requirements

**• Project name: **MEta-Server for protein Sequence Analysis (MESSA)

**• Project home page: **http://prodata.swmed.edu/MESSA/

**• Operating system(s): **This is a web server and users should access it through web browsers.

**• Programming language: **Python, HTML and Javascript

**• Other requirements: **The server is tested on Mozilla Firefox (version >= 12.0), Microsoft Internet Explorer (version >= 8.0) Google Chrome and Safari (Version >= 5.0). Correct display of the result page requires Java (TM) Platform to be installed and enabled by the browsers.

**• License: **Academic Free License

**• Any restrictions to use by non-academics: **The users need to provide an academic email address to initiate a job.

## Authors' contributions

QC designed and wrote the software and the web interface, performed the analysis on the *Ca*. L. asiaticus proteome, and drafted the manuscript. NVG designed the conceptual layout. NVG and QC selected the methods to be used and edited the manuscript. Both authors approved the final manuscript.

## Supplementary Material

Additional file 1 **Function annotations of the *Ca*. L. asiaticus proteins**. This file contains the annotations of the *Ca*. L. asiaticus proteins from NCBI, SEED and the information provided by us on the basis of MESSA results and manual curation. When MESSA offers updated or modified annotations, the updated annotations are highlighted in green and the original SEED or NCBI annotations are highlighted in yellow. Click here for file

Additional file 2 **Curation of proteins predicted to be secreted by the Sec pathway**. This file contains proteins that are predicted to have signal peptides by computer programs. For each protein, the evidence to support or refute the prediction and the final judgment after manual curation is listed. Click here for file
