# Supplementary material for: MESSA: MEta-Server for protein Sequence Analysis
Source: BMC Biol. 2012 Oct 2;10:82. doi: 10.1186/1741-7007-10-82 (PMC3519821; doi:10.1186/1741-7007-10-82)
Supplement: Additional file 1 — Function annotations of the Ca. L. asiaticus proteins. This file contains the annotations of the Ca. L. asiaticus proteins from NCBI, SEED and the information provided by us on the basis of MESSA results and manual curation. When MESSA offers updated or modified annotations, the updated annotations are highlighted in green and the original SEED or NCBI annotations are highlighted in yellow. [file 1741-7007-10-82-S1.PDF]

NCBI_gi	SEED_id	NCBI_annotaion	SEED_annotation	Curated_funciton_prediction	Comments
**254780122**	537021.9.peg.1	hypothetical protein	hypothetical protein	unknown	
**254780123**	537021.9.peg.2	hypothetical protein	hypothetical protein	unknown	
**254780124**	537021.9.peg.3	hypothetical protein	Phage protein	endo/exonuclease	
**254780125**	537021.9.peg.4	prophage antirepressor	Phage antirepressor protein	prophage antirepressor	
**254780126**	537021.9.peg.5	hypothetical protein	unknown	phage protein, may related to DNA binding	
**254780127**	537021.9.peg.6	putative DNA polymerase from bacteriophage origin	DNA polymerase I (EC 2.7.7.7)	prophage DNA polymerase I (EC 2.7.7.7)	
**254780128**	537021.9.peg.7	VRR-NUC domain-containing protein	hypothetical protein	restriction endonuclease	
**254780129**	537021.9.peg.8	hypothetical protein	DNA helicase, phage-associated	prohage DNA/RNA helicase	1/2 of a protein
**254780130**	537021.9.peg.9	hypothetical protein	DNA helicase, phage-associated	prohage DNA/RNA helicase	1/2 of a protein
**254780131**	537021.9.peg.10	DNA ligase, NAD-dependent	DNA ligase, NAD-dependent	NAD-dependant DNA ligase	
**254780132**	537021.9.peg.11	guanylate kinase	Guanylate kinase (EC 2.7.4.8)	guanylate kinase (EC 2.7.4.8)	
**254780133**	N/A	hypothetical protein	N/A	unknown	
**254780134**	537021.9.peg.12	phage-related integrase/recombinase	integrase	phage integrase	
**254780135**	537021.9.peg.13	hypothetical protein	hypothetical protein	unknown	has signal peptide
**254780136**	537021.9.peg.14	ribonucleotide-diphosphate reductase subunit beta	Ribonucleotide reductase of class Ia (aerobic), beta subunit (EC 1.17.4.1)	ribonucleotide-diphosphate reductase subunit beta (EC 1.17.4.1)	this is just part of the protein,
**254780137**	537021.9.peg.16	malic enzyme	NADP-dependent malic enzyme (EC 1.1.1.40)	malic enzyme (EC 1.1.1.40)	
**254780138**	537021.9.peg.17	ABC transporter	ABC-type transport system involved in resistance to organic solvents, permease component USSDB6A	ABC-type import for membrane lipid (from outer membrane to inner membrane), transmembrane component	transmembrane protein
**254780139**	537021.9.peg.18	putative ATP-binding component of ABC transporter	probable ABC transporter, ATP-binding protein	ABC-type import for membrane lipid (from outer membrane to inner membrane), ATPase component	
**254780140**	537021.9.peg.19	putative ABC transporter, substrate-binding protein	putative solute-binding component of ABC transporter	ABC-type import for membrane lipid (from outer membrane to inner membrane), auxiliary component	transmembrane protein
**254780141**	537021.9.peg.20	putative ABC transporter protein	hypothetical protein	ABC-type import for membrane lipid (from outer membrane to inner membrane), auxiliary component	has signal peptide
**254780142**	537021.9.peg.22	DNA-directed RNA polymerase subunit beta'	DNA-directed RNA polymerase beta’ subunit (EC 2.7.7.6)	DNA-directed RNA polymerase subunit beta' (EC 2.7.7.6)	
**254780143**	537021.9.peg.23	DNA-directed RNA polymerase subunit beta	DNA-directed RNA polymerase beta subunit (EC 2.7.7.6)	DNA-directed RNA polymerase subunit beta (EC 2.7.7.6)	
**254780144**	537021.9.peg.24	50S ribosomal protein L12P	LSU ribosomal protein L7/L12 (P1/P2)	50S ribosomal protein L12P	
**254780145**	537021.9.peg.25	50S ribosomal protein L10	LSU ribosomal protein L10p (P0)	50S ribosomal protein L10	
**254780146**	537021.9.peg.27	50S ribosomal protein L1	LSU ribosomal protein L1p (L10Ae)	50S ribosomal protein L1	
**254780147**	537021.9.peg.28	50S ribosomal protein L11	LSU ribosomal protein L11p (L12e)	50S ribosomal protein L11	
**254780148**	537021.9.peg.29	transcription antitermination protein NusG	Transcription antitermination protein NusG	transcription antitermination protein NusG	
**254780149**	537021.9.peg.30	hypothetical protein	preprotein translocase	Preprotein translocase subunit SecE	transmembrane protein
**254780150**	537021.9.peg.31	translation elongation factor Tu	Translation elongation factor Tu	translation elongation factor Tu	
**254780151**	537021.9.peg.32	tRNA-specific 2-thiouridylase MnmA	tRNA (5-methylaminomethyl-2-thiouridylate)-methyltransferase (EC 2.1.1.61)	tRNA methyl transferase (EC 2.1.1.61)	wrong start protein should start from the second M
**N/A**	537021.9.peg.33	N/A	D-3-phosphoglycerate dehydrogenase	D-3-phosphoglycerate dehydrogenase	1/4 of a protein
**N/A**	537021.9.peg.34	N/A	D-3-phosphoglycerate dehydrogenase (EC 1.1.1.95)	D-3-phosphoglycerate dehydrogenase	1/4 of a protein
**254780152**	537021.9.peg.35	D-3-phosphoglycerate dehydrogenase	D-3-phosphoglycerate dehydrogenase (EC 1.1.1.95)	D-3-phosphoglycerate dehydrogenase	1/4 of a protein
**N/A**	537021.9.peg.36	N/A	hypothetical protein	D-3-phosphoglycerate dehydrogenase	1/4 of a protein
**254780153**	537021.9.peg.37	phosphoserine aminotransferase	Phosphoserine aminotransferase (EC 2.6.1.52)	phosphoserine aminotransferase (EC 2.6.1.52)	
**254780154**	537021.9.peg.38	hypothetical protein	hypothetical protein	unknown	
**254780156**	537021.9.peg.39	putative inositol-1-monophosphatase	Inositol-1-monophosphatase (EC 3.1.3.25)	inositol-1-monophosphatase (EC 3.1.3.25)	
**254780157**	537021.9.peg.40	phosphoribosylaminoimidazole carboxylase catalytic subunit protein	Phosphoribosylaminoimidazole carboxylase catalytic subunit (EC 4.1.1.21)	phosphoribosylaminoimidazole carboxylase (EC 4.1.1.21)	
**254780158**	537021.9.peg.41	phosphoribosylaminoimidazole carboxylase ATPase subunit	Phosphoribosylaminoimidazole carboxylase ATPase subunit (EC 4.1.1.21)	phosphoribosylaminoimidazole carboxylase ATPase (EC 4.1.1.21)	
**N/A**	537021.9.peg.42	N/A	hypothetical protein	missing in NCBI database, but should have it, it is ribosome protein	missing in NCBI database, but should

					have it
**254780159**	537021.9.peg.43	hypothetical protein	hypothetical protein	unknown	transmembrane protein
**254780160**	537021.9.peg.44	hypothetical protein	hypothetical protein	unknown	no reliable prediction
**254780161**	537021.9.peg.45	phytoene synthase protein	Phytoene synthase (EC 2.5.1.32)	phytoene synthase protein (EC 2.5.1.32)	
**254780162**	537021.9.peg.46	hypothetical protein	hypothetical protein	unknown	wrong gene prediction?
**254780163**	537021.9.peg.47	ATP-dependent Clp protease ATP-binding subunit	ATP-dependent Clp protease ATP-binding subunit ClpA	ATP-dependent Clp protease ATP-binding subunit	
**254780164**	537021.9.peg.48	ATP-dependent Clp protease adaptor protein ClpS	ATP-dependent Clp protease adaptor protein ClpS	ATP-dependent Clp protease adaptor protein ClpS	wrong start point? But start from??
**254780165**	537021.9.peg.49	hypothetical protein	hypothetical protein	unknown	correct protein, unknown function
**254780166**	537021.9.peg.50	hydrolase protein	probable hydrolase protein	hydrolase	
**254780167**	537021.9.peg.51	hypothetical protein	hypothetical protein	zinc-finger	need to check out function
**254780168**	537021.9.peg.52	monooxygenase FAD-binding protein	Salicylate hydroxylase (EC 1.14.13.1)	salicylate hydroxylase (EC 1.14.13.1)	
**254780169**	537021.9.peg.53	hypothetical protein	hypothetical protein	unknown	wrong gene prediction, only 54 aa
**254780170**	537021.9.peg.55	cationic amino acid ABC transporter, periplasmic binding protein	Glutamate Aspartate periplasmic binding protein precursor GltI (TC 3.A.1.3.4)	ABC-type importer for general L-amino acids, substrate binding component	has signal peptide
**254780171**	537021.9.peg.56	ABC transporter membrane spanning protein (amino acid)	Glutamate Aspartate transport system permease protein GltJ (TC 3.A.1.3.4)	ABC-type importer for general L-amino acids, transmembrane component	transmembrane protein
**254780172**	537021.9.peg.57	general L-amino acid transport system permease protein	Glutamate Aspartate transport system permease protein GltK (TC 3.A.1.3.4)	ABC-type importer for general L-amino acids, transmembrane component	transmembrane protein
**254780173**	537021.9.peg.58	ABC transporter related protein	Amino acid ABC transporter, ATP-binding protein	ABC-type importer for general L-amino acids, ATPase component	
**254780174**	537021.9.peg.59	putative oxidoreductase protein	PUTATIVE OXIDOREDUCTASE PROTEIN	oxidoreductase protein in electron transport chain complex I	start point wrong prediction
**254780175**	537021.9.peg.61	hypothetical protein	hypothetical protein	unknown	wrong gene prediction, only 38 aa, and find nothing in NR
**254780176**	537021.9.peg.62	hypothetical protein	hypothetical protein	unknown	No censensus prediction
**254780177**	537021.9.peg.63	lipoyltransferase	Octanoate-[acyl-carrier-protein]-protein-N-octanoyltransferase	lipoyltransferase (EC:2.3.1.181)	
**254780178**	537021.9.peg.64	queuine tRNA-ribosyltransferase	tRNA-guanine transglycosylase (EC 2.4.2.29)	queuine tRNA-ribosyltransferase (EC 2.4.2.29)	
**254780179**	537021.9.peg.65	hypothetical protein	hypothetical protein	unknown	transmembrane protein
**254780180**	537021.9.peg.66	S-adenosylmethionine:tRNA ribosyltransferase-isomerase	S-adenosylmethionine:tRNA ribosyltransferase-isomerase (EC 5.-.-.-)	S-adenosylmethionine:tRNA ribosyltransferase-isomerase (EC 5.-.-.-)	
**254780181**	537021.9.peg.67	phosphopantetheine adenylyltransferase	Phosphopantetheine adenylyltransferase (EC 2.7.7.3)	phosphopantetheine adenylyltransferase (EC 2.7.7.3)	
**254780182**	537021.9.peg.68	DNA gyrase subunit A	DNA gyrase subunit A (EC 5.99.1.3)	DNA gyrase subunit A (EC 5.99.1.3)	
**254780183**	537021.9.peg.69	single-strand binding protein (ssb)	Single-stranded DNA-binding protein	single-strand binding protein (ssb)	
**254780184**	537021.9.peg.70	excinuclease ABC subunit A	Excinuclease ABC subunit A	excinuclease ABC subunit A, uvrA	
**254780185**	537021.9.peg.71	glutamine synthetase protein	Glutamine synthetase type I (EC 6.3.1.2)	glutamine synthetase (EC 6.3.1.2)	
**254780186**	537021.9.peg.72	glutaminase	Glutaminase (EC 3.5.1.2)	glutaminase (EC 3.5.1.2)	
**254780187**	537021.9.peg.73	30S ribosomal protein S4	SSU ribosomal protein S4p (S9e)	30S ribosomal protein S4	
**N/A**	537021.9.peg.74	N/A	Ribonucleotide reductase of class Ia (aerobic), beta subunit (EC 1.17.4.1)	ribonucleotide-diphosphate reductase subunit beta	1/2 of a protein
**N/A**	537021.9.peg.75	N/A	Ribonucleotide reductase of class Ia (aerobic), beta subunit (EC 1.17.4.1)	ribonucleotide-diphosphate reductase subunit beta	1/2 of a protein
**254780189**	537021.9.peg.76	hypothetical protein	hypothetical protein	unknown	
**254780190**	537021.9.peg.77	fumarate hydratase	Fumarate hydratase class II (EC 4.2.1.2)	fumarate hydratase (EC 4.2.1.2)	
**254780191**	537021.9.peg.78	alanyl-tRNA synthetase	Alanyl-tRNA synthetase (EC 6.1.1.7)	alanyl-tRNA synthetase (EC 6.1.1.7)	
**255764460**	537021.9.peg.79	recombinase A	RecA protein	recombinase A	
**254780193**	537021.9.peg.81	lipid A ABC exporter family, fused ATPase and inner membrane subunits	hypothetical protein	ABC-type exporter for multiple drug and lipid, transmembrane and ATPase component	transmembrane protein
**254780194**	537021.9.peg.83	50S ribosomal protein L31	LSU ribosomal protein L31p	50S ribosomal protein L31	
**254780195**	537021.9.peg.84	CTP synthetase	CTP synthase (EC 6.3.4.2)	CTP synthetase (EC 6.3.4.2)	

**254780196**	537021.9.peg.85	hypothetical protein	hypothetical protein	protein translocase (for protein to pass the inner membrane)	transmembrane protein
**254780197**	537021.9.peg.86	triosephosphate isomerase protein	Triosephosphate isomerase (EC 5.3.1.1)	triosephosphate isomerase protein (EC 5.3.1.1)	
**254780198**	N/A	hypothetical protein	N/A	unknown	transmembrane protein
**254780199**	537021.9.peg.87	outer membrane lipoprotein	hypothetical protein	outermembrane lipoprotein	has signal peptide
**254780200**	537021.9.peg.88	ferrochelatase	Ferrochelatase, protoheme ferro-lyase (EC 4.99.1.1)	ferrochelatase (EC 4.99.1.1)	
**254780201**	537021.9.peg.89	hypothetical protein	hypothetical protein	Lipopolysaccharide biosynthesis protein	
**254780202**	537021.9.peg.90	hypothetical protein	Queuosine Biosynthesis QueE Radical SAM	organic radical activating enzymes	
**N/A**	537021.9.peg.91	N/A	Putative deoxyribonuclease YcfH	hydrolase, just piece of protein 254780214	just piece of protein 254780214
**254780203**	537021.9.peg.92	hypothetical protein	hypothetical protein	unknown	
**254780204**	537021.9.peg.93	hypothetical protein	hypothetical protein	unknown	transmembrane protein
**254780205**	537021.9.peg.94	prenyltransferase	4-hydroxybenzoate polyprenyltransferase (EC 2.5.1.-)	4-hydroxybenzoate polyprenyltransferase (EC 2.5.1.-)	transmembrane protein
**254780206**	537021.9.peg.95	phosphoribosylamine--glycine ligase	Phosphoribosylamine--glycine ligase (EC 6.3.4.13)	phosphoribosylamine--glycine ligase (EC 6.3.4.13)	
**254780207**	537021.9.peg.96	hypothetical protein	hypothetical protein	unknown	has signal peptide
**254780209**	537021.9.peg.99	hypothetical protein	hypothetical protein	unknown	has signal peptide
**254780210**	537021.9.peg.100	hypothetical protein	hypothetical protein	unknown	
**254780212**	537021.9.peg.101	bacterioferritin comigratory protein	Thiol peroxidase, Bcp-type (EC 1.11.1.15)	bacterioferritin comigratory protein/peroxiredoxin (EC 1.11.1.15)	bacterioferritin comigratory protein = peroxiredoxin
**254780213**	537021.9.peg.102	hypothetical protein	hypothetical protein	unknown	
**254780214**	537021.9.peg.103	metal-dependent hydrolase protein	Metal-dependent hydrolases of the beta-lactamase superfamily I; PhnP protein	metal-dependent hydrolase protein	
**254780215**	537021.9.peg.104	hypothetical protein	Putative deoxyribonuclease YcfH	Dnase	
**255764461**	537021.9.peg.105	methionyl-tRNA synthetase	Methionyl-tRNA synthetase (EC 6.1.1.10)	methionyl-tRNA synthetase (EC 6.1.1.10)	
**254780217**	537021.9.peg.106	DNA polymerase III subunit delta'	DNA polymerase III delta prime subunit (EC 2.7.7.7)	DNA polymerase III subunit delta' (EC 2.7.7.7)	
**254780218**	537021.9.peg.107	thymidylate kinase	Thymidylate kinase (EC 2.7.4.9)	thymidylate kinase (EC 2.7.4.9)	
**254780219**	537021.9.peg.108	hypothetical protein	hypothetical protein	unknown	
**254780220**	N/A	hypothetical protein	N/A	unknown	has signal peptide
**254780221**	537021.9.peg.110	hypothetical protein	hypothetical protein	unknown	has signal peptide
**254780222**	537021.9.peg.111	DNA topoisomerase IV subunit B	Topoisomerase IV subunit B (EC 5.99.1.-)	DNA topoisomerase IV subunit B (EC 5.99.1.-)	
**254780223**	537021.9.peg.112	ABC transporter permease	hypothetical protein	permease	transmembrane protein
**254780224**	537021.9.peg.113	hypothetical protein	hypothetical protein	unknown	
**254780225**	537021.9.peg.114	peptidyl-tRNA hydrolase	Peptidyl-tRNA hydrolase (EC 3.1.1.29)	peptidyl-tRNA hydrolase (EC 3.1.1.29)	
**254780226**	537021.9.peg.115	translation-associated GTPase	GTP-binding and nucleic acid-binding protein YchF	translation-associated GTPase	
**254780227**	537021.9.peg.117	pyrophosphate--fructose-6-phosphate 1-phosphotransferase	Pyrophosphate--fructose 6-phosphate 1-phosphotransferase, alpha subunit (EC 2.7.1.90)	pyrophosphate--fructose-6-phosphate 1-phosphotransferase subunit alpha (EC 2.7.1.90)	
**254780228**	537021.9.peg.118	hypothetical protein	Integral membrane protein	unknown	transmembrane protein
**254780229**	537021.9.peg.119	hypothetical protein	hypothetical protein	DNA helicase	
**254780230**	537021.9.peg.120	hypothetical protein	hypothetical conserved membrane protein	unknown	transmembrane protein
**254780231**	537021.9.peg.121	hypothetical protein	hypothetical protein	unknown	
**254780232**	537021.9.peg.122	inorganic pyrophosphatase	Inorganic pyrophosphatase (EC 3.6.1.1)	inorganic pyrophosphatase (EC 3.6.1.1)	
**254780233**	537021.9.peg.123	GTP-binding protein	GTP-binding protein TypA/BipA	GTPase, TypA involved in stress response	
**254780234**	537021.9.peg.124	argininosuccinate synthase	Argininosuccinate synthase (EC 6.3.4.5)	argininosuccinate synthase (EC 6.3.4.5)	
**254780235**	537021.9.peg.125	hypothetical protein	hypothetical protein	unknown	transmembrane protein
**254780236**	537021.9.peg.126	50S ribosomal protein L17	LSU ribosomal protein L17p	50S ribosomal protein L17	
**254780237**	537021.9.peg.127	DNA-directed RNA polymerase subunit alpha	DNA-directed RNA polymerase alpha subunit (EC 2.7.7.6)	DNA-directed RNA polymerase subunit alpha (EC 2.7.7.6)	
**254780238**	537021.9.peg.129	30S ribosomal protein S11	SSU ribosomal protein S11p (S14e)	30S ribosomal protein S11	
**254780239**	537021.9.peg.130	30S ribosomal protein S13	SSU ribosomal protein S13p (S18e)	30S ribosomal protein S13	
**254780240**	537021.9.peg.131	adenylate kinase	Adenylate kinase (EC 2.7.4.3)	adenylate kinase (EC 2.7.4.3)	
**254780241**	537021.9.peg.132	preprotein translocase subunit SecY	Preprotein translocase secY subunit (TC 3.A.5.1.1)	preprotein translocase subunit SecY	transmembrane protein
**254780242**	537021.9.peg.133	50S ribosomal protein L15	LSU ribosomal protein L15p (L27Ae)	50S ribosomal protein L15	
**254780243**	537021.9.peg.134	50S ribosomal protein L30	LSU ribosomal protein L30p (L7e)	50S ribosomal protein L30	

**254780244**	537021.9.peg.135	30S ribosomal protein S5	SSU ribosomal protein S5p (S2e)	30S ribosomal protein S5	
**254780245**	537021.9.peg.136	50S ribosomal protein L18	LSU ribosomal protein L18p (L5e)	50S ribosomal protein L18	
**254780246**	537021.9.peg.137	50S ribosomal protein L6	LSU ribosomal protein L6p (L9e)	50S ribosomal protein L6	
**254780247**	537021.9.peg.138	30S ribosomal protein S8	SSU ribosomal protein S8p (S15Ae)	30S ribosomal protein S8	
**254780248**	537021.9.peg.139	30S ribosomal protein S14	SSU ribosomal protein S14p (S29e) Zinc-independent	30S ribosomal protein S14	
**254780249**	537021.9.peg.140	50S ribosomal protein L5	LSU ribosomal protein L5p (L11e)	50S ribosomal protein L5	
**254780250**	537021.9.peg.141	50S ribosomal protein L24	LSU ribosomal protein L24p (L26e)	50S ribosomal protein L24	
**254780251**	537021.9.peg.142	50S ribosomal protein L14	LSU ribosomal protein L14p (L23e)	50S ribosomal protein L14	
**254780252**	537021.9.peg.143	30S ribosomal protein S17	SSU ribosomal protein S17p (S11e)	30S ribosomal protein S17	
**254780253**	537021.9.peg.144	ribosomal protein L29	LSU ribosomal protein L29p (L35e)	ribosomal protein L29	
**254780254**	537021.9.peg.145	50S ribosomal protein L16	LSU ribosomal protein L16p (L10e)	50S ribosomal protein L16	
**254780255**	537021.9.peg.146	30S ribosomal protein S3	SSU ribosomal protein S3p (S3e)	30S ribosomal protein S3	
**254780256**	537021.9.peg.147	50S ribosomal protein L22	LSU ribosomal protein L22p (L17e)	50S ribosomal protein L22	
**254780257**	537021.9.peg.148	SSU ribosomal protein S19P	SSU ribosomal protein S19p (S15e)	SSU ribosomal protein S19P	
**254780258**	537021.9.peg.149	50S ribosomal protein L2	LSU ribosomal protein L2p (L8e)	50S ribosomal protein L2	
**254780259**	537021.9.peg.150	50S ribosomal protein L23	LSU ribosomal protein L23p (L23Ae)	50S ribosomal protein L23	
**254780260**	537021.9.peg.151	50S ribosomal protein L4	LSU ribosomal protein L4p (L1e)	50S ribosomal protein L4	
**254780261**	537021.9.peg.152	50S ribosomal protein L3	LSU ribosomal protein L3p (L3e)	50S ribosomal protein L3	
**254780262**	537021.9.peg.153	30S ribosomal protein S10	SSU ribosomal protein S10p (S20e)	30S ribosomal protein S10	
**254780263**	537021.9.peg.154	translation elongation factor Tu	Translation elongation factor Tu	translation elongation factor Tu	
**254780264**	537021.9.peg.155	elongation factor G	Translation elongation factor G	elongation factor G	
**254780265**	537021.9.peg.156	30S ribosomal protein S7	SSU ribosomal protein S7p (S5e)	30S ribosomal protein S7	
**254780266**	537021.9.peg.157	30S ribosomal protein S12	SSU ribosomal protein S12p (S23e)	30S ribosomal protein S12	
**254780267**	537021.9.peg.158	acetyl-CoA carboxylase biotin carboxyl carrier protein subunit	Biotin carboxyl carrier protein of acetyl-CoA carboxylase	acetyl-CoA carboxylase biotin carboxyl carrie	
**254780268**	537021.9.peg.159	acetyl-CoA carboxylase biotin carboxylase subunit	Biotin carboxylase of acetyl-CoA carboxylase (EC 6.3.4.14)	acetyl-CoA carboxylase biotin carboxylase (EC 6.3.4.14)	
**254780269**	537021.9.peg.160	leucyl/phenylalanyl-tRNA--protein transferase	Leucyl/phenylalanyl-tRNA--protein transferase (EC 2.3.2.6)	leucyl/phenylalanyl-tRNA--protein transferase (EC 2.3.2.6)	
**254780270**	537021.9.peg.161	ATP-dependent protease La	ATP-dependent protease La (EC 3.4.21.53) Type I	ATP-dependent protease La (EC 3.4.21.53)	
**254780271**	537021.9.peg.162	ATP-dependent protease ATP-binding subunit ClpX	ATP-dependent Clp protease ATP-binding subunit ClpX	ATP-dependent protease ATP-binding subunit ClpX	
**254780272**	537021.9.peg.163	ATP-dependent Clp protease proteolytic subunit	ATP-dependent Clp protease proteolytic subunit (EC 3.4.21.92)	ATP-dependent Clp protease proteolytic subunit (EC 3.4.21.92)	
**254780273**	537021.9.peg.164	putative ABC transporter ATP-binding protein	ABC transporter, ATP-binding protein	ABC-type ATPase, function as regulator for virulence gene, chvD	
**254780274**	537021.9.peg.165	lysophospholipase protein	hypothetical protein	lysophospholipase protein	
**254780275**	537021.9.peg.167	SsrA-binding protein	tmRNA-binding protein SmpB	SsrA-binding protein	
**254780276**	537021.9.peg.168	dihydrodipicolinate synthase	Dihydrodipicolinate synthase (EC 4.2.1.52)	dihydrodipicolinate synthase (EC 4.2.1.52)	
**254780277**	537021.9.peg.169	DNA polymerase I	DNA polymerase I (EC 2.7.7.7)	DNA polymerase I	
**254780278**	537021.9.peg.170	phosphoglyceromutase	Phosphoglycerate mutase (EC 5.4.2.1)	phosphoglyceromutase (EC 5.4.2.1)	
**254780279**	537021.9.peg.171	dihydrodipicolinate reductase	Dihydrodipicolinate reductase (EC 1.3.1.26)	dihydrodipicolinate reductase (EC 1.3.1.26)	
**254780280**	537021.9.peg.172	glucokinase	Glucokinase (EC 2.7.1.2)	glucokinase (EC 2.7.1.2)	
**254780281**	537021.9.peg.173	cytochrome-c oxidase assembly factor protein	Cytochrome oxidase biogenesis protein Sco1/SenC/PrrC, putative copper metallochaperone	cytochrome-c oxidase assembly factor protein	has signal peptide
**254780282**	537021.9.peg.174	putative transcription regulator protein	Transcriptional regulator, LysR family	putative transcription regulator protein	
**254780283**	537021.9.peg.175	thioredoxin reductase (NADPH) protein	Thioredoxin reductase (EC 1.8.1.9)	thioredoxin reductase (NADPH) protein (EC 1.8.1.9)	
**254780284**	537021.9.peg.176	hypothetical protein	hypothetical protein	unknown	transmembrane protein
**254780285**	537021.9.peg.177	arginyl-tRNA synthetase	Arginyl-tRNA synthetase (EC 6.1.1.19)	arginyl-tRNA synthetase (EC 6.1.1.19)	
**254780286**	537021.9.peg.178	deoxyguanosinetriphosphate triphosphohydrolase-like protein	Deoxyguanosinetriphosphate triphosphohydrolase (EC 3.1.5.1)	dGTP triphosphohydrolase (EC 3.1.5.1)	
**254780287**	537021.9.peg.179	iron-sulfur cluster assembly accessory protein	probable iron binding protein from the HesB_IscA_SufA family	iron-sulfur cluster assembly accessory protein	
**254780288**	537021.9.peg.180	exodeoxyribonuclease III protein	Exodeoxyribonuclease III (EC 3.1.11.2)	exodeoxyribonuclease III protein (EC 3.1.11.2)	
**254780289**	537021.9.peg.181	RNA polymerase sigma factor RpoD	RNA polymerase sigma factor RpoD	RNA polymerase sigma factor RpoD	

**254780290**	537021.9.peg.182	quinone oxidoreductase	Quinone oxidoreductase (EC 1.6.5.5)	quinone oxidoreductase	
**254780291**	537021.9.peg.183	hypothetical protein	hypothetical protein	unknown	transmembrane protein
**254780292**	537021.9.peg.184	50S ribosomal protein L13	LSU ribosomal protein L13p (L13Ae)	50S ribosomal protein L13	start point might be wrong
**254780293**	537021.9.peg.185	30S ribosomal protein S9	SSU ribosomal protein S9p (S16e)	30S ribosomal protein S9	
**254780294**	537021.9.peg.187	N-acetyl-gamma-glutamyl-phosphate reductase	N-acetyl-gamma-glutamyl-phosphate reductase (EC 1.2.1.38)	N-acetyl-gamma-glutamyl-phosphate reductase (EC 1.2.1.38)	
**254780295**	537021.9.peg.188	tRNA pseudouridine synthase A	tRNA pseudouridine synthase A (EC 4.2.1.70)	tRNA pseudouridine synthase A (EC 4.2.1.70)	
**255764462**	537021.9.peg.189	methionyl-tRNA formyltransferase	Methionyl-tRNA formyltransferase (EC 2.1.2.9)	methionyl-tRNA formyltransferase (EC 2.1.2.9)	
**254780297**	537021.9.peg.190	peptide deformylase	Peptide deformylase (EC 3.5.1.88)	peptide deformylase (EC 3.5.1.88)	
**254780298**	537021.9.peg.192	putative transmembrane protein	hypothetical multidrug efflux transporter protein	unknown	transmembrane protein
**254780299**	537021.9.peg.193	acyl-CoA dehydrogenase protein	Acyl-CoA dehydrogenase (EC 1.3.99.3)	acyl-CoA dehydrogenase protein (EC 1.3.99.3)	
**254780300**	537021.9.peg.194	aspartate carbamoyltransferase catalytic subunit	Aspartate carbamoyltransferase (EC 2.1.3.2)	aspartate carbamoyltransferase catalytic subunit (EC 2.1.3.2)	
**254780301**	537021.9.peg.195	dihydroorotase	Dihydroorotase (EC 3.5.2.3)	dihydroorotase (EC 3.5.2.3)	
**254780302**	537021.9.peg.196	putative glycerol-3-phosphate acyltransferase PlsY	Acyl-phosphate:glycerol-3-phosphate O-acyltransferase PlsY	glycerol-3-phosphate acyltransferas	transmembrane protein
**254780303**	537021.9.peg.197	DNA protecting protein DprA	putative smf protein	DNA protecting protein DprA	1/3 of the protein
**254780304**	N/A	DNA protecting protein DprA	N/A	DNA protecting protein DprA	1/3 of the protein
**254780305**	537021.9.peg.198	hypothetical protein	hypothetical protein	DNA protecting protein DprA	1/3 of the protein
**254780306**	537021.9.peg.199	DNA topoisomerase I	DNA topoisomerase I (EC 5.99.1.2)	DNA topoisomerase I (EC 5.99.1.2)	
**254780307**	537021.9.peg.200	aspartate-semialdehyde dehydrogenase	Aspartate-semialdehyde dehydrogenase (EC 1.2.1.11)	aspartate-semialdehyde dehydrogenase (EC 1.2.1.11)	
**254780308**	537021.9.peg.201	putative membrane-bound lytic murein transglycosylase signal peptide protein	Membrane-bound lytic murein transglycosylase B precursor (EC 3.2.1.-)	lytic murein transglycosylase protein	has signal peptide
**254780309**	537021.9.peg.202	comF family protein	Competence protein comF	Competence protein comF	1/2 of the protein
**254780310**	537021.9.peg.203	hypothetical protein	hypothetical protein	Competence protein comF	1/2 of the protein
**254780311**	537021.9.peg.205	hypothetical protein	Expressed protein	peroxiredoxin	
**254780312**	537021.9.peg.206	response regulator receiver protein	Response regulator	two component system regulator protein	
**254780313**	537021.9.peg.207	ribosomal RNA methyltransferase RrmJ/FtsJ	Cell division protein FtsJ / Ribosomal RNA large subunit methyltransferase E (EC 2.1.1.-)	ribosomal RNA methyltransferase RrmJ/FtsJ (EC 2.1.1.-)	
**254780314**	537021.9.peg.208	outer membrane protein	porin outer membrane protein	outer membrane protein	has signal peptide
**254780315**	537021.9.peg.209	hypothetical protein	hypothetical protein	unknown	
**254780316**	537021.9.peg.210	phenylalanyl-tRNA synthetase subunit beta	Phenylalanyl-tRNA synthetase beta chain (EC 6.1.1.20)	phenylalanyl-tRNA synthetase subunit beta (EC 6.1.1.20)	
**254780317**	537021.9.peg.211	phenylalanyl-tRNA synthetase, alpha subunit	Phenylalanyl-tRNA synthetase alpha chain (EC 6.1.1.20)	phenylalanyl-tRNA synthetase, alpha subunit (EC 6.1.1.20)	
**254780318**	537021.9.peg.212	ribosomal protein L20	LSU ribosomal protein L20p	ribosomal protein L20	
**254780319**	537021.9.peg.213	50S ribosomal protein L35	LSU ribosomal protein L35p	50S ribosomal protein L35	
**254780320**	537021.9.peg.214	translation initiation factor IF-3	Translation initiation factor 3	translation initiation factor IF-3	
**254780321**	537021.9.peg.215	GTP-binding protein LepA	Translation elongation factor LepA	GTP-binding protein LepA	
**254780322**	537021.9.peg.216	SAM-dependent methyltransferase protein	probable SAM-dependent methyltransferase protein	SAM-dependent methyltransferase protein	
**254780323**	537021.9.peg.217	ATP/ADP translocase	Nucleotide related transporter , NTT family	ATP/ADP translocase	transmembrane protein
**254780324**	537021.9.peg.219	hypothetical protein	hypothetical protein	unknown	transmembrane protein
**254780325**	537021.9.peg.220	mutator MutT protein	5-methyl-dCTP pyrophosphohydrolase (EC 3.6.1.-)	mutator MutT protein (EC 3.6.1.-)	
**254780326**	537021.9.peg.221	bifunctional ornithine acetyltransferase/N-acetylglutamate synthase protein	Glutamate N-acetyltransferase (EC 2.3.1.35) / N-acetylglutamate synthase (EC 2.3.1.1)	bifunctional ornithine acetyltransferase/N-acetylglutamate synthase	
**254780327**	537021.9.peg.222	preprotein translocase subunit SecA	Protein export cytoplasm protein SecA ATPase RNA helicase (TC 3.A.5.1.1)	preprotein translocase subunit SecA	
**254780328**	537021.9.peg.223	UDP-glucose 4-epimerase	UDP-glucose 4-epimerase (EC 5.1.3.2)	UDP-glucose 4-epimerase (EC 5.1.3.2)	
**254780329**	537021.9.peg.225	30S ribosomal protein S6	SSU ribosomal protein S6p	30S ribosomal protein S6	
**254780330**	537021.9.peg.226	30S ribosomal protein S18	SSU ribosomal protein S18p	30S ribosomal protein S18	
**254780331**	537021.9.peg.228	50S ribosomal protein L9	LSU ribosomal protein L9p	50S ribosomal protein L9	
**254780332**	537021.9.peg.229	replicative DNA helicase	Replicative DNA helicase (EC 3.6.1.-)	replicative DNA helicase (EC 3.6.1.-)	
**254780333**	537021.9.peg.230	alanine racemase	Alanine racemase (EC 5.1.1.1)	alanine racemase (EC 5.1.1.1)	
**254780334**	537021.9.peg.231	DNA repair protein RadA	DNA repair protein RadA	DNA repair protein RadA	

**N/A**	537021.9.peg.232	N/A	hypothetical protein	unknown	
**254780335**	N/A	colicin V production protein	N/A	colicin V production protein	transmembrane protein
**254780336**	537021.9.peg.234	amidophosphoribosyltransferase	Amidophosphoribosyltransferase (EC 2.4.2.14)	amidophosphoribosyltransferase (EC 2.4.2.14)	
**254780337**	537021.9.peg.235	oxidoreductase protein	putative short-chain dehydrogenase/reductase	oxidoreductase protein	
**254780338**	537021.9.peg.236	hypothetical protein	hypothetical protein	unknown	transmembrane protein
**N/A**	537021.9.peg.237	N/A	hypothetical protein	unknown	transmembrane protein
**254780339**	537021.9.peg.238	hypothetical protein	hypothetical protein	unknown	
**254780340**	537021.9.peg.239	proline/glycine betaine ABC transporter, ATP-binding protein	L-proline glycine betaine ABC transport system permease protein ProV (TC 3.A.1.12.1)	ABC-type importer for Choline (vitamin Bp), ATPase component	
**254780341**	537021.9.peg.240	proline/glycine betaine ABC transporter, permease protein	L-proline glycine betaine ABC transport system permease protein ProW (TC 3.A.1.12.1)	ABC-type importer for Choline (vitamin Bp), transmembrane component	transmembrane protein
**254780342**	537021.9.peg.241	substrate-binding region of ABC-type glycine betaine transport system	putative glycine betaine-binding ABC transporter protein	ABC-type importer for Choline (vitamin Bp), substrate binding component	has signal peptide
**254780343**	537021.9.peg.242	probable cation efflux protein	Cobalt-zinc-cadmium resistance protein	cation efflux protein	transmembrane protein
**254780344**	537021.9.peg.243	7-cyano-7-deazaguanine reductase	NADPH dependent preQ0 reductase	7-cyano-7-deazaguanine reductase	
**254780345**	537021.9.peg.244	transcription antitermination protein NusB	Transcription termination protein NusB	transcription antitermination protein NusB	
**254780346**	537021.9.peg.245	riboflavin synthase subunit beta	6,7-dimethyl-8-ribityllumazine synthase (EC 2.5.1.9)	riboflavin synthase subunit beta (EC 2.5.1.9)	
**254780347**	537021.9.peg.246	riboflavin synthase subunit alpha	Riboflavin synthase alpha chain (EC 2.5.1.9)	riboflavin synthase subunit alpha (EC 2.5.1.9)	
**254780348**	537021.9.peg.247	5-amino-6-(5-phosphoribosylamino)uracil reductase/diaminohydroxyphosphoribosylaminopyrimidine	Diaminohydroxyphosphoribosylaminopyrimidine deaminase (EC 3.5.4.26) / 5-amino-6-(5-phosphoribosylamino)uracil reductase (EC 1.1.1.193)	5-amino-6-(5-phosphoribosylamino)uracil reductase/diaminohydroxyphosphoribosylaminopyrimidine (EC 3.5.4.26) (EC 1.1.1.193)	
**254780349**	537021.9.peg.249	serine hydroxymethyltransferase	Serine hydroxymethyltransferase (EC 2.1.2.1)	serine hydroxymethyltransferase (EC 2.1.2.1)	
**254780350**	537021.9.peg.250	hypothetical protein	hypothetical protein	unknown	has signal peptide
**254780351**	537021.9.peg.251	MarR family transcriptional regulator	Transcriptional regulator, MarR family	MarR family transcriptional regulator	
**255764463**	537021.9.peg.253	delta-aminolevulinic acid dehydratase	Porphobilinogen synthase (EC 4.2.1.24)	delta-aminolevulinic acid dehydratase	
**254780353**	537021.9.peg.255	DNA topoisomerase IV subunit A	Topoisomerase IV subunit A (EC 5.99.1.-)	DNA topoisomerase IV subunit A (EC 5.99.1.-)	
**254780354**	537021.9.peg.256	hypothetical protein	hypothetical protein	unknown	transmembrane protein
**254780355**	537021.9.peg.257	type II citrate synthase	Citrate synthase (si) (EC 2.3.3.1)	type II citrate synthase (EC 2.3.3.1)	
**254780356**	537021.9.peg.258	hypothetical protein	hypothetical protein	DNA uptake, ComEC/Rec2-related protein	transmembrane protein
**254780357**	537021.9.peg.259	hypothetical protein	DNA internalization-related competence protein ComEC/Rec2	DNA uptake, ComEC/Rec2-related protein	transmembrane protein
**255764464**	537021.9.peg.260	Cytidine/deoxycytidylate deaminase, zinc-binding region	tRNA-specific adenosine-34 deaminase (EC 3.5.4.-)	adenosine deaminasese (EC 3.5.4.-)	
**254780359**	537021.9.peg.261	GTP cyclohydrolase II protein (riboflavin biosynthesis)	3,4-dihydroxy-2-butanone 4-phosphate synthase / GTP cyclohydrolase II (EC 3.5.4.25)	GTP cyclohydrolase II protein (EC 3.5.4.25)	
**254780360**	537021.9.peg.262	protoheme IX farnesyltransferase	Heme O synthase, protoheme IX farnesyltransferase (EC 2.5.1.-) COX10-CtaB	protoheme IX farnesyltransferase (EC 2.5.1.-)	transmembrane protein
**254780361**	537021.9.peg.263	ribosomal protein L32	LSU ribosomal protein L32p	ribosomal protein L32	
**255764465**	537021.9.peg.264	Na+/H+ antiporter NhaA	Na+/H+ antiporter NhaA type	Na+/H+ antiporter NhaA	transmembrane protein
**254780363**	537021.9.peg.265	ferredoxin-NADP+ reductase protein	Flavodoxin reductases (ferredoxin-NADPH reductases) family 1	ferredoxin-NADP+ reductase protein	
**254780364**	537021.9.peg.266	ferredoxin-NADP+ reductase protein	Ferredoxin--NADP(+) reductase (EC 1.18.1.2)	ferredoxin-NADP+ reductase protein	
**254780365**	537021.9.peg.267	UTP-glucose-1-phosphate uridylyltransferase protein	UTP--glucose-1-phosphate uridylyltransferase (EC 2.7.7.9)	UTP-glucose-1-phosphate uridylyltransferase (EC 2.7.7.9)	
**255764466**	537021.9.peg.268	hypothetical protein	Periplasmic thiol:disulfide oxidoreductase DsbB, required for DsbA reoxidation	Disulfide bond formation protein DsbB	transmembrane protein
**254780367**	537021.9.peg.269	ribonuclease protein	Ribonuclease BN (EC 3.1.-.-)	ribonuclease protein (EC 3.1.-.-)	transmembrane protein
**254780368**	537021.9.peg.270	electron transfer flavoprotein beta subunit	Electron transfer flavoprotein, beta subunit	electron transfer flavoprotein beta subunit	
**254780369**	537021.9.peg.271	Electron transfer flavoprotein alpha subunit	Electron transfer flavoprotein, alpha subunit	Electron transfer flavoprotein alpha subunit	
**254780370**	537021.9.peg.272	argininosuccinate lyase	Argininosuccinate lyase (EC 4.3.2.1)	argininosuccinate lyase (EC 4.3.2.1)	
**254780371**	537021.9.peg.273	diaminopimelate decarboxylase protein	Diaminopimelate decarboxylase (EC 4.1.1.20)	diaminopimelate decarboxylase protein (EC 4.1.1.20)	
**254780372**	537021.9.peg.274	flagellar biosynthesis protein FliP	Flagellar biosynthesis protein FliP	flagellar biosynthesis protein FliP	transmembrane protein
**254780373**	537021.9.peg.275	flagellar basal body-associated protein FliL	Flagellar biosynthesis protein fliL	flagellar basal body-associated protein FliL	transmembrane protein
**254780374**	537021.9.peg.276	flagellar basal body L-ring protein	Flagellar L-ring protein FlgH	flagellar basal body L-ring protein	has signal peptide
**254780375**	537021.9.peg.277	hypothetical protein	hypothetical protein	unknown	has signal peptide

**254780376**	537021.9.peg.278	flagellar basal body P-ring protein	Flagellar P-ring protein FlgI	flagellar basal body P-ring protein	has signal peptide
**254780377**	537021.9.peg.279	flagellar basal body P-ring biosynthesis protein FlgA	Flagellar basal-body P-ring formation protein flgA	flagellar basal body P-ring biosynthesis protein	has signal peptide
**254780378**	537021.9.peg.280	flagellar basal body rod protein FlgG	Flagellar basal-body rod protein FlgG	flagellar basal body rod protein FlgG	extracellular, flagellar component, exported by flagellar assembly machinery
**254780379**	537021.9.peg.281	flagellar hook-basal body protein FliE	Flagellar hook-basal body complex protein FliE	flagellar hook-basal body protein FliE	periplasmic, flagellar component, transported by flagellar
**254780380**	537021.9.peg.282	flagellar basal body rod protein FlgC	Flagellar basal-body rod protein FlgC	flagellar basal body rod protein FlgC	periplasmic, flagellar component, transported by flagellar
**254780381**	537021.9.peg.283	flagellar basal body rod protein FlgB	Flagellar basal-body rod protein FlgB	flagellar basal body rod protein FlgB	periplasmic, flagellar component, transported by flagellar
**254780382**	537021.9.peg.284	ATP dependent RNA helicase protein	ATP-dependent RNA helicase Atu1833	ATP dependent RNA helicase protein	
**254780383**	537021.9.peg.285	endonuclease III	Endonuclease III (EC 4.2.99.18)	endonuclease III (EC 4.2.99.18)	
**254780384**	537021.9.peg.286	serralysin	hypothetical protein	zinc-dependant protease	
**255764467**	537021.9.peg.287	Type I secretion system ATPase, PrtD	hypothetical protein	Type I secretion system ATPase and permease, PrtD, ABC-exporter	transmembrane protein
**254780386**	537021.9.peg.288	Type I secretion membrane fusion protein, HlyD	Alkaline protease secretion protein AprE	Type I secretion membrane fusion protein, HlyD, ABC-type-exporter	transmembrane protein
**254780387**	537021.9.peg.289	C4-dicarboxylate transporter DctA	C4-dicarboxylate transport protein	Na+/H+-dicarboxylate symporter	transmembrane protein
**254780388**	537021.9.peg.290	hypothetical protein	hypothetical protein	von Willebrand factor type A fused with TadE/F involved in pilus assembly	transmembrane protein, but might be processed by cpaA and become periplasmic
**254780389**	537021.9.peg.291	hypothetical protein	hypothetical protein	unknown	
**254780390**	537021.9.peg.292	nucleoside diphosphate kinase	Nucleoside diphosphate kinase (EC 2.7.4.6)	nucleoside diphosphate kinase (EC 2.7.4.6)	
**254780391**	537021.9.peg.293	DNA polymerase III subunit chi	DNA polymerase III chi subunit (EC 2.7.7.7)	DNA polymerase III subunit chi (EC 2.7.7.7)	
**254780392**	537021.9.peg.294	leucyl aminopeptidase	Cytosol aminopeptidase PepA (EC 3.4.11.1)	leucyl aminopeptidase (EC 3.4.11.1)	
**255764468**	537021.9.peg.295	permease protein	hypothetical protein	ABC-type exporter for lipopolysaccharide, transmembrane component	transmembrane protein
**255764469**	537021.9.peg.296	putative permease protein	putative permease protein	ABC-type exporter for lipopolysaccharide, transmembrane component	transmembrane protein
**254780395**	537021.9.peg.297	organic solvent tolerance protein	Outer membrane protein Imp, required for envelope biogenesis / Organic solvent tolerance protein precursor	ABC-type exporter for lipopolysaccharide, auxiliary component	has signal peptide
**254780396**	537021.9.peg.299	peptidyl-prolyl cis-trans isomerase protein	Survival protein SurA precursor (Peptidyl-prolyl cis-trans isomerase SurA) (EC 5.2.1.8)	peptidyl-prolyl cis-trans isomerase protein (EC 5.2.1.8)	has signal peptide
**254780397**	537021.9.peg.300	4-hydroxythreonine-4-phosphate dehydrogenase	4-hydroxythreonine-4-phosphate dehydrogenase (EC 1.1.1.262)	4-hydroxythreonine-4-phosphate dehydrogenase (EC 1.1.1.262)	
**254780398**	537021.9.peg.301	dimethyladenosine transferase	Dimethyladenosine transferase (EC 2.1.1.-)	dimethyladenosine transferase (EC 2.1.1.-)	
**254780399**	537021.9.peg.302	DNA mismatch repair protein	DNA mismatch repair protein MutL	DNA mismatch repair protein	
**254780400**	537021.9.peg.303	hypothetical protein	hypothetical protein	unknown	
**254780401**	537021.9.peg.304	tetraacyldisaccharide 4'-kinase	Tetraacyldisaccharide 4’-kinase (EC 2.7.1.130)	tetraacyldisaccharide 4'-kinase (EC 2.7.1.130)	transmembrane protein
**254780402**	537021.9.peg.305	3-deoxy-D-manno-octulosonic-acid transferase	3-deoxy-D-manno-octulosonic-acid transferase (EC 2.-.-.-)	3-deoxy-D-manno-octulosonic-acid transferase (EC 2.-.-.-)	transmembrane protein
**254780403**	537021.9.peg.306	hypothetical protein	Uncharacterized protein, Bsl7517 homolog	unknown	
**254780404**	537021.9.peg.307	inositol monophosphatase family protein	FIG043197: Inositol monophosphatase family protein	inositol monophosphatase family protein	
**254780405**	537021.9.peg.308	hypothetical protein	hypothetical protein	N6-adenine-specific methylase (EC 2.1.1.-)	
**254780406**	537021.9.peg.309	putative ribosomal large subunit pseudouridine synthase B	Ribosomal large subunit pseudouridine synthase B (EC 4.2.1.70) LSU Psi2605	ribosomal large subunit pseudouridine synthase B (EC 4.2.1.70)	
**254780407**	537021.9.peg.310	hypothetical protein	hypothetical protein	unknown	
**254780408**	537021.9.peg.311	hypothetical protein	hypothetical protein	unknown	
**254780409**	537021.9.peg.312	ribose-phosphate pyrophosphokinase	Ribose-phosphate pyrophosphokinase (EC 2.7.6.1)	ribose-phosphate pyrophosphokinase (EC 2.7.6.1)	

**254780410**	537021.9.peg.313	hypothetical protein	COG1565: Uncharacterized conserved protein	unknown	
**254780411**	537021.9.peg.314	prolipoprotein diacylglyceryl transferase	Prolipoprotein diacylglyceryl transferase (EC 2.4.99.-)	prolipoprotein diacylglyceryl transferase (EC 2.4.99.-)	transmembrane protein
**254780412**	537021.9.peg.315	hypothetical protein	hypothetical protein	unknown	
**254780413**	537021.9.peg.316	PAS/PAC sensor signal transduction histidine kinase	Sensory box histidine kinase	Signal transduction histidine kinase	
**254780414**	537021.9.peg.317	GMP synthase	GMP synthase [glutamine-hydrolyzing] (EC 6.3.5.2)	GMP synthase (EC 6.3.5.2)	
**255764470**	537021.9.peg.318	integral membrane protein TerC	CBS domain protein	integral membrane protein TerC + TlyC, Hemolysins and related proteins containing CBS domains	transmembrane protein
**254780416**	537021.9.peg.319	putative ferredoxin protein	4Fe-4S ferredoxin, iron-sulfur binding	putative ferredoxin protein	
**254780417**	537021.9.peg.320	transcriptional regulator CarD family protein	CarD-like transcriptional regulator	transcriptional regulator CarD family protein	
**254780418**	537021.9.peg.321	50S ribosomal protein L25/general stress protein Ctc	LSU ribosomal protein L25p	50S ribosomal protein L25/general stress protei	
**254780419**	537021.9.peg.322	aspartyl-tRNA synthetase	Aspartyl-tRNA synthetase (EC 6.1.1.12), Aspartyl-tRNA(Asn) synthetase (EC 6.1.1.23) unambiguous	aspartyl-tRNA synthetase (EC 6.1.1.12)	
**254780420**	537021.9.peg.323	carbamoyl phosphate synthase small subunit	Carbamoyl-phosphate synthase small chain (EC 6.3.5.5)	carbamoyl phosphate synthase small subunit (EC 6.3.5.5)	
**254780421**	537021.9.peg.324	hypothetical protein	hypothetical protein	unknown	transmembrane protein
**255764471**	537021.9.peg.325	GTP-binding protein EngA	GTP-binding protein EngA	GTP-binding protein EngA	
**254780423**	537021.9.peg.326	S-adenosylmethionine synthetase	S-adenosylmethionine synthetase (EC 2.5.1.6)	S-adenosylmethionine synthetase (EC 2.5.1.6)	
**254780424**	537021.9.peg.327	transcriptional regulator protein	Predicted transcriptional regulator	transcriptional regulator protein	
**254780425**	537021.9.peg.328	apolipoprotein N-acyltransferase	Apolipoprotein N-acyltransferase (EC 2.3.1.-) / Copper homeostasis protein CutE	apolipoprotein N-acyltransferase (EC 2.3.1.-)	transmembrane protein
**254780426**	537021.9.peg.329	hemolysin protein	Magnesium and cobalt efflux protein CorC	hemolysin protein, transporter associated protein	
**254780427**	537021.9.peg.331	hypothetical protein	FIG000233: metal-dependent hydrolase	metal binding protein	
**255764472**	537021.9.peg.332	2-methylthioadenine synthetase (miaB-like) protein	tRNA-i(6)A37 methylthiotransferase	2-methylthioadenine synthetase	
**254780429**	537021.9.peg.333	hypothetical protein	Inactive homolog of metal-dependent proteases, putative molecular chaperone	metal-dependant protease	
**254780430**	537021.9.peg.334	nitrogen fixation protein	NifU protein	Nfu/NifU protein, may involve in formation of Fe-S cluster	
**254780431**	537021.9.peg.335	hypothetical protein	hypothetical protein	cell cycle regulator	
**254780432**	537021.9.peg.336	acetylornithine transaminase protein	Acetylornithine aminotransferase (EC 2.6.1.11)	acetylornithine transaminase protein (EC 2.6.1.11)	
**255764473**	537021.9.peg.337	ornithine carbamoyltransferase	Ornithine carbamoyltransferase (EC 2.1.3.3)	ornithine carbamoyltransferase (EC 2.1.3.3)	
**254780434**	537021.9.peg.338	dihydroorotate dehydrogenase 2	Dihydroorotate dehydrogenase (EC 1.3.3.1)	dihydroorotate dehydrogenase (EC 1.3.3.1)	
**254780435**	537021.9.peg.339	peptidase S11, D-alanyl-D-alanine carboxypeptidase 1	D-alanyl-D-alanine carboxypeptidase (EC 3.4.16.4)	D-alanyl-D-alanine carboxypeptidase (EC 3.4.16.4)	has signal peptide
**254780436**	537021.9.peg.340	hypothetical protein	hypothetical protein	unknown	transmembrane protein
**254780437**	537021.9.peg.341	30S ribosomal protein S21	SSU ribosomal protein S21p	30S ribosomal protein S21	should be mapped to ribosome pathway in KEGG… maybe the start point is wrong… if not wrong, rosetta may help predict the fix helix
**254780438**	537021.9.peg.342	pyridoxine 5'-phosphate synthase	Pyridoxine 5’-phosphate synthase (EC 2.6.99.2)	pyridoxine 5'-phosphate synthase (EC 2.6.99.2)	
**254780439**	537021.9.peg.343	carbamoyl phosphate synthase large subunit	Carbamoyl-phosphate synthase large chain (EC 6.3.5.5)	carbamoyl phosphate synthase large subunit (EC 6.3.5.5)	
**254780440**	537021.9.peg.344	transcription elongation factor GreA	Transcription elongation factor GreA	transcription elongation factor GreA	
**255764474**	537021.9.peg.345	glycosyl transferase group 1	Glycosyltransferase	glycosyl transferase	
**254780442**	537021.9.peg.346	pyruvate kinase	Pyruvate kinase (EC 2.7.1.40)	pyruvate kinase (EC 2.7.1.40)	
**254780443**	537021.9.peg.347	hypothetical protein	conserved hypothetical protein	unknown	has signal peptide
**254780444**	537021.9.peg.350	bacteriophage repressor protein C1	Phage repressor	transcriptional regulator, phage repressor	
**254780445**	537021.9.peg.351	isoleucyl-tRNA synthetase	Isoleucyl-tRNA synthetase (EC 6.1.1.5)	isoleucyl-tRNA synthetase (EC 6.1.1.5)	
**254780446**	537021.9.peg.352	hypothetical protein	hypothetical protein	unknown	
**254780448**	537021.9.peg.353	hypothetical protein	hypothetical protein	unknown	transmembrane protein
**254780449**	537021.9.peg.354	hypothetical protein	hypothetical protein	unknown	

**254780450**	537021.9.peg.355	two-component sensor histidine kinase/response regulator hybrid protein	Sensory box histidine kinase/response regulator	two-component sensor histidine kinase/respons	transmembrane protein
**254780451**	537021.9.peg.356	aconitate hydratase	Aconitate hydratase (EC 4.2.1.3)	aconitate hydratase (EC 4.2.1.3)	
**254780452**	537021.9.peg.357	hypothetical protein	FIG005935: membrane protein	unknown	transmembrane protein
**254780453**	537021.9.peg.358	orotidine 5'-phosphate decarboxylase	Orotidine 5’-phosphate decarboxylase (EC 4.1.1.23)	orotidine 5'-phosphate decarboxylase (EC 4.1.1.23)	
**254780454**	537021.9.peg.359	DNA polymerase III subunit beta	DNA polymerase III beta subunit (EC 2.7.7.7)	DNA polymerase III subunit beta (EC 2.7.7.7)	
**254780455**	537021.9.peg.361	nicotinate phosphoribosyltransferase	Nicotinate phosphoribosyltransferase (EC 2.4.2.11)	nicotinate phosphoribosyltransferase (EC 2.4.2.11)	
**254780456**	537021.9.peg.362	30S ribosomal protein S1	SSU ribosomal protein S1p	30S ribosomal protein S1	
**254780457**	537021.9.peg.364	cytidylate kinase	Cytidylate kinase (EC 2.7.4.14)	cytidylate kinase (EC 2.7.4.14)	
**254780458**	537021.9.peg.365	3-phosphoshikimate 1-carboxyvinyltransferase	5-Enolpyruvylshikimate-3-phosphate synthase (EC 2.5.1.19)	3-phosphoshikimate 1-carboxyvinyltransferase (EC 2.5.1.19)	
**254780459**	537021.9.peg.366	hypothetical protein	hypothetical protein	unknown	
**254780460**	537021.9.peg.367	3-hydroxydecanoyl-(acyl carrier protein) dehydratase	3-hydroxydecanoyl-[acyl-carrier-protein] dehydratase (EC 4.2.1.60)	3-hydroxydecanoyl-(acyl carrier protein) synthase (EC 2.3.1.41)	
**254780461**	537021.9.peg.368	3-oxoacyl-(acyl carrier protein) synthase I	3-oxoacyl-[acyl-carrier-protein] synthase, KASI (EC 2.3.1.41)	3-oxoacyl-(acyl carrier protein) synthase I (EC 2.3.1.41)	
**254780462**	537021.9.peg.369	enoyl-(acyl carrier protein) reductase	Enoyl-[acyl-carrier-protein] reductase [NADH] (EC 1.3.1.9)	enoyl-(acyl carrier protein) reductase (EC 1.3.1.9)	
**254780463**	537021.9.peg.370	putative peptidoglycan binding protein	putative hemagglutinin protein	unknown	transmembrane protein
**254780464**	537021.9.peg.371	M16 family peptidase	Mitochondrial processing peptidase-like protein (EC 3.4.24.64)	Zn-dependent peptidasese (EC 3.4.24.64)	
**254780465**	537021.9.peg.372	threonine synthase	Threonine synthase (EC 4.2.3.1)	threonine synthase (EC 4.2.3.1)	
**254780466**	537021.9.peg.373	putative modification methylase	DNA modification methyltransferase (EC 2.1.1.-)	DNA modification methylase (EC 2.1.1.-)	
**254780467**	537021.9.peg.375	exodeoxyribonuclease VII small subunit	Exodeoxyribonuclease VII small subunit (EC 3.1.11.6)	exodeoxyribonuclease VII small subunit (EC 3.1.11.6)	
**254780468**	537021.9.peg.376	sensory box/GGDEF family protein	Sensory box/GGDEF domain/EAL domain protein	signal transduction protein	transmembrane protein
**254780469**	537021.9.peg.377	zinc-binding protein	hypothetical protein	zinc-finger	
**254780470**	537021.9.peg.378	translation initiation factor IF-1	Translation initiation factor 1	translation initiation factor IF-1	
**254780471**	537021.9.peg.379	cytochrome o ubiquinol oxidase subunit IV	Cytochrome O ubiquinol oxidase subunit IV (EC 1.10.3.-)	cytochrome o ubiquinol oxidase subunit IV (EC 1.10.3.-)	transmembrane protein
**254780472**	537021.9.peg.380	cytochrome o ubiquinol oxidase subunit III	Cytochrome O ubiquinol oxidase subunit III (EC 1.10.3.-)	cytochrome o ubiquinol oxidase subunit III (EC 1.10.3.-)	transmembrane protein
**254780473**	537021.9.peg.381	cytochrome O ubiquinol oxidase subunit I	Cytochrome O ubiquinol oxidase subunit I (EC 1.10.3.-)	cytochrome O ubiquinol oxidase subunit I (EC 1.10.3.-)	transmembrane protein
**254780474**	537021.9.peg.382	ubiquinol oxidase, subunit II	Cytochrome O ubiquinol oxidase subunit II (EC 1.10.3.-)	ubiquinol oxidase, subunit II (EC 1.10.3.-)	transmembrane protein
**254780475**	537021.9.peg.383	50S ribosomal protein L33	LSU ribosomal protein L33p	50S ribosomal protein L33	
**254780476**	537021.9.peg.384	two component response regulator	PUTATIVE 2-COMPONENT RECEIVER DOMAIN PROTEIN	two component response regulator	
**254780477**	537021.9.peg.385	DSBA oxidoreductase	Periplasmic thiol:disulfide interchange protein DsbA	DSBA oxidoreductase	has signal peptide
**254780478**	537021.9.peg.386	hypothetical protein	Argininosuccinate synthase	unknown	
**254780479**	537021.9.peg.387	A/G-specific adenine glycosylase	A/G-specific adenine glycosylase (EC 3.2.2.-)	A/G-specific adenine glycosylase (EC 3.2.2.-)	
**254780480**	537021.9.peg.388	2'-deoxycytidine 5'-triphosphate deaminase	Deoxycytidine triphosphate deaminase (EC 3.5.4.13)	2'-deoxycytidine 5'-triphosphate deaminase (EC 3.5.4.13)	
**254780481**	537021.9.peg.389	50S ribosomal protein L34	hypothetical protein	50S ribosomal protein L34	
**254780482**	537021.9.peg.390	ribonuclease P	Ribonuclease P protein component (EC 3.1.26.5)	ribonuclease P (EC 3.1.26.5)	
**254780483**	537021.9.peg.391	putative inner membrane protein translocase component YidC	Inner membrane protein translocase component YidC, long form	inner membrane protein translocase component YidC	transmembrane protein
**254780484**	537021.9.peg.392	GTPase EngB	GTP-binding protein EngB	GTPase EngB	
**N/A**	537021.9.peg.393	N/A	Acetylglutamate kinase (EC 2.7.2.8)	Acetylglutamate kinase	1/2 of the protein
**N/A**	537021.9.peg.394	N/A	Acetylglutamate kinase (EC 2.7.2.8)	Acetylglutamate kinase	1/2 of the protein
**254780485**	537021.9.peg.395	biotin synthase	Biotin synthase (EC 2.8.1.6)	biotin synthase (EC 2.8.1.6)	
**254780486**	537021.9.peg.396	8-amino-7-oxononanoate synthase	8-amino-7-oxononanoate synthase (EC 2.3.1.47)	8-amino-7-oxononanoate synthase (EC 2.3.1.47)	
**254780487**	537021.9.peg.397	dithiobiotin synthetase	Dethiobiotin synthetase (EC 6.3.3.3)	dithiobiotin synthetase (EC 6.3.3.3)	
**254780488**	537021.9.peg.398	adenosylmethionine--8-amino-7-oxononanoate transaminase	Adenosylmethionine-8-amino-7-oxononanoate aminotransferase (EC 2.6.1.62)	Adenosylmethionine-8-amino-7-oxononanoate aminotransferase (EC 2.6.1.62)	

**254780489**	537021.9.peg.399	3-oxoacyl-(acyl carrier protein) synthase II	Biotin synthesis protein bioZ	3-oxoacyl-(acyl carrier protein) synthase II	
**254780490**	537021.9.peg.400	site-specific tyrosine recombinase XerC	probable integrase/recombinase protein	site-specific tyrosine recombinase XerC	
**254780491**	537021.9.peg.401	Mrp protein	Mrp protein	unknown	
**254780492**	N/A	hypothetical protein	N/A	unknown	
**254780829**	537021.9.peg.402	ATP-dependent protease ATP-binding subunit	ATP-dependent hsl protease ATP-binding subunit HslU	ATP-dependent protease ATP-binding subunit	
**254780828**	537021.9.peg.403	ATP-dependent protease peptidase subunit	ATP-dependent protease HslV (EC 3.4.25.-)	ATP-dependent protease peptidase subunit (EC 3.4.25.-)	
**254780827**	537021.9.peg.404	pantothenate kinase	Pantothenate kinase (EC 2.7.1.33)	pantothenate kinase (EC 2.7.1.33)	
**254780826**	537021.9.peg.405	phosphoenolpyruvate carboxykinase	Phosphoenolpyruvate carboxykinase [ATP] (EC 4.1.1.49)	phosphoenolpyruvate carboxykinase (EC 4.1.1.49)	
**255764476**	537021.9.peg.406	two-component sensor histidine kinase protein	FIG056333: sensor	two-component sensor histidine kinase protein	transmembrane protein
**254780824**	537021.9.peg.407	hypothetical protein	ATPase YjeE, predicted to have essential role in cell wall biosynthesis / COG3178: Predicted phosphotransferase related to Ser/Thr protein kinases	ATPase or kinase	
**254780823**	537021.9.peg.408	double-strand break repair protein AddB	FIG041266: ATP-dependent nuclease subunit B	double-strand break repair protein AddB	
**N/A**	537021.9.peg.409	N/A	FIG061771: ATP-dependent nuclease subunit A	double-strand break repair helicase AddA	1/2 of the protein
**N/A**	537021.9.peg.410	N/A	FIG061771: ATP-dependent nuclease subunit A	double-strand break repair helicase AddA	1/2 of the protein
**254780822**	537021.9.peg.411	thioredoxin	Thioredoxin	thioredoxin	
**254780821**	537021.9.peg.412	FolC bifunctional protein	Dihydrofolate synthase (EC 6.3.2.12) / Folylpolyglutamate synthase (EC 6.3.2.17)	FolC bifunctional protein	
**254780820**	537021.9.peg.413	acetyl-CoA carboxylase subunit beta	Acetyl-coenzyme A carboxyl transferase beta chain (EC 6.4.1.2)	acetyl-CoA carboxylase subunit beta (EC 6.4.1.2)	
**255764477**	537021.9.peg.415	dephospho-CoA kinase	Dephospho-CoA kinase (EC 2.7.1.24)	dephospho-CoA kinase (EC 2.7.1.24)	
**254780818**	537021.9.peg.416	DNA polymerase III subunit epsilon	DNA polymerase III epsilon subunit (EC 2.7.7.7)	DNA polymerase III subunit epsilon (EC 2.7.7.7)	
**254780817**	537021.9.peg.417	preprotein translocase subunit SecB	Protein export cytoplasm chaperone protein (SecB, maintains protein to be exported in unfolded state)	preprotein translocase subunit SecB	
**254780815**	537021.9.peg.418	hypothetical protein	hypothetical protein	membrane associated transporter protein	transmembrane protein
**254780814**	537021.9.peg.419	DNA gyrase subunit B	DNA gyrase subunit B (EC 5.99.1.3)	DNA gyrase subunit B (EC 5.99.1.3)	
**254780813**	537021.9.peg.420	Maf-like protein	Maf/YceF/YhdE family protein	Maf-like protein, cell cycle control	
**254780812**	537021.9.peg.421	uroporphyrinogen decarboxylase	Uroporphyrinogen III decarboxylase (EC 4.1.1.37)	uroporphyrinogen decarboxylase (EC 4.1.1.37)	
**254780811**	537021.9.peg.422	hypothetical protein	Hypothetical membrane protein, possible involvement in cytochrome functioning/assembly	unknown	transmembrane protein
**254780810**	537021.9.peg.424	transcription termination factor Rho	Transcription termination factor Rho	transcription termination factor Rho	
**254780809**	537021.9.peg.425	tRNA modification GTPase TrmE	GTPase and tRNA-U34 5-formylation enzyme TrmE	tRNA modification GTPase TrmE	
**254780808**	537021.9.peg.426	tRNA uridine 5-carboxymethylaminomethyl modification enzyme GidA	tRNA uridine 5-carboxymethylaminomethyl modification enzyme GidA	tRNA uridine 5-carboxymethylaminomethy modification enzyme GidA	
**254780807**	537021.9.peg.427	glucose-inhibited division protein B	probable glucose inhibited division protein B	glucose-inhibited division protein B	
**254780806**	537021.9.peg.428	chromosome partitioning protein A	Chromosome (plasmid) partitioning protein ParA / Sporulation initiation inhibitor protein Soj	chromosome partitioning protein A	
**254780805**	537021.9.peg.429	chromosome partitioning protein B	Chromosome (plasmid) partitioning protein ParB / Stage 0 sporulation protein J	chromosome partitioning protein B	
**254780804**	537021.9.peg.430	DNA polymerase III subunit delta	DNA polymerase III delta subunit (EC 2.7.7.7)	DNA polymerase III subunit delta (EC 2.7.7.7)	
**254780803**	537021.9.peg.431	hypothetical protein	hypothetical protein	unknown	transmembrane protein
**254780802**	537021.9.peg.432	leucyl-tRNA synthetase	Leucyl-tRNA synthetase (EC 6.1.1.4)	leucyl-tRNA synthetase (EC 6.1.1.4)	
**254780801**	537021.9.peg.433	hypothetical protein	Hypothetical protein YggS, proline synthase co-transcribed bacterial homolog PROSC	enzyme of TIM barrel fold	
**254780800**	537021.9.peg.434	hypothetical protein	hypothetical protein	unknown	
**254780799**	537021.9.peg.435	DNA translocase FtsK	Cell division protein FtsK	DNA translocase FtsK	transmembrane protein
**254780798**	537021.9.peg.436	possible lolA type protein	outer membrane lipoprotein carrier protein LolA	ABC transporter for lipoprotein, auxiliary component	has signal peptide
**254780797**	537021.9.peg.437	hypothetical protein	hypothetical protein	Exonuclease III	
**254780796**	537021.9.peg.438	ribonuclease H	Ribonuclease HI (EC 3.1.26.4)	ribonuclease H (EC 3.1.26.4)	
**254780795**	537021.9.peg.439	homoserine kinase	Homoserine kinase (EC 2.7.1.39)	homoserine kinase (EC 2.7.1.39)	
**254780794**	537021.9.peg.440	tryptophanyl-tRNA synthetase	Tryptophanyl-tRNA synthetase (EC 6.1.1.2)	tryptophanyl-tRNA synthetase (EC 6.1.1.2)	
**254780793**	537021.9.peg.441	integral membrane protein MviN	hypothetical protein	integral membrane protein MviN	transmembrane protein
**255764478**	537021.9.peg.442	aminopeptidase protein	Aminopeptidase S (Leu, Val, Phe, Tyr preference) (EC	aminopeptidase protein (EC 3.4.11.24)	

			3.4.11.24)		
**254780791**	537021.9.peg.443	exodeoxyribonuclease VII large subunit	Exodeoxyribonuclease VII large subunit (EC 3.1.11.6)	exodeoxyribonuclease VII large subunit	
**254780790**	N/A	hypothetical protein	N/A	unknown	transmembrane protein
**254780789**	537021.9.peg.444	hypothetical protein	COG0779: clustered with transcription termination protein NusA	unknown	
**254780788**	537021.9.peg.445	transcription elongation factor NusA	Transcription termination protein NusA	transcription elongation/termination factor NusA	
**254780787**	537021.9.peg.446	translation initiation factor IF-2	Translation initiation factor 2	translation initiation factor IF-2	
**254780786**	537021.9.peg.447	ribosome-binding factor A	Ribosome-binding factor A	ribosome-binding factor A	
**254780785**	537021.9.peg.448	30S ribosomal protein S15	SSU ribosomal protein S15p (S13e)	30S ribosomal protein S15	
**254780784**	537021.9.peg.449	polynucleotide phosphorylase/polyadenylase	Polyribonucleotide nucleotidyltransferase (EC 2.7.7.8)	polynucleotide phosphorylase/polyadenylase (EC 2.7.7.8)	
**254780783**	537021.9.peg.450	transcription regulator protein	TRANSCRIPTION REGULATOR PROTEIN	ROS/MUCR transcription regulator	
**255764479**	537021.9.peg.452	hypothetical protein	hypothetical protein	unknown	
**254780782**	537021.9.peg.451	succinyl-diaminopimelate desuccinylase	N-succinyl-L,L-diaminopimelate desuccinylase (EC 3.5.1.18)	succinyl-diaminopimelate desuccinylase (EC 3.5.1.18)	
**254780781**	537021.9.peg.453	2,3,4,5-tetrahydropyridine-2,6-carboxylate N-succinyltransferase	2,3,4,5-tetrahydropyridine-2,6-dicarboxylate N-succinyltransferase (EC 2.3.1.117)	2,3,4,5-tetrahydropyridine-2,6-carboxylate N-succinyltransferase (EC 2.3.1.117)	
**254780780**	537021.9.peg.454	histidine triad (HIT) protein	histidine triad (HIT) protein	HIT family hydrolyase involve in cell cycle	
**254780779**	537021.9.peg.456	30S ribosomal protein S2	SSU ribosomal protein S2p (SAe)	30S ribosomal protein S2	
**254780778**	537021.9.peg.457	elongation factor Ts	Translation elongation factor Ts	elongation factor Ts	
**254780777**	537021.9.peg.458	uridylate kinase	Uridylate kinase (EC 2.7.4.-)	uridylate kinase (EC 2.7.4.-)	
**254780776**	537021.9.peg.459	ribosome recycling factor	Ribosome recycling factor	ribosome recycling factor	
**254780775**	537021.9.peg.460	undecaprenyl diphosphate synthase	Undecaprenyl pyrophosphate synthetase (EC 2.5.1.31)	undecaprenyl diphosphate synthase (EC 2.5.1.31)	
**254780774**	537021.9.peg.461	phosphatidate cytidylyltransferase protein	Phosphatidate cytidylyltransferase (EC 2.7.7.41)	phosphatidate cytidylyltransferase protein (EC 2.7.7.41)	transmembrane protein
**254780773**	537021.9.peg.462	zinc metallopeptidase	Membrane-associated zinc metalloprotease	membrane-associated Zn-dependent protease	transmembrane protein
**254780772**	537021.9.peg.463	surface antigen (D15)	Outer membrane protein assembly factor YaeT precursor	YaeT in YaeT/YfiO complex for assembling proteins into outer membrane	has signal peptide
**254780771**	537021.9.peg.464	UDP-3-O-[3-hydroxymyristoyl] glucosamine N-acyltransferase	UDP-3-O-[3-hydroxymyristoyl] glucosamine N-acyltransferase (EC 2.3.1.-)	UDP-3-O-[3-hydroxymyristoyl] glucosamine N-acyltransferase (EC 2.3.1.-)	
**254780770**	537021.9.peg.465	(3R)-hydroxymyristoyl-ACP dehydratase	(3R)-hydroxymyristoyl-[acyl carrier protein] dehydratase (EC 4.2.1.-)	(3R)-hydroxymyristoyl-ACP dehydratase (EC 4.2.1.-)	
**255764481**	537021.9.peg.466	UDP-N-acetylglucosamine acyltransferase	Acyl-[acyl-carrier-protein]--UDP-N-acetylglucosamine O-acyltransferase (EC 2.3.1.129)	UDP-N-acetylglucosamine acyltransferase (EC 2.3.1.129)	
**254780768**	537021.9.peg.467	hypothetical protein	Protein of unknown function DUF1009 clustered with KDO2-Lipid A biosynthesis genes	unknown	
**254780767**	537021.9.peg.468	lipid-A-disaccharide synthase	Lipid-A-disaccharide synthase (EC 2.4.1.182)	lipid-A-disaccharide synthase (EC 2.4.1.182)	
**254780766**	537021.9.peg.469	recombination protein F	DNA recombination and repair protein RecF	DNA recombination protein F, RecF	
**254780765**	537021.9.peg.470	3-deoxy-manno-octulosonate cytidylyltransferase	3-deoxy-manno-octulosonate cytidylyltransferase (EC 2.7.7.38)	3-deoxy-manno-octulosonate cytidylyltransferase (EC 2.7.7.38)	
**254780764**	537021.9.peg.471	hypothetical protein	hypothetical protein	signal transduction protein	has signal peptide
**N/A**	537021.9.peg.472	N/A	DNA polymerase III subunits gamma and tau (EC 2.7.7.7)	DNA polymerase III subunits gamma and tau	1/2 of the protein
**N/A**	537021.9.peg.473	N/A	hypothetical protein	DNA polymerase III subunits gamma and tau	1/2 of the protein
**254780763**	537021.9.peg.474	hypothetical protein	FIG000557: hypothetical protein	unknown	
**254780762**	537021.9.peg.475	recombination protein RecR	Recombination protein RecR	recombination protein RecR	
**N/A**	537021.9.peg.476	N/A	virulence factor MviN-like protein	integral membrane protein MviN	transmembrane protein, 1/2 of the protein
**N/A**	537021.9.peg.477	N/A	virulence factor MviN homolog	integral membrane protein MviN	transmembrane protein, 1/2 of the protein
**254780761**	537021.9.peg.478	probable FAD-dependent oxidoreductase protein	D-2-hydroxyglutarate dehydrogenase	glycolate oxidase subunit GlcD	
**254780760**	537021.9.peg.479	hypothetical protein	YrdC/Sua5 family protein, required for threonylcarbamoyladenosine (t(6)A) formation in tRNA	translational regulation protein	
**254780759**	537021.9.peg.480	hypothetical protein	hypothetical protein	unknown	has signal peptide
**254780758**	537021.9.peg.481	hypothetical protein	Hypothetical metal-binding enzyme, YcbL homolog	zn-depedent hydrolase	

**254780757**	537021.9.peg.482	glycyl-tRNA synthetase subunit alpha	Glycyl-tRNA synthetase alpha chain (EC 6.1.1.14)	glycyl-tRNA synthetase subunit alpha (EC 6.1.1.14)	
**255764482**	537021.9.peg.483	methyltransferase protein	COG4123: Predicted O-methyltransferase	O-methyltransferase	
**254780755**	537021.9.peg.484	octaprenyl-diphosphate synthase protein	Octaprenyl-diphosphate synthase (EC 2.5.1.-) / Dimethylallyltransferase (EC 2.5.1.1) / Geranyltranstransferase (farnesyldiphosphate synthase) (EC 2.5.1.10) / Geranylgeranyl pyrophosphate synthetase (EC 2.5.1.29)	octaprenyl-diphosphate synthase protein (EC 2.5.1.-)	
**254780754**	537021.9.peg.485	inorganic polyphosphate/ATP-NAD kinase	NAD kinase (EC 2.7.1.23)	inorganic polyphosphate/ATP-NAD kinase (EC 2.7.1.23)	
**254780753**	537021.9.peg.486	peptide chain release factor 2	Peptide chain release factor 2; programmed frameshift-containing	peptide chain release factor 2	
**254780752**	537021.9.peg.487	penicillin binding peptidoglycan synthetase protein	Multimodular transpeptidase-transglycosylase (EC 2.4.1.129) (EC 3.4.-.-)	penicillin binding peptidoglycan synthetase (EC 2.4.1.-) (EC 3.4.-.-)	transmembrane protein
**254780751**	537021.9.peg.488	ribonuclease E	Cytoplasmic axial filament protein CafA and Ribonuclease G (EC 3.1.4.-)	ribonuclease E	
**254780750**	537021.9.peg.489	DNA mismatch repair protein	DNA mismatch repair protein MutS	DNA mismatch repair protein, MutS	
**254780749**	537021.9.peg.490	lipoprotein signal peptidase transmembrane	Lipoprotein signal peptidase (EC 3.4.23.36)	Lipoprotein signal peptidase (EC 3.4.23.36)	transmembrane protein
**254780748**	537021.9.peg.491	integration host factor, beta subunit	Integration host factor beta subunit	integration host factor, beta subunit	
**254780747**	537021.9.peg.492	putative protease IV transmembrane protein	macromolecule metabolism; macromolecule degradation; degradation of proteins, peptides, glycopeptides	protease IV transmembrane protein	has signal peptide
**254780746**	537021.9.peg.494	hypothetical protein	hypothetical protein	outer membrane protein, involve in lipopolysaccharide export	transmembrane protein
**254780745**	537021.9.peg.495	OstA family protein	hypothetical protein	outer membrane protein, involve in lipopolysaccharide export	has signal peptide
**254780744**	537021.9.peg.496	ABC transporter nucleotide binding/ATPase protein	Lipopolysaccharide ABC transporter, ATP-binding protein LptB	ABC-type exporter for lipopolysaccharide, ATPase component	
**254780743**	537021.9.peg.497	putative uracil-DNA glycosylase	Uracil-DNA glycosylase, family 4	Uracil-DNA glycosylase	
**254780742**	537021.9.peg.498	hypothetical protein	Carboxynorspermidine dehydrogenase, putative (EC 1.1.1.-)	Saccharopine dehydrogenase and related protein	
**254780741**	537021.9.peg.499	carboxynorspermidine decarboxylase	Carboxynorspermidine decarboxylase, putative (EC 4.1.1.-)	carboxynorspermidine decarboxylase (EC 4.1.1.-)	
**254780740**	537021.9.peg.500	peptidase S16 lon domain protein	Uncharacterized protein, similar to the N-terminal domain of Lon protease	ATP-dependent protease, La	
**254780739**	537021.9.peg.501	hypothetical protein	protein of unknown function DUF343	unknown	
**255764483**	537021.9.peg.502	hypothetical protein	hypothetical protein	unknown	
**254780737**	537021.9.peg.503	hypothetical protein	hypothetical protein	unknown	has signal peptide
**254780736**	537021.9.peg.504	hypothetical protein	Flp pilus assembly protein, pilin Flp	Flp pilus assembly protein, flp	transmembrane protein, but might be processed by cpaA and become periplasmic
**254780735**	537021.9.peg.505	hypothetical protein	hypothetical protein	Flp pilus assembly protein,flp	has signal peptide
**254780734**	537021.9.peg.506	Flp/Fap pilin component	hypothetical protein	Flp pilus assembly protein, flp	transmembrane protein, but might be processed by cpaA and become periplasmic
**254780733**	537021.9.peg.507	hypothetical protein	hypothetical protein	Flp pilus assembly protein, flp	transmembrane protein, but might be processed by cpaA and become periplasmic
**254780732**	537021.9.peg.508	hypothetical protein	Flp pilus assembly protein, pilin Flp	Flp pilus assembly protein, flp	transmembrane protein, but might be processed by cpaA and become periplasmic
**254780730**	537021.9.peg.510	hypothetical protein	Flp pilus assembly protein, pilin Flp	Flp pilus assembly protein, flp	transmembrane protein, but might be processed by cpaA and become

					periplasmic
**254780729**	537021.9.peg.511	peptidase A24A prepilin type IV	Type IV prepilin peptidase TadV/CpaA	Flp pilus assembly protein CpaA	transmembrane protein
**254780728**	537021.9.peg.512	pilus assembly protein	Flp pilus assembly protein RcpC/CpaB	Flp pilus assembly protein CpaB	has signal peptide
**254780727**	537021.9.peg.513	putative pilus assembly protein	Type II/IV secretion system secretin RcpA/CpaC, associated with Flp pilus assembly	Flp pilus assembly protein, secretin CpaC	has signal peptide
**255764485**	537021.9.peg.514	hypothetical protein	Flp pilus assembly protein CpaD	Flp pilus assembly protein, ATPase CpaD	has signal peptide
**254780725**	537021.9.peg.515	response regulator receiver protein	Type II/IV secretion system ATPase TadZ/CpaE, associated with Flp pilus assembly	Flp pilus assembly protein, ATPase CpaE	
**254780724**	537021.9.peg.516	component of type IV pilus	Type II/IV secretion system ATP hydrolase TadA/VirB11/CpaF, TadA subfamily	Flp pilus assembly protein, ATPase CpaF	
**254780723**	537021.9.peg.517	hypothetical protein	hypothetical protein	Flp pilus assembly protein TadB	transmembrane protein
**254780722**	537021.9.peg.518	pilus assembly protein	Flp pilus assembly protein TadB	Flp pilus assembly protein TadB	transmembrane protein, 1/2 of the protein
**254780721**	537021.9.peg.519	pilus component protein	Type II/IV secretion system protein TadC, associated with Flp pilus assembly	Flp pilus assembly protein TadC	transmembrane protein
**254780720**	537021.9.peg.520	Ferroxidase	Ferritin-like protein 2	Ferroxidase	
**254780719**	537021.9.peg.521	zinc uptake ABC transporter, permease protein	Zinc ABC transporter, inner membrane permease protein ZnuB	ABC-type importer for zinc, transmembrane component	transmembrane protein
**254780718**	537021.9.peg.522	putative high-affinity zinc uptake system ATP-binding component of ABC transporter protein	Zinc ABC transporter, ATP-binding protein ZnuC	ABC-type importer for zinc, ATPase component	
**254780717**	537021.9.peg.524	zinc uptake ABC transporter	Zinc ABC transporter, periplasmic-binding protein ZnuA	ABC-type importer for zinc, substrate binding component	has signal peptide
**254780716**	537021.9.peg.525	6-phosphogluconate dehydrogenase	6-phosphogluconate dehydrogenase, decarboxylating (EC 1.1.1.44)	6-phosphogluconate dehydrogenase (EC 1.1.1.44)	
**254780715**	537021.9.peg.526	50S ribosomal protein L19	LSU ribosomal protein L19p	50S ribosomal protein L19	
**254780714**	537021.9.peg.527	tRNA (guanine-N(1)-)-methyltransferase	tRNA (Guanine37-N1) -methyltransferase (EC 2.1.1.31)	tRNA-(guanine-N1)-methyltransferase, trmD (EC 2.1.1.31)	
**254780713**	537021.9.peg.528	16S rRNA-processing protein	16S rRNA processing protein RimM	rimM, 16S rRNA-processing protein	
**254780712**	537021.9.peg.529	30S ribosomal protein S16	SSU ribosomal protein S16p	30S ribosomal protein S16	
**254780711**	537021.9.peg.530	signal recognition particle protein	Signal recognition particle, subunit Ffh SRP54 (TC 3.A.5.1.1)	signal recognition particle protein	
**254780710**	537021.9.peg.531	diaminopimelate epimerase	Diaminopimelate epimerase (EC 5.1.1.7)	diaminopimelate epimerase (EC 5.1.1.7)	
**254780709**	537021.9.peg.533	cell division protein	Signal recognition particle receptor protein FtsY (=alpha subunit) (TC 3.A.5.1.1)	cell division protein FtsY	
**254780708**	537021.9.peg.534	intracellular septation protein A	hypothetical protein	intracellular septation protein A	transmembrane protein
**254780707**	537021.9.peg.535	putative phosphate-binding periplasmic protein	Phosphate ABC transporter, periplasmic phosphate-binding protein PstS (TC 3.A.1.7.1)	ABC-type importer for phosphate, substrate binding component	has signal peptide
**255764486**	537021.9.peg.536	ABC transporter, membrane spanning protein	Phosphate transport system permease protein PstC (TC 3.A.1.7.1)	ABC-type importer for phosphate, transmembrane component	transmembrane protein
**254780705**	537021.9.peg.537	phosphate ABC transporter, permease protein PstA	Phosphate transport system permease protein PstA (TC 3.A.1.7.1)	ABC-type importer for phosphate, transmembrane component	transmembrane protein
**254780704**	537021.9.peg.538	ABC transporter, nucleotide binding/ATPase protein	Phosphate transport ATP-binding protein PstB (TC 3.A.1.7.1)	ABC-type importer for phosphate, ATPase component	
**254780703**	537021.9.peg.539	putative phosphate transport system protein	Phosphate transport system regulatory protein PhoU	phosphate transport system regulatory protein	
**255764487**	537021.9.peg.540	heat shock protein	Heat shock protein GrpE	heat shock protein	
**254780701**	537021.9.peg.541	putative two-component sensor histidine kinase transcriptional regulatory protein	Sensor histidine kinase (EC 2.7.3.-)	two-component sensor histidine kinase (EC 2.7.3.-)	transmembrane protein
**254780700**	537021.9.peg.543	serine protease DO-like protease	putative serine protease	serine protease DO-like protease	has signal peptide
**254780699**	537021.9.peg.544	hypothetical protein	hypothetical protein	unknown	
**254780698**	537021.9.peg.545	D-alanyl-D-alanine carboxypeptidase 1 penicillin-binding protein	D-alanyl-D-alanine carboxypeptidase (EC 3.4.16.4)	D-alanyl-D-alanine carboxypeptidase (EC 3.4.16.4)	has signal peptide
**254780697**	537021.9.peg.546	large conductance mechanosensitive channel protein	Large-conductance mechanosensitive channel	large conductance mechanosensitive channel	transmembrane protein
**254780696**	537021.9.peg.547	glutathione synthetase	Glutathione synthetase (EC 6.3.2.3)	glutathione synthetase (EC 6.3.2.3)	
**254780695**	537021.9.peg.548	flagellar MS-ring protein	Flagellar M-ring protein FliF	flagellar MS-ring protein	transmembrane protein
**254780694**	537021.9.peg.549	probable transcriptional regulator protein, LuxR	transcriptional regulator	transcriptional regulator	

		family			
**254780693**	537021.9.peg.550	transcriptional regulator protein	PUTATIVE TRANSCRIPTION REGULATOR PROTEIN	transcriptional regulator	
**254780691**	537021.9.peg.551	hypothetical protein	hypothetical protein	unknown	
**254780690**	537021.9.peg.552	flagellar biosynthesis protein FlhB	Flagellar biosynthesis protein FlhB	flagellar biosynthesis protein FlhB	transmembrane protein
**254780689**	537021.9.peg.553	flagellar motor switch protein G	Flagellar motor switch protein FliG	flagellar motor switch protein G	
**254780688**	537021.9.peg.554	putative flagellar motor switch protein	putative flagellar motor switch protein	putative flagellar motor switch protein	
**254780687**	537021.9.peg.555	flagellar C-ring protein	Flagellar motor switch protein FliM	flagellar C-ring protein	
**255764488**	537021.9.peg.556	flagellar motor protein MotA	Flagellar motor rotation protein MotA	flagellar motor protein MotA	transmembrane protein
**254780685**	537021.9.peg.557	flagellar basal body rod protein FlgF	Flagellar basal-body rod protein flgF	flagellar basal body rod protein FlgF	outermembrane, transported by flagellar assembly machinery
**254780684**	537021.9.peg.558	flagellum-specific ATP synthase	Flagellum-specific ATP synthase FliI	flagellum-specific ATP synthase	
**254780683**	537021.9.peg.559	lipoprotein	lipoprotein precursor protein	Peptidase M23	
**254780682**	537021.9.peg.560	hypothetical protein	hypothetical protein	Peptidase M23	
**255764489**	537021.9.peg.561	stationary phase survival protein SurE	5-nucleotidase SurE (EC 3.1.3.5)	stationary phase survival protein SurE (EC 3.1.3.5)	
**254780680**	537021.9.peg.562	seryl-tRNA synthetase	Seryl-tRNA synthetase (EC 6.1.1.11)	seryl-tRNA synthetase (EC 6.1.1.11)	
**254780679**	N/A	hypothetical protein	N/A	unknown	transmembrane protein
**254780677**	537021.9.peg.564	hypothetical protein	Putative oligoketide cyclase/lipid transport protein	Oligoketide cyclase/lipid transport protein	
**254780676**	537021.9.peg.565	lipoyl synthase	Lipoate synthase	lipoyl synthase	
**254780675**	537021.9.peg.566	dihydrolipoamide dehydrogenase	Dihydrolipoamide dehydrogenase of pyruvate dehydrogenase complex (EC 1.8.1.4)	Dihydrolipoamide dehydrogenase of pyruvate dehydrogenase complex (EC 1.8.1.4)	
**254780674**	537021.9.peg.567	pyruvate dehydrogenase complex dihydrolipoamide acetyltransferase	Dihydrolipoamide acetyltransferase component of pyruvate dehydrogenase complex (EC 2.3.1.12)	pyruvate dehydrogenase complex dihydrolipoamide acetyltransferase (EC 2.3.1.12)	
**254780673**	537021.9.peg.569	pyruvate dehydrogenase subunit beta	Pyruvate dehydrogenase E1 component beta subunit (EC 1.2.4.1)	pyruvate dehydrogenase subunit beta (EC 1.2.4.1)	
**255764490**	537021.9.peg.570	dehydrogenase, E1 component	Pyruvate dehydrogenase E1 component alpha subunit (EC 1.2.4.1)	dehydrogenase, E1 component (EC 1.2.4.1)	
**254780671**	537021.9.peg.571	putative cell division protein	Septum formation initiator	cell division protein	transmembrane protein
**254780670**	537021.9.peg.572	phosphopyruvate hydratase	Enolase (EC 4.2.1.11)	phosphopyruvate hydratase (EC 4.2.1.11)	
**254780669**	537021.9.peg.573	2-dehydro-3-deoxyphosphooctonate aldolase	2-Keto-3-deoxy-D-manno-octulosonate-8-phosphate synthase (EC 2.5.1.55)	2-dehydro-3-deoxyphosphooctonate aldolase (EC 2.5.1.55)	
**254780668**	537021.9.peg.575	hypothetical protein	hypothetical protein	unknown	
**254780666**	537021.9.peg.576	Fmu (Sun) domain protein	16S rRNA m(5)C 967 methyltransferase (EC 2.1.1.-)	tRNA and rRNA cytosine-C5-methylasese (EC 2.1.1.-)	
**254780664**	537021.9.peg.578	bifunctional phosphoribosylaminoimidazolecarboxamide formyltransferase/IMP cyclohydrolase	IMP cyclohydrolase (EC 3.5.4.10) / Phosphoribosylaminoimidazolecarboxamide formyltransferase (EC 2.1.2.3)	bifunctional phosphoribosylaminoimidazolecarboxamide formyltransferase/IMP cyclohydrolase (EC 1.4.1.2) (EC 1.4.1.2)	
**254780663**	537021.9.peg.579	carbonate dehydratase	Carbonic anhydrase (EC 4.2.1.1)	carbonate dehydratase (EC 4.2.1.1)	
**254780662**	537021.9.peg.580	NAD-glutamate dehydrogenase	NAD-specific glutamate dehydrogenase (EC 1.4.1.2), large form	NAD-glutamate dehydrogenase (EC 1.4.1.2)	
**254780661**	537021.9.peg.581	exonuclease I	Exodeoxyribonuclease I (EC 3.1.11.1)	exonuclease I (EC 3.1.11.1)	
**254780660**	537021.9.peg.583	hypothetical protein	FIG000859: hypothetical protein	unknown	
**254780659**	537021.9.peg.584	hypothetical protein	FIG006542: Phosphoesterase	Calcineurin-like phosphoesterase	
**254780658**	537021.9.peg.585	5-formyltetrahydrofolate cyclo-ligase	5-formyltetrahydrofolate cyclo-ligase (EC 6.3.3.2)	5-formyltetrahydrofolate cyclo-ligase (EC 6.3.3.2)	1/2 of the protein
**254780657**	537021.9.peg.586	hypothetical protein	hypothetical protein	5-formyltetrahydrofolate cyclo-ligase (EC 6.3.3.2)	1/2 of the protein
**255764492**	537021.9.peg.587	hypothetical protein	hypothetical protein	unknown	
**254780655**	537021.9.peg.588	transketolase	Transketolase (EC 2.2.1.1)	transketolase (EC 2.2.1.1)	
**254780654**	537021.9.peg.589	Glyceraldehyde 3-Phosphate Dehydrogenase	NAD-dependent glyceraldehyde-3-phosphate dehydrogenase (EC 1.2.1.12)	Glyceraldehyde 3-Phosphate Dehydrogenase (EC 1.2.1.12)	
**254780653**	537021.9.peg.590	phosphoglycerate kinase	Phosphoglycerate kinase (EC 2.7.2.3)	phosphoglycerate kinase (EC 2.7.2.3)	
**254780652**	537021.9.peg.591	Fructose-bisphosphate aldolase	Fructose-bisphosphate aldolase class I (EC 4.1.2.13)	Fructose-bisphosphate aldolase (EC 4.1.2.13)	
**254780650**	537021.9.peg.592	hypothetical protein	hypothetical protein	unknown	
**254780649**	537021.9.peg.594	nicotinic acid mononucleotide adenylyltransferase	Nicotinate-nucleotide adenylyltransferase (EC 2.7.7.18) bacterial NadD family	nicotinic acid mononucleotide adenylyltransferase (EC 2.7.7.18)	

**254780648**	537021.9.peg.595	GTPase ObgE	COG0536: GTP-binding protein Obg	GTPase ObgE, regulator of replication	
**254780647**	537021.9.peg.596	50S ribosomal protein L27	LSU ribosomal protein L27p	50S ribosomal protein L27	
**254780646**	537021.9.peg.597	50S ribosomal protein L21	LSU ribosomal protein L21p	50S ribosomal protein L21	
**254780645**	537021.9.peg.599	hypothetical protein	hypothetical protein	unknown	
**254780644**	537021.9.peg.600	hypothetical protein	hypothetical protein	unknown	
**254780642**	537021.9.peg.601	hypothetical protein	hypothetical protein	unknown	
**254780641**	537021.9.peg.602	hypothetical protein	hypothetical protein	Chromosome segregation ATPases	1/2 of the protein
**254780640**	N/A	hypothetical protein	N/A	Chromosome segregation ATPases	1/2 of the protein
**254780639**	537021.9.peg.604	hypothetical protein	hypothetical protein	unknown	
**254780638**	537021.9.peg.605	molecular chaperone protein DnaJ	Chaperone protein DnaJ	molecular chaperone protein DnaJ	
**255764494**	537021.9.peg.606	molecular chaperone DnaK	Chaperone protein DnaK	molecular chaperone DnaK	
**254780636**	537021.9.peg.607	ribonuclease D	Ribonuclease D related protein	ribonuclease D	
**254780635**	537021.9.peg.608	hypothetical protein	Iron-regulated protein A precursor	Uncharacterized iron-regulated protein	has signal peptide
**254780634**	537021.9.peg.609	NOL1/NOP2/SUN family signature protein	Sun protein	tRNA and rRNA cytosine-C5-methylasese	
**254780633**	537021.9.peg.610	creatinine amidohydrolase	Creatinine amidohydrolase (EC 3.5.2.10)	creatinine amidohydrolase (EC 3.5.2.10)	
**254780632**	537021.9.peg.611	hypothetical protein	hypothetical protein	unknown	
**254780630**	537021.9.peg.613	ribonuclease PH	Ribonuclease PH (EC 2.7.7.56)	ribonuclease PH (EC 2.7.7.56)	
**254780629**	537021.9.peg.614	putative deoxyribonucleotide triphosphate pyrophosphatase	Nucleoside 5-triphosphatase RdgB (dHAPTP, dITP, XTP-specific) (EC 3.6.1.15)	putative deoxyribonucleotide triphosphate pyrophosphatase (EC 3.6.1.15)	
**254780628**	537021.9.peg.615	coproporphyrinogen III oxidase	Radical SAM family enzyme, similar to coproporphyrinogen III oxidase, oxygen-independent, clustered with nucleoside-triphosphatase RdgB	coproporphyrinogen III oxidase	
**254780627**	537021.9.peg.616	chromosomal replication initiation protein	Chromosomal replication initiator protein DnaA	chromosomal replication initiation protein	
**254780626**	537021.9.peg.617	30S ribosomal protein S20	SSU ribosomal protein S20p	30S ribosomal protein S20	
**254780625**	537021.9.peg.618	formamidopyrimidine-DNA glycosylase	Formamidopyrimidine-DNA glycosylase (EC 3.2.2.23)	formamidopyrimidine-DNA glycosylase (EC 3.2.2.23)	
**254780624**	537021.9.peg.619	ubiquinone/menaquinone biosynthesis methyltransferase	Ubiquinone/menaquinone biosynthesis methyltransferase UbiE (EC 2.1.1.-)	ubiquinone/menaquinone biosynthesis methyltransferase (EC 2.1.1.-)	
**254780623**	537021.9.peg.620	2-polyprenylphenol 6-hydroxylase	Ubiquinone biosynthesis monooxygenase UbiB	2-polyprenylphenol 6-hydroxylase	transmembrane protein
**254780622**	537021.9.peg.621	bifunctional phosphopantothenoylcysteine decarboxylase/phosphopantothenate synthase	Phosphopantothenoylcysteine decarboxylase (EC 4.1.1.36) / Phosphopantothenoylcysteine synthetase (EC 6.3.2.5)	Phosphopantothenoylcysteine decarboxylase (EC 4.1.1.36) / Phosphopantothenoylcysteine synthetase (EC 6.3.2.5)	
**254780621**	537021.9.peg.622	small heat shock protein	16 kDa heat shock protein A	small heat shock protein	
**254780620**	537021.9.peg.623	iron-responsive transcriptional regulator	Iron-responsive repressor RirA	iron-responsive transcriptional regulator	
**254780619**	537021.9.peg.624	primosome assembly protein PriA	Helicase PriA essential for oriC/DnaA-independent DNA replication	primosome assembly protein PriA	
**254780618**	537021.9.peg.625	F0F1 ATP synthase subunit delta	ATP synthase delta chain (EC 3.6.3.14)	F0F1 ATP synthase subunit delta (EC 3.6.3.14)	
**254780617**	537021.9.peg.626	F0F1 ATP synthase subunit alpha	ATP synthase alpha chain (EC 3.6.3.14)	F0F1 ATP synthase subunit alpha (EC 3.6.3.14)	
**254780616**	537021.9.peg.627	F0F1 ATP synthase subunit gamma	ATP synthase gamma chain (EC 3.6.3.14)	F0F1 ATP synthase subunit gamma (EC 3.6.3.14)	
**254780615**	537021.9.peg.628	F0F1 ATP synthase subunit beta	ATP synthase beta chain (EC 3.6.3.14)	F0F1 ATP synthase subunit beta (EC 3.6.3.14)	
**254780614**	537021.9.peg.629	F0F1 ATP synthase subunit epsilon	ATP synthase epsilon chain (EC 3.6.3.14)	F0F1 ATP synthase subunit epsilon (EC 3.6.3.14)	
**254780613**	537021.9.peg.630	adenylosuccinate synthetase	Adenylosuccinate synthetase (EC 6.3.4.4)	adenylosuccinate synthetase (EC 6.3.4.4)	
**254780612**	537021.9.peg.631	putative transmembrane protein	hypothetical protein	unknown	transmembrane protein
**254780611**	537021.9.peg.632	RNA polymerase factor sigma-32	RNA polymerase sigma factor RpoH	RNA polymerase factor sigma-32	
**254780610**	537021.9.peg.633	RNA-pseudouridylate synthase protein, ribosomal large subunit D	Ribosomal large subunit pseudouridine synthase D (EC 4.2.1.70)	ribosomal large subunit pseudouridine synthase D (EC 4.2.1.70)	
**255764495**	537021.9.peg.635	putative phosphoesterase protein	putative phosphohydrolases, Icc family	phosphoesterase protein	
**254780608**	537021.9.peg.636	ribosomal large subunit pseudouridine synthase C	Ribosomal large subunit pseudouridine synthase C (EC 4.2.1.70) LSU Psi955, Psi2504 and Psi2580	ribosomal large subunit pseudouridine synthase C (EC 4.2.1.70)	
**254780607**	537021.9.peg.637	hypothetical protein	Putative secreted protein	unknown	has signal peptide
**254780606**	537021.9.peg.638	cell division protein	Cell division protein FtsK	cell division protein FtsK	
**254780605**	537021.9.peg.639	putative aminopeptidase	Xaa-Pro aminopeptidase (EC 3.4.11.9)	aminopeptidase	
**254780604**	537021.9.peg.640	5-aminolevulinate synthase	5-aminolevulinate synthase (EC 2.3.1.37)	5-aminolevulinate synthase (EC 2.3.1.37)	
**254780603**	537021.9.peg.641	tRNA/rRNA methyltransferase protein	rRNA methylases	rRNA methylases	
**254780602**	537021.9.peg.642	aspartate aminotransferase	Aspartate aminotransferase (EC 2.6.1.1)	aspartate aminotransferase	
**254780601**	537021.9.peg.643	ATP-dependent RNA helicase protein	ATP-dependent RNA helicase, DEAD/DEAH box	ATP-dependent RNA helicase protein	

			family		
**254780600**	537021.9.peg.644	CDP-diacylglycerol/glycerol-3-phosphate 3-phosphatidyltransferase	CDP-diacylglycerol--glycerol-3-phosphate 3-phosphatidyltransferase (EC 2.7.8.5)	CDP-diacylglycerol/glycerol-3-phosphate 3-phosphatidyltransferase (EC 2.7.8.5)	transmembrane protein
**255764496**	537021.9.peg.645	excinuclease ABC subunit C	Excinuclease ABC subunit C	excinuclease ABC subunit C, uvrC	
**254780598**	537021.9.peg.646	outer membrane protein	OmpA-like transmembrane region	outer membrane protein	has signal peptide
**255764497**	537021.9.peg.647	ABC transporter permease	ABC-type anion transport system, duplicated permease component	ABC-type importer for sulfonate-like oxoacid ion, transmembrane component	transmembrane protein
**254780596**	537021.9.peg.648	ABC transporter nucleotide binding/ATPase protein	ABC-type nitrate/sulfonate/bicarbonate transport system, ATPase component	ABC-type importer for sulfonate-like oxoacid ion, ATPase component	
**254780595**	537021.9.peg.649	nucleoside-diphosphate-sugar epimerase protein	Nucleoside-diphosphate-sugar epimerases	nucleoside-diphosphate-sugar epimerase protein	
**254780594**	537021.9.peg.650	Glutathione S-transferase domain protein	Glutathione S-transferase family protein	Glutathione S-transferase domain protein	
**254780593**	537021.9.peg.651	undecaprenyl pyrophosphate phosphatase	Undecaprenyl-diphosphatase (EC 3.6.1.27)	undecaprenyl pyrophosphate phosphatase (EC 3.6.1.27)	transmembrane protein
**254780592**	537021.9.peg.652	TPR repeat-containing protein	Flp pilus assembly protein TadD, contains TPR repeat	Flp pilus assembly protein, TadD	has signal peptide
**254780591**	537021.9.peg.653	aminomethyltransferase protein (glycine cleavage)	Folate-dependent protein for Fe/S cluster synthesis/repair in oxidative stress	aminomethyltransferase protein	
**254780590**	537021.9.peg.654	hypothetical protein	hypothetical protein	unknown	
**254780589**	537021.9.peg.655	hypothetical protein	probable exported protein STY0357	unknown	has signal peptide
**254780588**	537021.9.peg.656	acetyl-CoA carboxylase carboxyltransferase subunit alpha	Acetyl-coenzyme A carboxyl transferase alpha chain (EC 6.4.1.2)	acetyl-CoA carboxylase carboxyltransferase (EC 6.4.1.2)	
**255764498**	537021.9.peg.657	site-specific tyrosine recombinase XerD	Tyrosine recombinase XerD	site-specific tyrosine recombinase, XerD	
**254780586**	537021.9.peg.659	BolA family protein	Cell division protein BolA	Stress-induced morphogen, BolA family protein, involve in signal transduction	
**254780585**	537021.9.peg.660	molecular chaperone DnaJ family protein	FIG003437: hypothetical with DnaJ-like domain	molecular chaperone DnaJ family protein	
**254780584**	537021.9.peg.661	50S ribosomal protein L28	hypothetical protein	50S ribosomal protein L28	
**254780583**	537021.9.peg.662	glyoxalase II	Hydroxyacylglutathione hydrolase (EC 3.1.2.6)	glyoxalase II	
**254780582**	537021.9.peg.663	Methyltransferase type 11	SAM-dependent methyltransferase 2, in cluster with Hydroxyacylglutathione hydrolase (EC 3.1.2.6)	Methyltransferase	
**255764499**	537021.9.peg.664	1-acyl-sn-glycerol-3-phosphate acyltransferase	1-acyl-sn-glycerol-3-phosphate acyltransferase	1-acyl-sn-glycerol-3-phosphate acyltransferase	transmembrane protein
**254780579**	537021.9.peg.665	hypothetical protein	hypothetical protein	unknown	transmembrane protein
**254780578**	537021.9.peg.666	phosphatidylserine synthase	CDP-diacylglycerol--serine O-phosphatidyltransferase (EC 2.7.8.8)	phosphatidylserine synthase (EC 2.7.8.8)	transmembrane protein
**254780577**	537021.9.peg.667	phosphatidylserine decarboxylase	Phosphatidylserine decarboxylase (EC 4.1.1.65)	phosphatidylserine decarboxylase (EC 4.1.1.65)	transmembrane protein
**254780576**	537021.9.peg.668	ABC transporter related protein	putative ATP-binding component of ABC transporter	ABC-typle ATPase and permease for heavy metal export	transmembrane protein
**254780575**	537021.9.peg.669	hypothetical protein	hypothetical protein	permeases, RarD	transmembrane protein
**255764500**	537021.9.peg.670	hypothetical protein	hypothetical protein	unknown	has signal peptide
**254780573**	537021.9.peg.672	cysteinyl-tRNA synthetase	Cysteinyl-tRNA synthetase (EC 6.1.1.16)	cysteinyl-tRNA synthetase (EC 6.1.1.16)	
**254780572**	537021.9.peg.673	hypothetical protein	Similar to TadZ/CpaE, associated with Flp pilus assembly	Flp pilus assembly protein, TadG	transmembrane protein
**254780571**	537021.9.peg.674	hypothetical protein	hypothetical protein	Flp pilus assembly protein, TadG	transmembrane protein
**254780570**	537021.9.peg.675	phosphoribosylglycinamide formyltransferase	Phosphoribosylglycinamide formyltransferase (EC 2.1.2.2)	phosphoribosylglycinamide formyltransferase (EC 2.1.2.2)	
**254780569**	537021.9.peg.676	phosphoribosylaminoimidazole synthetase	Phosphoribosylformylglycinamidine cyclo-ligase (EC 6.3.3.1)	phosphoribosylaminoimidazole synthetase (EC 6.3.3.1)	
**254780568**	537021.9.peg.677	hypothetical protein	hypothetical protein	unknown	transmembrane protein
**254780567**	537021.9.peg.678	hypothetical protein	Chromosomal replication initiator protein DnaA	ATPase involved in DNA replication	
**254780565**	537021.9.peg.679	hypothetical protein	hypothetical protein	unknown	transmembrane protein
**254780564**	537021.9.peg.680	hypothetical protein	hypothetical protein	ABC transporter, extracellular binding protein	1/2 of the protein
**254780563**	537021.9.peg.681	extracellular solute-binding protein	iron ABC transporter, periplasmic iron-binding protein	ABC transporter, extracellular binding protein	has signal peptide, 1/2 of the protein
**254780562**	537021.9.peg.682	hypothetical protein	hypothetical protein	unknown	
**254780561**	537021.9.peg.684	thiamine transporter substrate binding subunit	Thiamin ABC transporter, substrate-binding component	ABC-type importer for Thiamine (vitamin B1), substrate binding component	has signal peptide
**255764501**	537021.9.peg.685	thiamine transporter membrane protein	Thiamin ABC transporter, transmembrane component	ABC-type importer for Thiamine (vitamin B1),	transmembrane protein

				transmembrane component	
**254780559**	537021.9.peg.686	thiamine transporter ATP-binding subunit	Thiamin ABC transporter, ATPase component / Thiamine transport ATP-binding protein thiQ	ABC-type importer for Thiamine (vitamin B1), ATPase component	
**254780558**	537021.9.peg.687	hypothetical protein	Possible divergent polysaccharide deacetylase	Possible divergent polysaccharide deacetylase	transmembrane protein
**254780557**	537021.9.peg.688	dinucleoside polyphosphate hydrolase	FIG000735: Adenosine (5')-pentaphospho-(5'')-adenosine pyrophosphohydrolase (EC 3.6.1.-)	dinucleoside polyphosphate hydrolase	
**254780556**	537021.9.peg.689	hypothetical protein	hypothetical protein	unknown	has signal peptide
**254780555**	537021.9.peg.690	hypothetical protein	hypothetical protein	prophage antirepressor	
**254780554**	537021.9.peg.692	Holliday junction resolvase	Crossover junction endodeoxyribonuclease RuvC (EC 3.1.22.4)	Holliday junction resolvase, ruvC (EC 3.1.22.4)	
**254780553**	537021.9.peg.693	Holliday junction DNA helicase RuvA	Holliday junction DNA helicase RuvA	Holliday junction DNA helicase RuvA	
**254780552**	537021.9.peg.694	Holliday junction DNA helicase RuvB	Holliday junction DNA helicase RuvB	Holliday junction DNA helicase RuvB	
**254780551**	537021.9.peg.695	tolQ protein	MotA/TolQ/ExbB proton channel family protein	biopolymer transport protein TolQ	transmembrane protein
**255764502**	537021.9.peg.696	hypothetical protein	hypothetical protein	Biopolymer transport protein	transmembrane protein
**254780549**	537021.9.peg.697	signal peptide protein	hypothetical protein	biopolymer transport protein TolA	transmembrane protein
**254780548**	537021.9.peg.698	translocation protein TolB	tolB protein precursor, periplasmic protein involved in the tonb-independent uptake of group A colicins	translocation protein TolB	has signal peptide
**254780547**	537021.9.peg.699	OmpA/MotB	18K peptidoglycan-associated outer membrane lipoprotein; Peptidoglycan-associated lipoprotein precursor; Outer membrane protein P6; OmpA/MotB precursor	Pal, the peptidoglycan-associated lipoprotein of Tol-Pal system	has signal peptide
**254780546**	537021.9.peg.700	hypothetical protein	tRNA(Ile)-lysidine synthetase	ATPase of the PP-loop superfamily involve in cell cycle control	
**254780545**	537021.9.peg.701	metalloprotease	Cell division protein FtsH (EC 3.4.24.-)	ATP-dependent Zn proteasese	transmembrane protein
**254780544**	537021.9.peg.702	phosphoglucosamine mutase protein	Phosphoglucosamine mutase (EC 5.4.2.10)	phosphoglucosamine mutase protein (EC 5.4.2.10)	
**254780543**	537021.9.peg.703	NADH dehydrogenase	NADH:ubiquinone oxidoreductase 17.2 kD subunit	NADH dehydrogenase	
**254780542**	537021.9.peg.704	hypothetical protein	hypothetical protein	unknown	has signal peptide
**255764503**	537021.9.peg.705	hypothetical protein	hypothetical protein	unknown	transmembrane protein
**254780540**	537021.9.peg.706	ABC transporter, membrane spanning protein (iron transport)	Manganese ABC transporter, inner membrane permease protein SitD	ABC-type importer for Manganese and iron, transmembrane component	transmembrane protein
**254780539**	537021.9.peg.707	ABC transporter, membrane spanning protein (iron)	Manganese ABC transporter, inner membrane permease protein SitC	ABC-type importer for Manganese and iron, transmembrane component	transmembrane protein
**254780538**	537021.9.peg.708	ABC transporter, nucleotide binding/ATPase protein (iron)	Manganese ABC transporter, ATP-binding protein SitB	ABC-type importer for Manganese and iron, ATPase component	
**254780537**	537021.9.peg.709	periplasmic solute binding protein	Manganese ABC transporter, periplasmic-binding protein SitA	ABC-type importer for Manganese and iron, substrate-binding component	has signal peptide
**254780536**	537021.9.peg.710	acyl-carrier-protein S-malonyltransferase	Malonyl CoA-acyl carrier protein transacylase (EC 2.3.1.39)	acyl-carrier-protein S-malonyltransferase (EC 2.3.1.39)	
**254780535**	537021.9.peg.711	3-ketoacyl-(acyl-carrier-protein) reductase	3-oxoacyl-[acyl-carrier protein] reductase (EC 1.1.1.100)	3-ketoacyl-(acyl-carrier-protein) reductase (EC 1.1.1.100)	
**254780534**	537021.9.peg.712	acyl carrier protein	Acyl carrier protein	acyl carrier protein	
**254780533**	537021.9.peg.713	3-oxoacyl-(acyl carrier protein) synthase II	3-oxoacyl-[acyl-carrier-protein] synthase, KASII (EC 2.3.1.41)	3-oxoacyl-(acyl carrier protein) synthase II (EC 2.3.1.41)	
**254780532**	537021.9.peg.714	aminodeoxychorismate lyase	FIG004453: protein YceG like	aminodeoxychorismate lyase, YceG-like	transmembrane protein
**254780531**	537021.9.peg.715	flagellin domain-containing protein	Flagellin protein FlaA	flagellin domain-containing protein	extracellular, flagellar component, exported by flagellar assembly machinery
**254780530**	537021.9.peg.716	hypothetical protein	hypothetical protein	flagellar assembly protein H	
**254780529**	537021.9.peg.717	flagellar motor protein MotB	Flagellar motor rotation protein MotB	flagellar motor protein MotB	transmembrane protein
**254780528**	537021.9.peg.718	chemotaxis protein	chemotaxis motility protein	chemotaxis protein	has signal peptide
**254780527**	537021.9.peg.719	hypothetical protein	hypothetical protein	unknown	
**254780526**	537021.9.peg.720	putative transcription regulator protein	Transcriptional regulator	transcriptional regulation protein	
**254780525**	537021.9.peg.721	flagellar hook protein FlgE	Flagellar hook protein FlgE	flagellar hook protein FlgE	extracellular, flagellar component, exported by flagellar assembly

					machinery
**254780524**	537021.9.peg.722	flagellar hook-associated protein FlgK	Flagellar hook-associated protein FlgK	flagellar hook-associated protein FlgK	extracellular, flagellar component, exported by flagellar assembly machinery
**254780523**	537021.9.peg.723	flagellar hook-associated protein FlgL	putative flagellar hook-associated protein	flagellar hook-associated protein FlgL	extracellular, flagellar component, exported by flagellar assembly machinery
**254780522**	537021.9.peg.724	flagellar biosynthesis regulatory protein FlaF	putative flagellar synthesis related protein	flagellar biosynthesis regulatory protein FlaF	
**254780521**	537021.9.peg.725	flagellar biosynthesis repressor FlbT	putative flagellum biosynthesis repressor protein	flagellar biosynthesis repressor FlbT	
**254780520**	537021.9.peg.726	flagellar basal body rod modification protein	Flagellar basal-body rod modification protein FlgD	flagellar basal body rod modification protein	extracellular, flagellar component, exported by flagellar assembly machinery
**254780519**	537021.9.peg.727	flagellar biosynthesis protein FliQ	Flagellar biosynthesis protein FliQ	flagellar biosynthesis protein FliQ	transmembrane protein
**255764504**	537021.9.peg.728	DNA-methyltransferase MKpn2kI	Cytosine-specific methyltransferase NlaX (EC 2.1.1.37) (M.NlaX)	type II DNA modification methyltransferase	1/2 of the protein
**254780516**	537021.9.peg.729	type II modification methyltransferase	Cytosine-specific methyltransferase NlaX (EC 2.1.1.37) (M.NlaX)	type II DNA modification methyltransferase	1/2 of the protein
**254780515**	537021.9.peg.730	hypothetical protein	hypothetical protein	restriction endonuclease	1/2 of the protein
**254780514**	N/A	type II restriction endonuclease	N/A	restriction endonuclease	1/2 of the protein
**254780512**	537021.9.peg.731	flagellar biosynthesis protein FlhA	Flagellar biosynthesis protein FlhA	flagellar biosynthesis protein FlhA	transmembrane protein
**254780511**	537021.9.peg.732	flagellar biosynthesis protein FliR	Flagellar biosynthesis protein FliR	flagellar biosynthesis protein FliR	transmembrane protein
**254780510**	537021.9.peg.733	hypothetical protein	hypothetical protein	unknown	
**254780509**	537021.9.peg.734	hypothetical protein	hypothetical protein	flgJ, peptidoglycan hydrolase	
**254780508**	537021.9.peg.735	hypothetical protein	hypothetical protein	unknown	
**254780507**	537021.9.peg.736	hypothetical protein	hypothetical protein	unknown	transmembrane protein
**254780506**	537021.9.peg.737	methylenetetrahydrofolate dehydrogenase/cyclohydrolase protein	Methylenetetrahydrofolate dehydrogenase (NADP+) (EC 1.5.1.5) / Methenyltetrahydrofolate cyclohydrolase (EC 3.5.4.9)	Methylenetetrahydrofolate dehydrogenase (NADP+) (EC 1.5.1.5) / Methenyltetrahydrofolate cyclohydrolase (EC 3.5.4.9)	
**254780505**	537021.9.peg.738	6-phosphogluconolactonase	6-phosphogluconolactonase (EC 3.1.1.31), eukaryotic type	6-phosphogluconolactonase (EC 3.1.1.31)	
**254780504**	537021.9.peg.739	glucose-6-phosphate 1-dehydrogenase	Glucose-6-phosphate 1-dehydrogenase (EC 1.1.1.49)	glucose-6-phosphate 1-dehydrogenase (EC 1.1.1.49)	
**254780503**	537021.9.peg.740	putative glutamine synthetase	glutamine synthetase family protein	putative glutamine synthetase	
**254780502**	537021.9.peg.741	ribonucleotide-diphosphate reductase subunit alpha	Ribonucleotide reductase of class Ia (aerobic), alpha subunit (EC 1.17.4.1)	ribonucleotide-diphosphate reductase subunit alpha (EC 1.17.4.1)	
**254780500**	537021.9.peg.742	hypothetical protein	hypothetical protein	Small protein A (tmRNA-binding)	transmembrane protein
**254780499**	537021.9.peg.743	hypothetical protein	Functional role page for Conserved hypothetical protein, gene in Ubiquinol-cytochrome C chaperone locus	unknown	
**254780498**	537021.9.peg.744	putative glycerol-3-phosphate acyltransferase PlsX	Phosphate:acyl-ACP acyltransferase PlsX	glycerol-3-phosphate acyltransferase	
**254780497**	537021.9.peg.745	3-oxoacyl-(acyl carrier protein) synthase III	3-oxoacyl-[acyl-carrier-protein] synthase, KASIII (EC 2.3.1.41)	3-oxoacyl-(acyl carrier protein) synthase III (EC 2.3.1.41)	
**254780496**	537021.9.peg.746	integration host factor subunit alpha	Integration host factor alpha subunit	integration host factor subunit alpha	
**254780495**	537021.9.peg.747	superoxide dismutase	Superoxide dismutase [Fe] (EC 1.15.1.1)	superoxide dismutase (EC 1.15.1.1)	
**254780494**	537021.9.peg.748	cation diffusion facilitator family transporter, putative	Cobalt-zinc-cadmium resistance protein	Co/Zn/Cd cation transporters	transmembrane protein
**254780493**	537021.9.peg.749	phosphoribosylaminoimidazole-succinocarboxamide synthase	Phosphoribosylaminoimidazole-succinocarboxamide synthase (EC 6.3.2.6)	phosphoribosylaminoimidazole-succinocarboxamide synthase (EC 6.3.2.6)	
**254780830**	N/A	hypothetical protein	N/A	unknown	
**254780831**	537021.9.peg.750	hypothetical protein	Scaffold protein for [4Fe-4S] cluster assembly ApbC, MRP-like	ATPase	
**254780832**	537021.9.peg.751	putative potassium uptake transport system protein	Kup system potassium uptake protein	K+ transporter	transmembrane protein
**254780833**	537021.9.peg.752	hypothetical protein	von Willebrand factor type A	von Willebrand factor type A fused with TadE/F involved in pilus assembly	transmembrane protein, but might be processed

					by cpaA and become periplasmic
**N/A**	537021.9.peg.753	N/A	DNA polymerase III alpha subunit (EC 2.7.7.7)	DNA polymerase III subunit alpha	1/2 of the protein
**N/A**	537021.9.peg.754	N/A	hypothetical protein	DNA polymerase III subunit alpha	1/2 of the protein
**254780834**	537021.9.peg.755	DNA primase	DNA primase (EC 2.7.7.-)	DNA primase (EC 2.7.7.-)	
**255764505**	537021.9.peg.756	polysialic acid capsule expression protein	Arabinose 5-phosphate isomerase (EC 5.3.1.13)	sugar phosphate isomerase involved in capsule formation	
**254780836**	537021.9.peg.757	putative type I restriction-modification system DNA methylase	Type I restriction-modification system, DNA-methyltransferase subunit M (EC 2.1.1.72)	Type I restriction-modification system, adenine methylase	
**254780837**	537021.9.peg.758	putative restriction endonuclease S subunit	Type I restriction-modification system, specificity subunit S (EC 3.1.21.3)	EcokI restriction-modification system protein, HsdS	
**254780840**	537021.9.peg.760	hypothetical protein	hypothetical protein	unknown	
**254780841**	537021.9.peg.761	phosphatidylcholine synthase protein	Phosphatidylcholine synthase (EC 2.7.8.24)	phosphatidylcholine synthase protein (EC 2.7.8.24)	transmembrane protein
**254780842**	537021.9.peg.762	2-octaprenyl-6-methoxyphenyl hydroxylase	hypothetical protein	2-octaprenyl-6-methoxyphenyl hydroxylase (EC 1.14.13.-)	
**254780843**	537021.9.peg.763	glucose/galactose transporter	glucose/galactose transporter	glucose/galactose/fucose transporter	transmembrane protein
**254780844**	537021.9.peg.764	hypothetical protein	hypothetical protein	unknown	has signal peptide
**254780845**	537021.9.peg.765	ribonucleotide-diphosphate reductase subunit beta	Ribonucleotide reductase of class Ia (aerobic), beta subunit (EC 1.17.4.1)	ribonucleotide-diphosphate reductase subunit beta (EC 1.17.4.1)	
**254780846**	537021.9.peg.766	bifunctional riboflavin kinase/FMN adenylyltransferase	Riboflavin kinase (EC 2.7.1.26) / FMN adenylyltransferase (EC 2.7.7.2)	bifunctional riboflavin kinase/FMN adenylyltransferase	
**254780847**	537021.9.peg.767	hypothetical protein	HAD superfamily protein involved in N-acetyl-glucosamine catabolism	sugar phosphatase/HAD superfamily hydrolase	
**254780848**	537021.9.peg.768	co-chaperonin GroES	Heat shock protein 60 family co-chaperone GroES	co-chaperonin GroES	
**254780849**	537021.9.peg.769	chaperonin GroEL	Heat shock protein 60 family chaperone GroEL	chaperonin GroEL	
**254780852**	537021.9.peg.770	NADH dehydrogenase subunit A	NADH ubiquinone oxidoreductase chain A (EC 1.6.5.3)	NADH dehydrogenase subunit A (EC 1.6.5.3)	transmembrane protein
**254780853**	537021.9.peg.771	NADH dehydrogenase subunit B	NADH-ubiquinone oxidoreductase chain B (EC 1.6.5.3)	NADH dehydrogenase subunit B (EC 1.6.5.3)	
**254780854**	537021.9.peg.772	NADH dehydrogenase subunit C	NADH-ubiquinone oxidoreductase chain C (EC 1.6.5.3)	NADH dehydrogenase subunit C (EC 1.6.5.3)	
**254780855**	537021.9.peg.773	NADH dehydrogenase subunit D	NADH-ubiquinone oxidoreductase chain D (EC 1.6.5.3)	NADH dehydrogenase subunit D (EC 1.6.5.3)	
**254780856**	537021.9.peg.774	NADH-quinone oxidoreductase, E subunit	NADH-ubiquinone oxidoreductase chain E (EC 1.6.5.3)	NADH-quinone oxidoreductase, E subunit (EC 1.6.5.3)	
**254780858**	537021.9.peg.775	NADH dehydrogenase I subunit F	NADH-ubiquinone oxidoreductase chain F (EC 1.6.5.3)	NADH dehydrogenase I subunit F (EC 1.6.5.3)	
**254780859**	537021.9.peg.776	NADH dehydrogenase subunit G	NADH-ubiquinone oxidoreductase chain G (EC 1.6.5.3)	NADH dehydrogenase subunit G (EC 1.6.5.3)	
**254780860**	537021.9.peg.777	NADH dehydrogenase subunit H	NADH-ubiquinone oxidoreductase chain H (EC 1.6.5.3)	NADH dehydrogenase subunit H (EC 1.6.5.3)	transmembrane protein
**254780861**	537021.9.peg.778	NADH dehydrogenase subunit I	NADH-ubiquinone oxidoreductase chain I (EC 1.6.5.3)	NADH dehydrogenase subunit I (EC 1.6.5.3)	
**254780862**	537021.9.peg.779	NADH dehydrogenase subunit J	NADH-ubiquinone oxidoreductase chain J (EC 1.6.5.3)	NADH dehydrogenase subunit J (EC 1.6.5.3)	transmembrane protein
**254780863**	N/A	hypothetical protein	N/A	NADH-ubiquinone oxidoreductase, chain 4L	transmembrane protein, 1/2 of the protein
**254780864**	537021.9.peg.780	NADH-ubiquinone oxidoreductase, chain 4L	NADH-ubiquinone oxidoreductase chain K (EC 1.6.5.3)	NADH-ubiquinone oxidoreductase, chain 4L	transmembrane protein, 1/2 of the protein
**254780865**	537021.9.peg.781	NADH dehydrogenase subunit L	NADH-ubiquinone oxidoreductase chain L (EC 1.6.5.3)	NADH dehydrogenase subunit L (EC 1.6.5.3)	transmembrane protein
**254780866**	537021.9.peg.782	NADH dehydrogenase subunit M	NADH-ubiquinone oxidoreductase chain M (EC 1.6.5.3)	NADH dehydrogenase subunit M (EC 1.6.5.3)	transmembrane protein
**254780867**	537021.9.peg.783	NADH dehydrogenase subunit N	NADH-ubiquinone oxidoreductase chain N (EC 1.6.5.3)	NADH dehydrogenase subunit N (EC 1.6.5.3)	transmembrane protein
**254780868**	537021.9.peg.784	birA bifunctional protein	Biotin-protein ligase (EC 6.3.4.15)	birA bifunctional protein (EC 6.3.4.15)	
**254780869**	537021.9.peg.785	beta-lactamase domain-containing protein	Metallo-beta-lactamase family protein, RNA-specific	beta-lactamase domain-containing protein, hydrolase	
**254780870**	537021.9.peg.787	prolyl-tRNA synthetase	Prolyl-tRNA synthetase (EC 6.1.1.15) Bacterial type	prolyl-tRNA synthetase (EC 6.1.1.15)	
**N/A**	537021.9.peg.788	N/A	Lipoprotein releasing system transmembrane protein LolC	lipoprotein ABC transporter, permease protein	transmembrane protein, 1/2 of the protein
**N/A**	537021.9.peg.789	N/A	hypothetical protein	lipoprotein ABC transporter, permease proteing	transmembrane protein, 1/2 of the protein
**254780871**	537021.9.peg.790	lipoprotein-releasing system ATP-binding protein lolD	Lipoprotein releasing system ATP-binding protein LolD	ABC-type exporter of lipoprotein, ATPase component	
**254780872**	537021.9.peg.791	3-demethylubiquinone-9 3-methyltransferase	3-demethylubiquinone-9 3-methyltransferase (EC 2.1.1.64)	3-demethylubiquinone-9 3-methyltransferase (EC 2.1.1.64)	
**254780873**	537021.9.peg.792	aspartate kinase	Aspartokinase (EC 2.7.2.4)	aspartate kinase (EC 2.7.2.4)	
**254780874**	537021.9.peg.793	peptide chain release factor 1	Peptide chain release factor 1	Protein chain release factor A	

**254780875**	537021.9.peg.794	protoporphyrinogen oxidase (methyltransferase) protein	Methylase of polypeptide chain release factors	Methylase of polypeptide chain release factor, HemK	
**254780876**	537021.9.peg.795	hypothetical protein	Eukaryotic translation initiation factor 3 subunit 10	unknown	
**254780877**	537021.9.peg.796	ATP-dependent Clp protease, ATP-binding subunit protein	ClpB protein	ATP-dependent Clp protease, ATP-binding subunit	
**254780878**	537021.9.peg.797	hypothetical protein	hypothetical protein	unknown	transmembrane protein
**254780879**	537021.9.peg.798	hypothetical protein	Membrane proteins related to metalloendopeptidases	Membrane proteins related to metalloendopeptidase	transmembrane protein
**254780880**	N/A	hypothetical protein	N/A	unknown	
**254780881**	537021.9.peg.799	Mrp protein	Mrp protein	unknown	
**254780882**	537021.9.peg.800	site-specific tyrosine recombinase XerC	Integrase	site-specific tyrosine recombinase XerC	
**254780883**	537021.9.peg.801	hypothetical protein	YidD	unknown	
**255764506**	537021.9.peg.802	GTP cyclohydrolase I	GTP cyclohydrolase I (EC 3.5.4.16) type 1	GTP cyclohydrolase I (EC 3.5.4.16)	
**254780885**	537021.9.peg.803	hypothetical protein	hypothetical protein	pilus assembly protein, PliZ	
**254780886**	N/A	hypothetical protein	N/A	unknown	has signal peptide, short piece
**254780889**	537021.9.peg.805	inosine 5'-monophosphate dehydrogenase	Inosine-5’-monophosphate dehydrogenase (EC 1.1.1.205)	inosine 5'-monophosphate dehydrogenase (EC 1.1.1.205)	
**254780890**	537021.9.peg.806	hypothetical protein	hypothetical protein	unknown	
**254780891**	537021.9.peg.807	hypothetical protein	hypothetical protein	two component system sensory kinase	
**254780892**	537021.9.peg.808	probable two-component response regulator protein	miscellaneous; not classified regulator	two-component response regulator	
**254780893**	537021.9.peg.809	two component response regulator	putative two-component response regulator transcriptional regulatory protein	two component response regulator	
**254780894**	537021.9.peg.810	hypothetical protein	Flagellar protein fliJ	possible flagellar protein fliJ	
**254780895**	537021.9.peg.811	hypothetical protein	hypothetical protein	unknown	
**254780896**	537021.9.peg.812	ABC transporter membrane spanning protein (branched chain amino acid)	Branched-chain amino acid transport system permease protein LivM (TC 3.A.1.4.1)	ABC-type permease	transmembrane protein
**254780897**	537021.9.peg.814	trigger factor	Cell division trigger factor (EC 5.2.1.8)	triger factor	
**254780898**	537021.9.peg.815	hypothetical protein	hypothetical protein	tyrosine/serine phosphatase	has signal peptide
**N/A**	537021.9.peg.816	N/A	DNA repair protein RadC	DNA repair protein RadC	1/2 of the protein
**N/A**	537021.9.peg.817	N/A	DNA repair protein RadC	DNA repair protein RadC	1/2 of the protein
**254780899**	537021.9.peg.819	methionine aminopeptidase	Methionine aminopeptidase (EC 3.4.11.18)	methionine aminopeptidase (EC 3.4.11.18)	
**254780900**	537021.9.peg.820	homoserine dehydrogenase	Homoserine dehydrogenase (EC 1.1.1.3)	homoserine dehydrogenase (EC 1.1.1.3)	
**254780901**	537021.9.peg.821	single-stranded-DNA-specific exonuclease protein	Single-stranded-DNA-specific exonuclease RecJ (EC 3.1.-.-)	single-stranded-DNA-specific exonuclease (EC 3.1.-.-)	
**254780902**	537021.9.peg.822	isocitrate dehydrogenase	Isocitrate dehydrogenase [NADP] (EC 1.1.1.42)	isocitrate dehydrogenase (EC 1.1.1.42)	
**254780903**	537021.9.peg.823	two-component sensor histidine kinase protein	Two-component sensor histidine kinase PleC	two-component sensor histidine kinase protein	transmembrane protein
**254780904**	537021.9.peg.824	tRNA/rRNA methyltransferase	tRNA:Cm32/Um32 methyltransferase	tRNA/rRNA methyltransferase	
**254780905**	537021.9.peg.825	glutamate racemase	Glutamate racemase (EC 5.1.1.3)	glutamate racemase (EC 5.1.1.3)	
**254780906**	537021.9.peg.826	hypothetical protein	hypothetical protein	unknown	has signal peptide
**254780907**	N/A	hypothetical protein	N/A	unknown	has signal peptide
**254780908**	537021.9.peg.827	hypothetical protein	hypothetical protein	unknown	
**254780909**	537021.9.peg.828	hypothetical protein	hypothetical protein	unknown	has signal peptide
**255764507**	537021.9.peg.829	guanylate kinase	Guanylate kinase (EC 2.7.4.8)	guanylate kinase (EC 2.7.4.8)	
**254780911**	537021.9.peg.830	formyltetrahydrofolate deformylase	Formyltetrahydrofolate deformylase (EC 3.5.1.10)	formyltetrahydrofolate deformylase (EC 3.5.1.10)	
**254780912**	537021.9.peg.831	hypothetical protein	hypothetical protein	unknown	
**254780913**	537021.9.peg.832	cold shock protein	Cold shock protein CspE	cold shock protein	
**254780914**	537021.9.peg.833	hypothetical protein	hypothetical protein	unknown	has signal peptide
**254780915**	537021.9.peg.835	oligoendopeptidase F	Oligoendopeptidase F	oligoendopeptidase F	
**254780916**	537021.9.peg.836	putative sigma-54-dependent transcription regulator protein	two component, sigma54 specific, transcriptional regulator, Fis family	transcriptional regulator and a receiptor in two component system	
**254780917**	537021.9.peg.837	ABC transporter, nucleotide binding/ATPase protein	Y4gM	ABC-type exporter for multiple drug and lipid, transmembrane and ATPase component	transmembrane protein
**254780918**	537021.9.peg.838	glycosyl transferase family protein	Glycosyl transferase, group 2 family protein	COG0463 Glycosyltransferases involved in cell wall biogenesis	

**254780919**	537021.9.peg.839	dTDP-4-dehydrorhamnose 3,5-epimerase	dTDP-4-dehydrorhamnose 3,5-epimerase (EC 5.1.3.13)	dTDP-4-dehydrorhamnose 3,5-epimerase (EC 5.1.3.13)	
**254780920**	537021.9.peg.840	dTDP-glucose 4,6-dehydratase	dTDP-glucose 4,6-dehydratase (EC 4.2.1.46)	dTDP-glucose 4,6-dehydratase (EC 4.2.1.46)	
**254780921**	537021.9.peg.841	dTDP-4-dehydrorhamnose reductase	dTDP-4-dehydrorhamnose reductase (EC 1.1.1.133)	dTDP-4-dehydrorhamnose reductase (EC 1.1.1.133)	
**254780922**	537021.9.peg.842	glucose-1-phosphate thymidylyltransferase	Glucose-1-phosphate thymidylyltransferase (EC 2.7.7.24)	glucose-1-phosphate thymidylyltransferase (EC 2.7.7.24)	
**254780923**	537021.9.peg.843	hypothetical protein	hypothetical protein	Lipopolysaccharide biosynthesis protein in cell wall biogenesis	
**254780924**	537021.9.peg.844	potassium-efflux system protein	Binding-protein-dependent transport systems inner membrane component:ATP/GTP-binding site motif A (P-loop) :TrkA-N:Potassium e	potassium-efflux system protein	transmembrane protein
**254780925**	537021.9.peg.845	bifunctional preprotein translocase subunit SecD/SecF	Protein-export membrane protein SecD (TC 3.A.5.1.1) / Protein-export membrane protein SecF (TC 3.A.5.1.1)	Preprotein translocase subunit SecD/SecF	transmembrane protein
**254780926**	537021.9.peg.846	hypothetical protein	hypothetical protein	unknown	
**254780927**	537021.9.peg.847	UDP-N-acetylglucosamine 1-carboxyvinyltransferase	UDP-N-acetylglucosamine 1-carboxyvinyltransferase (EC 2.5.1.7)	UDP-N-acetylglucosamine 1-carboxyvinyltransferase (EC 2.5.1.7)	
**254780928**	537021.9.peg.848	histidyl-tRNA synthetase	Histidyl-tRNA synthetase (EC 6.1.1.21)	histidyl-tRNA synthetase (EC 6.1.1.21)	
**254780929**	537021.9.peg.849	hypothetical protein	hypothetical protein	unknown	has signal peptide
**254780930**	537021.9.peg.850	nodulation protein (outer membrane efflux protein)	RND efflux system, outer membrane lipoprotein CmeC	Type I secretion system outer membrane transport protein	has signal peptide
**254780932**	537021.9.peg.853	hypothetical protein	hypothetical protein	unknown	
**254780933**	537021.9.peg.854	valyl-tRNA synthetase	Valyl-tRNA synthetase (EC 6.1.1.9)	valyl-tRNA synthetase (EC 6.1.1.9)	
**254780934**	537021.9.peg.855	hypothetical protein	von Willebrand factor type A	von Willebrand factor type A fused with TadE/F involved in pilus assembly	transmembrane protein, but might be processed by cpaA and become periplasmic
**254780935**	537021.9.peg.856	rare lipoprotein A	Rare lipoprotein A precursor	rare lipoprotein A	has signal peptide
**254780936**	537021.9.peg.857	hypothetical protein	Mll7752 protein	unknown	
**254780937**	537021.9.peg.858	DNA-directed RNA polymerase subunit omega	DNA-directed RNA polymerase omega subunit (EC 2.7.7.6)	DNA-directed RNA polymerase subunit omega	
**254780938**	537021.9.peg.859	4'-phosphopantetheinyl transferase	Holo-[acyl-carrier protein] synthase (EC 2.7.8.7)	4'-phosphopantetheinyl transferase (EC 2.7.8.7)	
**254780939**	537021.9.peg.860	type I signal peptidase	Signal peptidase I (EC 3.4.21.89)	type I signal peptidase (EC 3.4.21.89)	transmembrane protein
**254780940**	537021.9.peg.861	ribonuclease III	Ribonuclease III (EC 3.1.26.3)	ribonuclease III (EC 3.1.26.3)	
**254780941**	537021.9.peg.862	GTP-binding protein Era	GTP-binding protein Era	GTP-binding protein Era	
**254780942**	537021.9.peg.863	UDP-N-acetylglucosamine pyrophosphorylase protein	N-acetylglucosamine-1-phosphate uridyltransferase (EC 2.7.7.23) / Glucosamine-1-phosphate N-acetyltransferase (EC 2.3.1.157)	UDP-N-acetylglucosamine pyrophosphorylase (EC 2.7.7.23)	
**254780943**	537021.9.peg.864	glucosamine--fructose-6-phosphate aminotransferase	Glucosamine--fructose-6-phosphate aminotransferase [isomerizing] (EC 2.6.1.16)	glucosamine--fructose-6-phosphate aminotransferase (EC 2.6.1.16)	
**254780944**	537021.9.peg.865	hypothetical protein	hypothetical protein	unknown	transmembrane protein
**254780945**	537021.9.peg.866	ATP-dependent DNA helicase RecG	ATP-dependent DNA helicase RecG (EC 3.6.1.-)	ATP-dependent DNA helicase RecG (EC 3.6.1.-)	
**254780946**	537021.9.peg.867	hypothetical protein	YgfY COG2938	unknown	
**254780947**	537021.9.peg.868	transcription-repair coupling factor	Transcription-repair coupling factor	transcription-repair coupling factor	
**254780948**	537021.9.peg.870	DNA repair protein RecO	DNA recombination and repair protein RecO	DNA repair protein RecO	
**254780949**	537021.9.peg.871	tyrosyl-tRNA synthetase	Tyrosyl-tRNA synthetase (EC 6.1.1.1) cluster 1	tyrosyl-tRNA synthetase (EC 6.1.1.1)	
**255764508**	537021.9.peg.872	hypothetical protein	hypothetical protein	similar to YdcF-like protein, function unknown	transmembrane protein
**254780951**	537021.9.peg.875	hypothetical protein	hypothetical protein	unknown	has signal peptide
**254780952**	537021.9.peg.876	DNA helicase II	ATP-dependent DNA helicase UvrD/PcrA	DNA helicase II	
**254780953**	537021.9.peg.877	OmpA/MotB	OmpA/MotB domain protein	outer membrane lipoprotein	has signal peptide
**254780954**	537021.9.peg.878	hypothetical protein	hypothetical protein	Uncharacterized protein involved in outer membrane biogenesis	has signal peptide
**254780955**	537021.9.peg.879	hypothetical protein	hypothetical protein	ClpXP protease specificity-enhancing factor, SspB	
**254780956**	537021.9.peg.881	thymidylate synthase	Thymidylate synthase (EC 2.1.1.45)	thymidylate synthase (EC 2.1.1.45)	
**254780957**	537021.9.peg.882	dihydrofolate reductase protein	Dihydrofolate reductase (EC 1.5.1.3)	dihydrofolate reductase protein (EC 1.5.1.3)	
**254780958**	537021.9.peg.883	HflK protein	HflK protein	Membrane protease subunit, hflK	transmembrane protein
**254780959**	537021.9.peg.884	putative hydrolase serine protease transmembrane	HflC protein	Membrane protease subunit, hflC	has signal peptide

		protein			
**254780960**	537021.9.peg.886	phosphoserine phosphatase SerB	Phosphoserine phosphatase (EC 3.1.3.3)	phosphoserine phosphatase SerB (EC 3.1.3.3)	
**N/A**	537021.9.peg.887	N/A	tRNA delta(2)-isopentenylpyrophosphate transferase (EC 2.5.1.8)	tRNA delta(2)-isopentenylpyrophosphate transferase	1/2 of the protein
**N/A**	537021.9.peg.888	N/A	tRNA delta(2)-isopentenylpyrophosphate transferase (EC 2.5.1.8)	tRNA delta(2)-isopentenylpyrophosphate transferase	1/2 of the protein
**254780961**	537021.9.peg.890	hypothetical protein	hypothetical protein	peptidase	transmembrane protein
**254780962**	537021.9.peg.891	hypothetical protein	GCN5-related N-acetyltransferase	part of GCN5-related N-acetyltransferase, only a piece	strange, only piece of the protein
**254780963**	537021.9.peg.892	hypothetical protein	hypothetical protein	unknown	has signal peptide
**254780964**	537021.9.peg.893	ribonucleotide-diphosphate reductase subunit beta	Ribonucleotide reductase of class Ia (aerobic), beta subunit (EC 1.17.4.1)	ribonucleotide-diphosphate reductase subunit beta	
**254780965**	537021.9.peg.894	hypothetical protein	hypothetical protein	unknown	has signal peptide
**254780966**	537021.9.peg.895	hypothetical protein	hypothetical protein	unknown	
**254780967**	537021.9.peg.896	probable multidrug resistance transporter protein	Drug resistance transporter, Bcr/CflA family	multidrug resistance transporter	transmembrane protein
**254780968**	537021.9.peg.897	hypothetical protein	Uncharacterized monothiol glutaredoxin ycf64-like	Glutaredoxin-related protein	
**254780969**	537021.9.peg.898	hypothetical protein	YrbA protein	transcriptional regulator, BolA	
**254780970**	537021.9.peg.899	phosphoribosylformylglycinamidine synthase II	Phosphoribosylformylglycinamidine synthase, synthetase subunit (EC 6.3.5.3)	phosphoribosylformylglycinamidine synthase II (EC 6.3.5.3)	
**254780971**	537021.9.peg.900	phosphoribosylformylglycinamidine synthase I	Phosphoribosylformylglycinamidine synthase, glutamine amidotransferase subunit (EC 6.3.5.3)	phosphoribosylformylglycinamidine synthase I (EC 6.3.5.3)	
**254780972**	537021.9.peg.901	phosphoribosylformylglycinamidine synthase, PurS protein	Phosphoribosylformylglycinamidine synthase, PurS subunit (EC 6.3.5.3)	phosphoribosylformylglycinamidine synthase, PurS protein (EC 6.3.5.3)	
**254780973**	537021.9.peg.902	phosphoribosylaminoimidazole-succinocarboxamide synthase	Phosphoribosylaminoimidazole-succinocarboxamide synthase (EC 6.3.2.6)	phosphoribosylaminoimidazole-succinocarboxamide synthase (EC 6.3.2.6)	
**254780974**	537021.9.peg.903	adenylosuccinate lyase	Adenylosuccinate lyase (EC 4.3.2.2)	adenylosuccinate lyase (EC 4.3.2.2)	
**254780975**	537021.9.peg.904	D-ribulose-5 phosphate 3-epimerase protein	Ribulose-phosphate 3-epimerase (EC 5.1.3.1)	D-ribulose-5 phosphate 3-epimerase protein (EC 5.1.3.1)	
**254780976**	537021.9.peg.906	Holliday junction resolvase YqgF	Putative Holliday junction resolvase (EC 3.1.-.-)	Holliday junction resolvase YqgF-like protein (EC 3.1.-.-)	
**254780977**	537021.9.peg.907	putative glutamyl-tRNA(Gln) amidotransferase subunit C protein	Aspartyl-tRNA(Asn) amidotransferase subunit C (EC 6.3.5.6), Glutamyl-tRNA(Gln) amidotransferase subunit C (EC 6.3.5.7)	aspartyl/glutamyl-tRNA amidotransferase subunit C (EC 6.3.5.6)	
**254780978**	537021.9.peg.908	aspartyl/glutamyl-tRNA amidotransferase subunit A	Aspartyl-tRNA(Asn) amidotransferase subunit A (EC 6.3.5.6), Glutamyl-tRNA(Gln) amidotransferase subunit A (EC 6.3.5.7)	aspartyl/glutamyl-tRNA amidotransferase subunit A (EC 6.3.5.6)	
**254780979**	537021.9.peg.909	aspartyl/glutamyl-tRNA amidotransferase subunit B	Aspartyl-tRNA(Asn) amidotransferase subunit B (EC 6.3.5.6), Glutamyl-tRNA(Gln) amidotransferase subunit B (EC 6.3.5.7)	aspartyl/glutamyl-tRNA amidotransferase subunit B (EC 6.3.5.6)	
**254780980**	537021.9.peg.910	hypothetical protein	hypothetical protein	unknown	
**254780982**	537021.9.peg.914	putative homoserine/homoserine lactoneefflux protein	putative homoserine/homoserine lactone efflux protein	efflux system, permease	transmembrane protein
**254780983**	537021.9.peg.915	hypothetical protein	hypothetical protein	unknown	
**254780984**	N/A	hypothetical protein	N/A	unknown	has signal peptide
**254780985**	537021.9.peg.917	hypothetical protein	hypothetical protein	unknown	
**254780986**	537021.9.peg.918	hypothetical protein	hypothetical protein	unknown	transmembrane protein
**254780987**	537021.9.peg.919	hypothetical protein	hypothetical protein	transcriptional regulator	
**254780988**	537021.9.peg.920	hypothetical protein	hypothetical protein	unknown	transmembrane protein
**254780989**	537021.9.peg.921	GCN5-related N-acetyltransferase	GCN5-related N-acetyltransferase	N-acetyltransferase	
**254780991**	537021.9.peg.922	excinuclease ABC subunit B	Excinuclease ABC subunit B	excinuclease ABC subunit B, uvrB	
**254780992**	537021.9.peg.923	acyl-CoA hydrolase	acyl-CoA hydrolase	acyl-CoA hydrolase	
**254780993**	537021.9.peg.924	hypothetical protein	hypothetical protein	permease	transmembrane protein
**254780994**	537021.9.peg.925	hypothetical protein	hypothetical protein	unknown	
**254780995**	537021.9.peg.926	Endonuclease/exonuclease/phosphatase	hypothetical protein	exonuclease	1/2 of the protein
**254780996**	537021.9.peg.927	hypothetical protein	hypothetical protein	exonuclease	1/2 of the protein

**254780998**	537021.9.peg.928	hypothetical protein	Sulfur acceptor protein SufE for iron-sulfur cluster assembly	SufE protein probably involved in Fe-S cluster assembly	
**254780999**	537021.9.peg.929	NAD synthetase	NAD synthetase (EC 6.3.1.5) / Glutamine amidotransferase chain of NAD synthetase	NAD synthetase (EC 6.3.1.5)	
**254781000**	537021.9.peg.932	tRNA (uracil-5-)-methyltransferase	tRNA (Uracil54-C5-)-methyltransferase (EC 2.1.1.35)	tRNA (uracil-5-)-methyltransferase	
**254781001**	537021.9.peg.933	hypothetical protein	hypothetical protein	unknown	
**254781002**	537021.9.peg.934	hypothetical protein	hypothetical protein	unknown	transmembrane protein
**254781003**	537021.9.peg.935	hypothetical protein	hypothetical protein	endonuclease	has signal peptide
**254781004**	537021.9.peg.936	threonyl-tRNA synthetase	Threonyl-tRNA synthetase (EC 6.1.1.3)	threonyl-tRNA synthetase (EC 6.1.1.3)	
**254781005**	537021.9.peg.938	hypothetical protein	hypothetical protein	unknown	has signal peptide
**254781006**	537021.9.peg.939	ribonucleotide-diphosphate reductase subunit beta	Ribonucleotide reductase of class Ia (aerobic), beta subunit (EC 1.17.4.1)	ribonucleotide-diphosphate reductase subunit beta	
**254781007**	537021.9.peg.940	hypothetical protein	hypothetical protein	unknown	has signal peptide
**254781008**	537021.9.peg.941	hypothetical protein	hypothetical protein	unknown	
**254781009**	537021.9.peg.943	hypothetical protein	Flagellar basal-body rod protein FlgF	unknown	
**254781010**	537021.9.peg.944	hypothetical protein	hypothetical protein	unknown	has signal peptide
**254781011**	537021.9.peg.946	orotate phosphoribosyltransferase	Orotate phosphoribosyltransferase (EC 2.4.2.10)	orotate phosphoribosyltransferase (EC 2.4.2.10)	
**254781012**	537021.9.peg.947	dihydroorotase	Dihydroorotase (EC 3.5.2.3)	dihydroorotase (EC 3.5.2.3)	
**254781013**	537021.9.peg.948	phage-associated protein	hypothetical protein	phage associated protein	transmembrane protein
**254781014**	537021.9.peg.949	hypothetical protein	hypothetical protein	unknown	has signal peptide
**254781015**	537021.9.peg.951	hypothetical protein	hypothetical protein	unknown	
**254781016**	537021.9.peg.952	diphosphomevalonate decarboxylase/isopentenyl-diphosphate delta-isomerase	Diphosphomevalonate decarboxylase (EC 4.1.1.33)	diphosphomevalonate decarboxylase/isopentenyl-diphosphate delta-isomerase (EC 4.1.1.33)	
**254781017**	537021.9.peg.953	GHMP kinase	Mevalonate kinase (EC 2.7.1.36)	GHMP kinase (EC 2.7.1.36)	
**254781018**	537021.9.peg.954	GHMP kinase	Mevalonate kinase (EC 2.7.1.36)	GHMP kinase (EC 2.7.1.36)	
**254781019**	537021.9.peg.955	hydroxymethylglutaryl-coenzyme A synthase	Hydroxymethylglutaryl-CoA synthase (EC 2.3.3.10)	hydroxymethylglutaryl-coenzyme A synthase (EC 2.3.3.10)	
**254781020**	537021.9.peg.956	isopentenyl pyrophosphate isomerase	Isopentenyl-diphosphate delta-isomerase, FMN-dependent (EC 5.3.3.2)	isopentenyl pyrophosphate isomerase (EC 5.3.3.2)	
**255764509**	537021.9.peg.957	hydroxymethylglutaryl-coenzyme A (HMG-CoA) reductase	Hydroxymethylglutaryl-CoA reductase (EC 1.1.1.34)	hydroxymethylglutaryl-coenzyme A (HMG-CoA) reductase (EC 1.1.1.34)	
**254781022**	537021.9.peg.958	hypothetical protein	COG1988: Predicted membrane-bound metal-dependent hydrolases	possible membrane-bounded hydrolase	transmembrane protein
**254781024**	537021.9.peg.959	electron transfer flavoprotein-ubiquinone oxidoreductase	Electron transfer flavoprotein-ubiquinone oxidoreductase (EC 1.5.5.1)	electron transfer flavoprotein-ubiquinone oxidoreductase (EC 1.5.5.1)	
**254781025**	537021.9.peg.960	deoxyuridine 5 27-triphosphate nucleotidohydrolase	Deoxyuridine 5'-triphosphate nucleotidohydrolase (EC 3.6.1.23)	deoxyuridine 5 27-triphosphate nucleotidohydrolase (EC 3.6.1.23)	
**254781026**	537021.9.peg.962	hypothetical protein	MG(2+) CHELATASE FAMILY PROTEIN / ComM-related protein	Mg chelatase-related protein	1/3 of a protein
**254781027**	537021.9.peg.963	hypothetical protein	MG(2+) CHELATASE FAMILY PROTEIN / ComM-related protein	Mg chelatase-related protein	1/3 of a protein
**254781028**	537021.9.peg.964	putative Mg2+ chelatase family protein	putative Mg2+ chelatase family protein	Mg chelatase-related protein	1/3 of a protein
**254781029**	537021.9.peg.965	chemotaxis sensory transducer	hypothetical protein	unknown	transmembrane protein, has disordered domain
**254781031**	537021.9.peg.966	exsB protein	Queuosine Biosynthesis QueC ATPase	exsB/queuosine biosynthesis protein	
**254781032**	537021.9.peg.967	hypothetical protein	Queuosine biosynthesis QueD, PTPS-I	6-pyruvoyl-tetrahydropterin synthase	
**254781033**	537021.9.peg.968	HemY domain-containing protein	Homolog of E. coli HemY protein	HemY domain-containing protein	transmembrane protein
**254781034**	537021.9.peg.969	hypothetical protein	hypothetical protein	unknown	transmembrane protein
**255764510**	537021.9.peg.970	uroporphyrinogen-III synthase	Uroporphyrinogen-III synthase (EC 4.2.1.75)	uroporphyrinogen-III synthase (EC 4.2.1.75)	
**254781036**	537021.9.peg.971	hypothetical protein	hypothetical protein	unknown	
**254781037**	537021.9.peg.972	porphobilinogen deaminase	Porphobilinogen deaminase (EC 2.5.1.61)	porphobilinogen deaminase (EC 2.5.1.61)	
**254781038**	537021.9.peg.973	putative DNA-binding/iron metalloprotein/AP endonuclease	YgjD/Kae1/Qri7 family, required for threonylcarbamoyladenosine (t(6)A) formation in tRNA	Metal-dependent proteases	
**254781039**	537021.9.peg.974	putative glycerol-3-phosphate dehydrogenase	Glycerol-3-phosphate dehydrogenase [NAD(P)+] (EC 1.1.1.94)	putative glycerol-3-phosphate dehydrogenase (EC 1.1.1.94)	

**254781040**	537021.9.peg.975	hypothetical protein	Protein of unknown function DUF55	unknown	
**254781041**	537021.9.peg.976	succinate dehydrogenase protein, cytochrome b subunit	Succinate dehydrogenase cytochrome b-556 subunit	succinate dehydrogenase protein, cytochrome b subunit	transmembrane protein
**254781042**	537021.9.peg.977	succinate dehydrogenase hydrophobic membrane anchor	Succinate dehydrogenase hydrophobic membrane anchor protein	succinate dehydrogenase hydrophobic membrane anchor	transmembrane protein
**254781043**	537021.9.peg.978	succinate dehydrogenase flavoprotein subunit	Succinate dehydrogenase flavoprotein subunit (EC 1.3.99.1)	succinate dehydrogenase flavoprotein subunit (EC 1.3.99.1)	
**254781044**	537021.9.peg.979	succinate dehydrogenase iron-sulfur subunit	Succinate dehydrogenase iron-sulfur protein (EC 1.3.99.1)	succinate dehydrogenase iron-sulfur subunit (EC 1.3.99.1)	
**254781045**	537021.9.peg.980	outer membrane lipoprotein omp19	hypothetical protein	unknown	has signal peptide
**254781046**	537021.9.peg.981	hypothetical protein	hypothetical protein	unknown	
**254781047**	537021.9.peg.982	AFG1-family ATPase	hypothetical protein	AFG1-family ATPase	
**254781048**	537021.9.peg.983	malate dehydrogenase	Malate dehydrogenase (EC 1.1.1.37)	malate dehydrogenase (EC 1.1.1.37)	
**254781049**	537021.9.peg.984	succinyl-CoA synthetase subunit beta	Succinyl-CoA ligase [ADP-forming] beta chain (EC 6.2.1.5)	succinyl-CoA synthetase subunit beta (EC 6.2.1.5)	
**254781050**	537021.9.peg.985	succinyl-CoA synthetase subunit alpha	Succinyl-CoA ligase [ADP-forming] alpha chain (EC 6.2.1.5)	succinyl-CoA synthetase subunit alpha (EC 6.2.1.5)	
**254781051**	537021.9.peg.986	alpha-ketoglutarate decarboxylase	2-oxoglutarate dehydrogenase E1 component (EC 1.2.4.2)	alpha-ketoglutarate decarboxylase (EC 1.2.4.2)	
**254781052**	537021.9.peg.987	dihydrolipoamide succinyltransferase	Dihydrolipoamide succinyltransferase component (E2) of 2-oxoglutarate dehydrogenase complex (EC 2.3.1.61)	dihydrolipoamide succinyltransferase (EC 2.3.1.61)	
**254781053**	537021.9.peg.988	dihydrolipoamide dehydrogenase	Dihydrolipoamide dehydrogenase of 2-oxoglutarate dehydrogenase (EC 1.8.1.4)	dihydrolipoamide dehydrogenase (EC 1.8.1.4)	
**254781054**	537021.9.peg.989	ribose-5-phosphate isomerase A	Ribose 5-phosphate isomerase A (EC 5.3.1.6)	ribose-5-phosphate isomerase A (EC 1.8.1.7)	
**254781055**	537021.9.peg.990	glutathione reductase	Glutathione reductase (EC 1.8.1.7)	glutathione reductase (EC 1.8.1.7)	
**254781056**	537021.9.peg.991	phage-related lysozyme	prophage LambdaMc01, lysozyme(EC:3.2.1.17)	phage-related lysozyme, degrade peptidoglycan	
**254781057**	N/A	hypothetical protein	N/A	phage-related lysozyme, degrade peptidoglycan	1/2 of the protein
**254781058**	537021.9.peg.992	phage-related lysozyme	prophage LambdaMc01, lysozyme	phage-related lysozyme, degrade peptidoglycan	1/2 of the protein
**254781059**	537021.9.peg.994	cysteine desulfurase activator complex subunit SufB	Iron-sulfur cluster assembly protein SufB	cysteine desulfurase activator complex subunit SufB	
**254781060**	537021.9.peg.995	ABC transporter, nucleotide binding/ATPase protein	Iron-sulfur cluster assembly ATPase protein SufC	SufC, ABC-type ATPase involve in Fe-S cluster assembly	
**254781061**	537021.9.peg.996	putative iron-sulfur cluster assembly protein	Iron-sulfur cluster assembly protein SufD	SufD, ABC-transporter involve in Fe-S cluster assembly	
**254781062**	537021.9.peg.997	putative aminotransferase involved in iron-sulfur cluster biogenesis	Cysteine desulfurase (EC 2.8.1.7), SufS subfamily	cystein Desulfurase, SufS (EC 2.8.1.7)	
**254781063**	537021.9.peg.998	hypothetical protein	PaaD-like protein (DUF59) involved in Fe-S cluster assembly	metal-sulfur cluster biosynthetic enzyme	
**254781064**	537021.9.peg.999	FeS assembly scaffold SufA	Iron binding protein SufA for iron-sulfur cluster assembly	FeS assembly scaffold SufA	
**254781065**	537021.9.peg.1000	L-lysine 2,3-aminomutase protein	Lysine 2,3-aminomutase (EC 5.4.3.2)	L-lysine 2,3-aminomutase protein (EC 5.4.3.2)	
**254781066**	537021.9.peg.1001	putative lysyl-tRNA synthetase protein	Lysyl-tRNA synthetase-related protein	lysyl-tRNA synthetase protein	
**254781067**	537021.9.peg.1002	elongation factor P	Translation elongation factor P	elongation factor P	
**254781068**	537021.9.peg.1003	hypothetical protein	hypothetical protein	glycoside hydrolase family protein	1/4 of the protein
**254781069**	537021.9.peg.1004	hypothetical protein	hypothetical protein	glycoside hydrolase family protein	1/4 of the protein
**254781070**	537021.9.peg.1005	hypothetical protein	hypothetical protein	glycoside hydrolase family protein	1/4 of the protein
**254781071**	537021.9.peg.1006	hypothetical protein	hypothetical protein	glycoside hydrolase family protein	1/4 of the protein
**254781072**	537021.9.peg.1007	hypothetical protein	hypothetical protein	unknown	
**254781073**	537021.9.peg.1008	coproporphyrinogen III oxidase	Coproporphyrinogen III oxidase, aerobic (EC 1.3.3.3)	coproporphyrinogen III oxidase (EC 1.3.3.3)	
**254781074**	537021.9.peg.1009	uracil-DNA glycosylase	Uracil-DNA glycosylase, family 1	uracil-DNA glycosylase	
**254781075**	537021.9.peg.1010	hypothetical protein	hypothetical protein	unknown	transmembrane protein
**254781076**	537021.9.peg.1011	ribonuclease HII	Ribonuclease HII (EC 3.1.26.4)	ribonuclease HII (EC 3.1.26.4)	
**254781077**	537021.9.peg.1012	hypothetical protein	hypothetical protein	unknown	
**254781078**	537021.9.peg.1013	hypothetical protein	hypothetical protein	outer membrane protein	has signal peptide
**254781079**	537021.9.peg.1014	diguanylate cyclase	diguanylate cyclase (GGDEF domain)	diguanylate cyclase	transmembrane protein

**254781080**	537021.9.peg.1015	hypothetical protein	COG3750: Uncharacterized protein conserved in bacteria	unknown	
**254781081**	537021.9.peg.1016	hypothetical protein	hypothetical protein	unknown	
**254781082**	537021.9.peg.1017	hypothetical protein	hypothetical protein	unknown	
**254781083**	537021.9.peg.1018	hypothetical protein	hypothetical protein	unknown	
**254781084**	537021.9.peg.1019	hypothetical protein	hypothetical protein	unknown	
**254781085**	537021.9.peg.1020	F0F1 ATP synthase subunit B	ATP synthase B chain (EC 3.6.3.14)	F0F1 ATP synthase subunit B (EC 3.6.3.14)	transmembrane protein
**254781086**	537021.9.peg.1021	F0F1 ATP synthase subunit B'	ATP synthase B' chain (EC 3.6.3.14)	F0F1 ATP synthase subunit B' (EC 3.6.3.14)	transmembrane protein
**254781087**	537021.9.peg.1022	H+transporting two-sector ATPase C subunit	ATP synthase C chain (EC 3.6.3.14)	H+transporting two-sector ATPase C subunit (EC 3.6.3.14)	transmembrane protein
**254781088**	537021.9.peg.1023	F0F1 ATP synthase subunit A	ATP synthase A chain (EC 3.6.3.14)	F0F1 ATP synthase subunit A (EC 3.6.3.14)	transmembrane protein
**254781089**	537021.9.peg.1024	hypothetical protein	hypothetical protein	unknown	
**254781090**	537021.9.peg.1025	hypothetical protein	hypothetical protein	unknown	
**254781091**	537021.9.peg.1026	putative pyridoxal-phosphate-dependent aminotransferase protein	Cysteine desulfurase (EC 2.8.1.7)	pyridoxal-phosphate-dependent aminotransferase protein (EC 2.8.1.7)	
**254781093**	537021.9.peg.1027	hypothetical protein	Alpha/beta hydrolase	hydrolase	
**254781094**	537021.9.peg.1028	glycyl-tRNA synthetase subunit beta	Glycyl-tRNA synthetase beta chain (EC 6.1.1.14)	glycyl-tRNA synthetase subunit beta (EC 6.1.1.14)	
**254781095**	537021.9.peg.1029	UDP-N-acetylenolpyruvoylglucosamine reductase	UDP-N-acetylenolpyruvoylglucosamine reductase (EC 1.1.1.158)	UDP-N-acetylenolpyruvoylglucosamine reductase (EC 1.1.1.158)	
**254781096**	537021.9.peg.1030	UDP-N-acetylmuramate--L-alanine ligase	UDP-N-acetylmuramate--alanine ligase (EC 6.3.2.8)	UDP-N-acetylmuramate--L-alanine ligase (EC 6.3.2.8)	
**254781097**	537021.9.peg.1031	N-acetylglucosaminyl transferase	UDP-N-acetylglucosamine--N-acetylmuramyl-(pentapeptide) pyrophosphoryl-undecaprenol N-acetylglucosamine transferase (EC 2.4.1.227)	N-acetylglucosaminyl transferase	
**254781098**	537021.9.peg.1032	cell division protein FtsW peptidoglycan synthesis	Cell division protein FtsW	cell division protein FtsW peptidoglycan synthetase	transmembrane protein
**254781099**	537021.9.peg.1033	UDP-N-acetylmuramoyl-L-alanyl-D-glutamate synthetase	UDP-N-acetylmuramoylalanine--D-glutamate ligase (EC 6.3.2.9)	UDP-N-acetylmuramoyl-L-alanyl-D-glutamate synthetase (EC 6.3.2.9)	
**254781100**	537021.9.peg.1034	phospho-N-acetylmuramoyl-pentapeptide-transferase	Phospho-N-acetylmuramoyl-pentapeptide-transferase (EC 2.7.8.13)	phospho-N-acetylmuramoyl-pentapeptide-transferase (EC 2.7.8.13)	transmembrane protein
**254781101**	537021.9.peg.1035	UDP-N-acetylmuramoylalanyl-D-glutamyl-2, 6-diaminopimelate/D-alanyl-D-alanyl ligase	UDP-N-acetylmuramoylalanyl-D-glutamyl-2,6-diaminopimelate--D-alanyl-D-alanine ligase (EC 6.3.2.10)	UDP-N-acetylmuramoylalanyl-D-glutamyl-2, 6-diaminopimelate/D-alanyl-D-alanyl ligase (EC 6.3.2.10)	
**254781102**	537021.9.peg.1036	UDP-N-acetylmuramoylalanyl-D-glutamate--2, 6-diaminopimelate ligase	UDP-N-acetylmuramoylalanyl-D-glutamate--2,6-diaminopimelate ligase (EC 6.3.2.13)	UDP-N-acetylmuramoylalanyl-D-glutamate--2,6-diaminopimelate ligase (EC 6.3.2.13)	
**254781103**	537021.9.peg.1037	penicillin-binding transmembrane protein	Cell division protein FtsI [Peptidoglycan synthetase] (EC 2.4.1.129)	penicillin-binding transmembrane protein (EC 2.4.1.129)	transmembrane protein
**254781104**	537021.9.peg.1038	hypothetical protein	hypothetical protein	unknown	transmembrane protein
**255764511**	537021.9.peg.1039	S-adenosyl-methyltransferase MraW	rRNA small subunit methyltransferase H	S-adenosyl-methyltransferase MraW	
**254781106**	537021.9.peg.1040	cell division protein MraZ	Cell division protein MraZ	cell division protein MraZ	
**254781107**	537021.9.peg.1041	phosphoglucomutase	Phosphoglucomutase (EC 5.4.2.2)	phosphoglucomutase (EC 5.4.2.2)	
**254781108**	537021.9.peg.1042	von Willebrand factor type A	hypothetical protein	von Willebrand factor type A fused with TadE/F involved in pilus assembly	transmembrane protein, but might be processed by cpaA and become periplasmic
**254781109**	N/A	hypothetical protein	N/A	unknown	transmembrane protein
**254781110**	537021.9.peg.1043	von Willebrand factor type A	conserved hypothetical protein	von Willebrand factor type A fused with TadE/F involved in pilus assembly	transmembrane protein, but might be processed by cpaA and become periplasmic
**254781111**	537021.9.peg.1045	geranyltranstransferase protein	Octaprenyl-diphosphate synthase (EC 2.5.1.-) / Dimethylallyltransferase (EC 2.5.1.1) / Geranyltranstransferase (farnesyldiphosphate synthase) (EC 2.5.1.10) / Geranylgeranyl pyrophosphate synthetase (EC 2.5.1.29)	geranyltranstransferase protein (EC 2.5.1.1)	
**254781112**	537021.9.peg.1046	putative amino acid-binding periplasmic ABC transporter protein	PUTATIVE AMINO ACID-BINDING PERIPLASMIC ABC TRANSPORTER PROTEIN	ABC-type substrate binding protein	has signal peptide
**254781113**	537021.9.peg.1047	amino acid ABC transporter (permease)	amino acid ABC transporter, permease protein	ABC-type permease	transmembrane protein

**254781114**	537021.9.peg.1048	glycosyl transferase family protein	Beta-1,4-galactosyltransferase	glycosyl transferase family protein	
**254781115**	537021.9.peg.1049	poly(A) polymerase protein	tRNA nucleotidyltransferase (EC 2.7.7.21) (EC 2.7.7.25)	poly(A) polymerase protein (EC 2.7.7.21) (EC 2.7.7.25)	
**255764512**	537021.9.peg.1050	preprotein tranlocase protein	Preprotein translocase subunit YajC (TC 3.A.5.1.1)	preprotein tranlocase protein, yajC	transmembrane protein
**255764513**	537021.9.peg.1051	glutamyl-tRNA synthetase	Glutamyl-tRNA synthetase (EC 6.1.1.17), Glutamyl-tRNA(Gln) synthetase (EC 6.1.1.24) unambiguous	glutamyl-tRNA synthetase (EC 6.1.1.17)	
**254781118**	537021.9.peg.1052	lysyl-tRNA synthetase	Lysyl-tRNA synthetase (class II) (EC 6.1.1.6)	lysyl-tRNA synthetase (EC 6.1.1.6)	
**254781119**	537021.9.peg.1053	hypothetical protein	hypothetical protein	unknown	transmembrane protein
**254781120**	537021.9.peg.1054	radical SAM protein	Ribosomal RNA large subunit methyltransferase N (EC 2.1.1.-)	radical SAM protein	
**254781121**	537021.9.peg.1055	hypothetical protein	conserved hypothetical signal peptide protein	unknown	has signal peptide
**254781122**	537021.9.peg.1056	putative thiamine pyrophosphokinase	Thiamin pyrophosphokinase (EC 2.7.6.2)	thiamine pyrophosphokinase (EC 2.7.6.2)	
**254781123**	537021.9.peg.1057	putative ABC transporter, ATP-binding protein	ABC transporter, ATP-binding protein	ABC-type ATPase involved in transposon excision regulation, Uup	
**254781124**	N/A	hypothetical protein	N/A	unknown	
**254781126**	N/A	hypothetical protein	N/A	unknown	
**254781127**	537021.9.peg.1059	hypothetical protein	hypothetical protein	unknown	
**254781128**	537021.9.peg.1060	hypothetical protein	hypothetical protein	outer membrane protein	has signal peptide
**254781129**	537021.9.peg.1061	hypothetical protein	hypothetical protein	unknown	
**254781130**	537021.9.peg.1062	hypothetical protein	hypothetical protein	unknown	
**254781131**	537021.9.peg.1063	hypothetical protein	hypothetical protein	unknown	1/2 of the protein
**N/A**	537021.9.peg.1064	N/A	hypothetical protein	unknown	has signal peptide, 1/2 of the protein
**254781133**	537021.9.peg.1065	hypothetical protein	hypothetical protein	N-acetyltransferase	
**254781134**	537021.9.peg.1066	hypothetical protein	conserved hypothetical protein	unknown	1/3 of the protein
**254781135**	537021.9.peg.1067	hypothetical protein	hypothetical protein	unknown	1/3 of the protein
**254781136**	537021.9.peg.1068	hypothetical protein	hypothetical protein	unknown	1/3 of the protein
**254781137**	537021.9.peg.1069	hypothetical protein	conserved hypothetical protein	unknown	
**254781138**	537021.9.peg.1070	hypothetical protein	hypothetical protein	unknown	should be phage protein
**254781139**	537021.9.peg.1071	hypothetical protein	hypothetical protein	unknown	1/2 of a protein
**254781140**	537021.9.peg.1072	hypothetical protein	hypothetical protein	unknown	1/2 of a protein
**N/A**	537021.9.peg.1073	N/A	hypothetical protein	phage major head protein	1/2 of the protein
**N/A**	537021.9.peg.1074	N/A	hypothetical protein	phage major head protein	1/2 of the protein
**254781141**	537021.9.peg.1075	hypothetical protein	hypothetical protein	unknown	
**254781143**	N/A	hypothetical protein	N/A	unknown	
**254781144**	537021.9.peg.1076	hypothetical protein	putative portal protein	unknown	1/2 of the protein
**254781145**	537021.9.peg.1077	hypothetical protein	putative portal protein	phage portal protein	1/2 of the protein
**N/A**	537021.9.peg.1078	N/A	PROBABLE DNA PACKAGING PROTEIN GP2	DNA packaging protein Gp2	1/3 of the protein
**N/A**	537021.9.peg.1079	N/A	PROBABLE DNA PACKAGING PROTEIN GP2	DNA packaging protein Gp2	1/3 of the protein
**N/A**	537021.9.peg.1080	N/A	PROBABLE DNA PACKAGING PROTEIN GP2	DNA packaging protein Gp2	1/3 of the protein
**254781146**	537021.9.peg.1081	hypothetical protein	hypothetical protein	unknown	
**254781147**	537021.9.peg.1082	transcriptional regulator	transcriptional regulator	transcriptional regulator	
**254781148**	537021.9.peg.1084	hypothetical protein	hypothetical protein	replicative DNA helicase	1/2 of the protein, but still too short to form a functional protein, the structure is not complete
**254781149**	537021.9.peg.1085	replicative DNA helicase	DnaB helicase	replicative DNA helicase	1/2 of the protein, but still too short to form a functional protein, the structure is not complete
**N/A**	537021.9.peg.1087	N/A	hypothetical protein	just piece of phage protein	piece of phage protein
**254781150**	N/A	hypothetical protein	N/A	replicative DNA helicase (only a piece of the protein)	
**254781151**	537021.9.peg.1088	hypothetical protein	hypothetical protein	unknown	1/2 of a protein
**254781152**	537021.9.peg.1089	hypothetical protein	hypothetical protein	unknown	1/2 of a protein
**254781153**	N/A	hypothetical protein	N/A	ribonucleotide-diphosphate reductase subunit beta	just a piece of the

				(only a piece of that protein)	homologous protein, maybe not real
**254781154**	537021.9.peg.1091	hypothetical protein	hypothetical protein	unknown	
**254781155**	N/A	hypothetical protein	N/A	unknown	
**254781156**	537021.9.peg.1092	hypothetical protein	hypothetical protein	unknown	has signal peptide
**254781157**	537021.9.peg.1093	hypothetical protein	hypothetical protein	unknown	has signal peptide
**254781158**	537021.9.peg.1094	peptidyl prolyl cis-trans isomerase D signal peptide protein	hypothetical protein	Parvulin-like peptidyl-prolyl isomerase	transmembrane protein
**254781159**	537021.9.peg.1095	hypothetical protein	hypothetical protein	unknown	has signal peptide
**254781160**	537021.9.peg.1097	16S rRNA m3U1498 methyltransferase	Ribosomal RNA small subunit methyltransferase E (EC 2.1.1.-)	16S rRNA m3U1498 methyltransferase (EC 2.1.1.-)	
**254781161**	537021.9.peg.1098	glutamate--cysteine ligase	Glutamate--cysteine ligase (EC 6.3.2.2)	glutamate--cysteine ligase (EC 6.3.2.2)	
**254781162**	537021.9.peg.1099	GCN5-related N-acetyltransferase	Acetyltransferase (EC 2.3.1.-)	acetyltransferase (EC 2.3.1.-)	
**254781163**	537021.9.peg.1100	hypothetical protein	hypothetical protein	unknown	transmembrane protein
**254781164**	537021.9.peg.1102	hypothetical protein	hypothetical protein	unknown	
**254781165**	537021.9.peg.1103	pyridoxamine 5'-phosphate oxidase	Pyridoxamine 5'-phosphate oxidase (EC 1.4.3.5)	pyridoxamine 5'-phosphate oxidase (EC 1.4.3.5)	
**254781166**	537021.9.peg.1104	enoyl-(acyl carrier protein) reductase	Enoyl-[acyl-carrier-protein] reductase [NADH] (EC 1.3.1.9)	enoyl-(acyl carrier protein) reductase (EC 1.3.1.9)	
**254781167**	537021.9.peg.1105	tRNA-dihydrouridine synthase A	tRNA dihydrouridine synthase A (EC 1.-.-.-)	tRNA-dihydrouridine synthase A (EC 1.-.-.-)	
**254781169**	537021.9.peg.1106	PTS system ascorbate-specific transporter subunit IIC	Ascorbate-specific PTS system, EIIC component	PTS system, transporter	transmembrane protein
**254781170**	537021.9.peg.1108	deoxyribodipyrimidine photolyase	Deoxyribodipyrimidine photolyase (EC 4.1.99.3)	deoxyribodipyrimidine photolyase (EC 4.1.99.3)	
**254781171**	537021.9.peg.1109	hypothetical protein	Proton/glutamate symport protein, Sodium/glutamate symport protein	Na+/H+-dicarboxylate symporter	transmembrane protein
**254781172**	537021.9.peg.1110	NAD-dependent DNA ligase LigA	DNA ligase (EC 6.5.1.2)	NAD-dependent DNA ligase LigA (EC 6.5.1.2)	
**255764514**	537021.9.peg.1111	DNA repair protein RecN	DNA repair protein RecN	DNA repair protein RecN	
**254781174**	537021.9.peg.1112	outer membrane assembly lipoprotein YfiO	Competence lipoprotein comL precursor	outer membrane assembly lipoprotein YfiO, may involve in DNA uptake	has signal peptide
**254781175**	537021.9.peg.1113	UDP-3-O-[3-hydroxymyristoyl] N-acetylglucosamine deacetylase	UDP-3-O-[3-hydroxymyristoyl] N-acetylglucosamine deacetylase (EC 3.5.1.-)	UDP-3-O-[3-hydroxymyristoyl] N-acetylglucosamin deacetylase (EC 3.5.1.-)	
**254781176**	537021.9.peg.1114	cell division protein FtsZ	Cell division protein FtsZ (EC 3.4.24.-)	cell division protein FtsZ	
**254781177**	537021.9.peg.1115	cell division protein	Cell division protein FtsA	cell division protein	
**254781178**	537021.9.peg.1116	cell division protein	Cell division protein ftsQ	cell division protein	transmembrane protein
**254781179**	537021.9.peg.1117	D-alanine--D-alanine ligase	D-alanine--D-alanine ligase (EC 6.3.2.4)	D-alanine--D-alanine ligase (EC 6.3.2.4)	
**254781180**	537021.9.peg.1118	hypothetical protein	hypothetical protein	unknown	
**254781181**	537021.9.peg.1119	hypothetical protein	hypothetical protein	unknown	transmembrane protein
**254781182**	537021.9.peg.1121	hypothetical protein	hypothetical protein	type I restriction enzyme	1/2 of a protein
**254781183**	537021.9.peg.1122	hypothetical protein	hypothetical protein	unknown	1/2 of a protein
**254781184**	537021.9.peg.1124	hypothetical protein	hypothetical protein	unknown	transmembrane protein
**254781186**	537021.9.peg.1125	hypothetical protein	hypothetical protein	phage protein, terminase large subunit	1/2 of a protein
**254781187**	537021.9.peg.1126	putative phage terminase, large subunit	terminase B protein, putative	phage protein, terminase large subunit	1/2 of a protein
**254781188**	537021.9.peg.1127	hypothetical protein	hypothetical protein	phage protein, terminase small subunit	
**254781189**	537021.9.peg.1129	hypothetical protein	hypothetical protein	unknown	transmembrane protein
**254781191**	537021.9.peg.1131	hypothetical protein	Phage protein	unknown	
**254781190**	537021.9.peg.1130	hypothetical protein	DR0530-like primase	primase	
**254781192**	537021.9.peg.1132	hypothetical protein	conserved hypothetical protein	single-strand DNA binding protein	
**254781193**	537021.9.peg.1133	putative DNA polymerase from bacteriophage origin	DNA polymerase, phage-associated	DNA polymerase from bacteriophage	
**254781194**	537021.9.peg.1134	hypothetical protein	hypothetical protein	endonuclease	
**255764515**	537021.9.peg.1135	SNF2 related	DNA helicase, phage-associated	ATP-dependent helicase	
**254781196**	537021.9.peg.1136	DNA ligase, NAD-dependent	DNA ligase, NAD-dependent	NAD-dependent DNA ligase	it is shorter than normal ones, only has one domain
**254781197**	537021.9.peg.1137	guanylate kinase	Guanylate kinase (EC 2.7.4.8)	guanylate kinase	
**254781198**	537021.9.peg.1138	guanylate kinase	hypothetical protein	unknown	transmembrane protein

**254781199**	537021.9.peg.1139	hypothetical protein	hypothetical protein	unknown	transmembrane protein
**N/A**	537021.9.peg.1140	N/A	hypothetical protein	unknown	transmembrane protein
**255764516**	537021.9.peg.1141	hypothetical protein	hypothetical protein	unknown	
**N/A**	537021.9.peg.1142	N/A	hypothetical protein	unknown	
**255764517**	537021.9.peg.1143	hypothetical protein	hypothetical protein	unknown	
**254781202**	537021.9.peg.1144	hypothetical protein	hypothetical protein	structural protein of bacteriophage	
**254781203**	537021.9.peg.1145	hypothetical protein	hypothetical protein	unknown	
**254781204**	537021.9.peg.1146	hypothetical protein	hypothetical protein	minor structure protein	
**254781205**	537021.9.peg.1147	hypothetical protein	hypothetical protein	unknown	
**254781206**	537021.9.peg.1148	hypothetical protein	hypothetical protein	unknown	
**254781207**	537021.9.peg.1149	hypothetical protein	hypothetical protein	unknown	has signal peptide
**254781208**	537021.9.peg.1150	hypothetical protein	hypothetical protein	unknown	
**254781209**	537021.9.peg.1151	hypothetical protein	hypothetical protein	tail tubular protein A of bicteriophage	
**254781210**	537021.9.peg.1152	hypothetical protein	hypothetical protein	major capsid protein	
**254781211**	537021.9.peg.1153	hypothetical protein	hypothetical protein	unknown	
**254781212**	537021.9.peg.1154	hypothetical protein	hypothetical protein	unknown	
**254781213**	537021.9.peg.1155	head-to-tail joining protein, putative	hypothetical protein	bacteriophage head-to-tail joining protein	
**254781214**	537021.9.peg.1156	hypothetical protein	hypothetical protein	unknown	
**254781215**	537021.9.peg.1157	putative phage terminase, large subunit	terminase B protein, putative	phage terminase, large subunit	
**254781216**	537021.9.peg.1158	hypothetical protein	hypothetical protein	unknown	
**254781217**	537021.9.peg.1159	hypothetical protein	hypothetical protein	phage protein, related to the packing of DNA, recognize DNA	
**254781218**	537021.9.peg.1160	hypothetical protein	hypothetical protein	transcriptional regulator	
**254781219**	537021.9.peg.1161	hypothetical protein	hypothetical protein	unknown	
**254781220**	537021.9.peg.1162	hypothetical protein	hypothetical protein	unknown	
**254781221**	537021.9.peg.1163	hypothetical protein	hypothetical protein	unknown	has signal peptide
**254781222**	537021.9.peg.1164	hypothetical protein	hypothetical protein	acyltransferase, phage related	1/2 of the protein
**254781223**	537021.9.peg.1165	intrrupted gp229, phage associated protein	hypothetical protein	acyltransferase, phage related	1/2 of the protein
**254781224**	537021.9.peg.1166	hypothetical protein	hypothetical protein	unknown	transmembrane protein
**254781225**	537021.9.peg.1167	P4 family phage/plasmid primase	hypothetical protein	P4 family phage/plasmid primase	
**254781226**	537021.9.peg.1168	hypothetical protein	hypothetical protein	unknown	
**254781227**	537021.9.peg.1169	hypothetical protein	hypothetical protein	unknown	
**254781228**	537021.9.peg.1170	hypothetical protein	hypothetical protein	unknown	
