# Supplementary material for: MESSA: MEta-Server for protein Sequence Analysis
Source: BMC Biol. 2012 Oct 2;10:82. doi: 10.1186/1741-7007-10-82 (PMC3519821; doi:10.1186/1741-7007-10-82)
Supplement: Additional file 2 — Curation of proteins predicted to be secreted by the Sec pathway. This file contains proteins that are predicted to have signal peptides by computer programs. For each protein, the evidence to support or refute the prediction and the final judgment after manual curation is listed. [file 1741-7007-10-82-S2.PDF]

GI	JUDGMENT	N1	N2	EVIDENCE
**254780384**	no SP, extracellular	0	0	1. Type I Secretion System substrate; 2. export by type I secretion system
**254780523**	No SP, extracellular	0	1	1. flagellar component; 2. do not have SP export by flagellar assembly machinery
**254780685**	No SP, outermembrane	0	0	1. flagellar component; 2. do not have SP export by flagellar assembly machinery
**254780380**	No SP, periplasmic	0	0	1. flagellar component; 2. do not have SP export by flagellar assembly machinery
**254780381**	No SP, periplasmic	0	0	1. flagellar component; 2. do not have SP export by flagellar assembly machinery
**254780379**	No SP, periplasmic	0	0	1. flagellar component; 2. do not have SP export by flagellar assembly machinery
**254780378**	No SP, extracellular	0	1	1. flagellar component; 2. do not have SP export by flagellar assembly machinery
**254780520**	No SP, extracellular	0	0	1. flagellar component; 2. do not have SP export by flagellar assembly machinery
**254780524**	No SP, extracellular	0	0	1. flagellar component; 2. do not have SP export by flagellar assembly machinery
**254780531**	No SP, extracellular	0	0	1. flagellar component; 2. do not have SP export by flagellar assembly machinery
**254780525**	No SP, extracellular	0	0	1. flagellar component; 2. do not have SP export by flagellar assembly machinery
**254780733**	No SP, periplasmic or extracellular	6	0	1. flp, pilus assembly protein; 2. has TMH but can be processed by TadV to become periplasmic. Do not have SP
**254780732**	No SP, periplasmic or extracellular	6	0	1. flp, pilus assembly protein; 2. has TMH but can be processed by TadV to become periplasmic. Do not have SP
**254780730**	No SP, periplasmic or extracellular	5	1	1. flp, pilus assembly protein; 2. has TMH but can be processed by TadV to become periplasmic. Do not have SP
**254780736**	No SP, periplasmic or extracellular	6	0	1. flp, pilus assembly protein; 2. has TMH but can be processed by TadV to become periplasmic. Do not have SP
**254780734**	No SP, periplasmic or extracellular	6	0	1. flp, pilus assembly protein; 2. has TMH but can be processed by TadV to become periplasmic. Do not have SP
**254781110**	No SP, periplasmic or extracellular	6	0	1. TadE or Tad F, pilus assembly protein; 2. has TMH but can be processed by TadV to become periplasmic. Do not have SP
**254780934**	No SP, periplasmic or extracellular	6	0	1. TadE or Tad F, pilus assembly protein; 2. has TMH but can be processed by TadV to become periplasmic. Do not have SP
**254780833**	No SP, periplasmic or extracellular	6	1	1. TadE or Tad F, pilus assembly protein; 2. has TMH but can be processed by TadV to become periplasmic. Do not have SP
**254780388**	No SP, periplasmic or extracellular	6	0	1. TadE or Tad F, pilus assembly protein; 2. has TMH but can be processed by TadV to become periplasmic. Do not have SP
**254781108**	No SP, periplasmic or extracellular	5	2	1. TadE or Tad F, pilus assembly protein; 2. has TMH but can be processed by TadV to become periplasmic. Do not have SP
**255764485**	SP, periplasmic or extracellular	0	0	1. CpaD, pilus assembly; 2. orthologs proved by Phobius and one mode of SignalP; 3. start point is wrong, should remove "MMVEYMITILFGGVCFKGLANMRSL ISCLKTIFWKNFFLRTL", then Phobius and one mode of SignalP predict SP
**254780707**	SP, periplasmic or extracellular	3	1	1. Function; 2. ortholog proved by both modes of SignalP and Phobius; 3. COG suggest this protein is usually periplasmic
**254780396**	SP, periplasmic or extracellular	6	1	1. function; 2. ortholog proved by both modes of SignalP and Phobius; 3. pfam suggest this protein contains domain that is usually periplasmic
**254780698**	SP, periplasmic or extracellular	4	1	1. function; 2. pfam suggest this protein contains domain that is usually periplasmic; 3. start point wrong, should remove "MLQQKIQSSAKKTFLTYPYGSIYIQRNIQL", then 4 program predict SPs
**254780930**	SP, periplasmic or extracellular	3	1	1. function; 2. ortholog proved by both modes of SignalP and Phobius
**254780700**	SP, periplasmic or extracellular?	6	1	1. ortholog proved by both modes of SignalP and Phobius;<br>2. COG suggest this protein is usually periplasmic
**254780772**	SP, periplasmic or extracellular?	6	1	1. function; 2. start point is wrong, should remove "MHKSTEDFRRIKRLLEKYFPRSFQ", then phobius will also predict SP
**254781112**	SP, periplasmic or extracellular?	6	1	1. function; 2. ortholog can be verified by both modes of SignalP and Phobius
**254780764**	SP, periplasmic or extracellular?	6	1	1. function; 2. Ortholog; 3. start point is wrong, should remove "MFTHAEKILYS LDLRKY", then both phibus and signalP predict it as signal protien
**254781174**	SP, periplasmic or extracellular?	6	1	1. function; 2. ortholog
**254780561**	SP, periplasmic or extracellular	4	2	Function
**254780314**	SP, periplasmic or extracellular	0	2	1. function; 2. ortholog proved by two modes of SignalP and Phobius
**254780635**	SP, periplasmic or extracellular	0	2	ortholog proved by one mode of SignalP and Phobius
**254780350**	SP, periplasmic or extracellular	4	2	ortholog proved by one mode of SignalP and Phobius, at least periplasm
**254780953**	SP, periplasmic or extracellular	0	2	1. function; 2. ortholog proved by one mode of SignalP and Phobius
**254780898**	SP, periplasmic or extracellular	6	2	1. function; 2. ortholog proved by one mode of SignalP and MEMSAT
**254780395**	SP, periplasmic or extracellular	2	2	1. function; 2. ortholog proved by two modes of SignalP and Phobius
**254780798**	SP, periplasmic or extracellular	5	2	1. function; 2. ortholog confirmed by two modes of SignalP
**254780728**	SP, periplasmic or extracellular	5	2	Function
**254780592**	SP, periplasmic or extracellular	4	2	Function
**254780376**	SP, periplasmic or extracellular	2	2	1. function, although it is Flagellar assembly protein, but this one is probably transported by Sec into periplasm to set up the flagellar machinery, it is FlgI, and it is an exception; 2. ortholog, confirmed by two modes of signalP too
**254780477**	SP, periplasmic or extracellular	5	2	ortholog proved by one mode of SignalP and Phobius
**254780745**	SP, periplasmic or extracellular	3	2	1. function; 2. pfam suggest this protein contains domain that is usually periplasmic

**254781221**	SP, periplasmic or extracellular	0	2	ortholog predicted to have SP
**254781045**	SP, periplasmic or extracellular	0	2	1. function; 2. ortholog, confirmed by two modes of signalP and phobius too
**254780221**	SP, periplasmic or extracellular	0	3	
**254780528**	SP, periplasmic or extracellular	0	3	Function
**254780959**	SP, periplasmic or extracellular	5	3	
**254780954**	SP, periplasmic or extracellular	5	3	
**254781078**	SP, periplasmic or extracellular	4	3	
**254780556**	SP, periplasmic or extracellular	2	3	
**254780906**	SP, periplasmic or extracellular	0	3	
**254780737**	SP, periplasmic or extracellular	0	3	
**254780735**	SP, periplasmic or extracellular	4	3	a bit weird, as it is flp homolog
**254780281**	SP, periplasmic or extracellular	5	3	Ortholog
**254780342**	SP, periplasmic or extracellular	2	3	Function
**254780984**	SP, periplasmic or extracellular	0	3	
**254780935**	SP, periplasmic or extracellular	4	3	Function
**254780547**	SP, periplasmic or extracellular	2	3	Function
**254781014**	SP, periplasmic or extracellular	4	3	
**254781128**	SP, periplasmic or extracellular	3	3	
**254780759**	SP, periplasmic or extracellular	0	3	
**254781010**	SP, periplasmic or extracellular	0	3	
**254780377**	SP, periplasmic or extracellular	3	3	function, although it is Flagellar assembly protein, but this one is probably transported by Sec into periplasm, it is FlgA, and it is an exception
**254780375**	SP, periplasmic or extracellular	0	3	
**254780374**	SP, periplasmic or extracellular	2	3	function, although it is Flagellar assembly protein, but this one is probably transported by Sec into periplasm, it is FlgH, and it is an exception
**254780717**	SP, periplasmic or extracellular	4	3	function
**254780435**	SP, periplasmic or extracellular	3	3	function
**254780965**	SP, periplasmic or extracellular	5	3	
**254780747**	SP, periplasmic or extracellular	6	3	1. Function; 2. structure prediction, the TMH predicted in the middle of the protein is not true judging from the structure template
**254780207**	SP, periplasmic or extracellular	0	3	
**254780141**	SP, periplasmic or extracellular	3	3	function
**254780589**	SP, periplasmic or extracellular	2	3	
**254780563**	SP, periplasmic or extracellular	0	4	function
**254780951**	SP, periplasmic or extracellular	3	4	
**254780170**	SP, periplasmic or extracellular	4	4	function
**254780308**	SP, periplasmic or extracellular	4	4	function
**254780907**	SP, periplasmic or extracellular	0	4	
**254780909**	SP, periplasmic or extracellular	0	4	
**254781156**	SP, periplasmic or extracellular	0	4	
**254781157**	SP, periplasmic or extracellular	0	4	
**254780607**	SP, periplasmic or extracellular	0	4	
**254780199**	SP, periplasmic or extracellular	5	4	function
**254781207**	SP, periplasmic or extracellular	5	4	ortholog
**254780542**	SP, periplasmic or extracellular	0	4	
**254780548**	SP, periplasmic or extracellular	5	4	function
**254781121**	SP, periplasmic or extracellular	0	4	
**254780209**	SP, periplasmic or extracellular	3	4	
**254780727**	SP, periplasmic or extracellular	3	4	function
**254780598**	SP, periplasmic or extracellular	0	4	function
**254780844**	SP, periplasmic or extracellular	4	4	
**254780537**	SP, periplasmic or extracellular	2	4	function
**254781003**	SP, periplasmic or extracellular	3	4	
**254780443**	SP, periplasmic or extracellular	4	4	
**254780220**	SP, periplasmic or extracellular	0	2	
**255764500**	SP, periplasmic or extracellular	2	2	
**254781159**	SP, periplasmic or extracellular	0	2	
**254781005**	SP, periplasmic or extracellular	0	2	
**254780886**	SP, periplasmic or extracellular	3	2	
**254780963**	SP, periplasmic or extracellular	3	2	
**254780914**	SP, periplasmic or extracellular?	6	2	
**254780135**	SP, periplasmic or extracellular?	6	2	
**254780929**	SP, periplasmic or extracellular?	6	2	
**254781007**	SP, periplasmic or extracellular?	6	2	
**254780311**	cytoplasmic	0	1	1. funciton as cytoplasmic enzyme; 2. structure prediction
**254780313**	cytoplasmic	0	1	1. funciton, work with ribosome; 2. structure prediction

**254780315**	cytoplasmic	0	1	
**254781149**	cytoplasmic	0	1	Function
**254781058**	cytoplasmic	0	1	1. function, this is the C-terminal part of phage lysozyme; 2. structure prediction
**254781197**	cytoplasmic	0	1	Function
**254781071**	cytoplasmic	0	1	1. this is just the middle piece of a protein; 2. structure prediction
**254780392**	cytoplasmic	0	1	1. Function; 2. structure prediction
**254780482**	cytoplasmic	0	1	1. function; 2. structure prediction
**255764495**	cytoplasmic	0	1	ortholog cannot be verified by either mode of signalP or Phobius
**255764462**	cytoplasmic	0	1	function
**254780908**	cytoplasmic	0	1	
**254780918**	cytoplasmic	0	1	
**254780912**	cytoplasmic	0	1	
**254781026**	cytoplasmic	0	1	1. function; 2. this is the middle part of a big protein; 3. structure prediction
**254780980**	cytoplasmic	0	1	
**254780195**	cytoplasmic	2	1	1. function; 2. structure prediction
**254780942**	cytoplasmic	0	1	function
**254781205**	cytoplasmic	0	1	
**254781162**	cytoplasmic	0	1	function
**254780168**	cytoplasmic	0	1	1. function; 2. structure prediction;<br>3. ortholog proved by one mode of SignalP and Phobius
**254780164**	cytoplasmic	0	1	Function
**254780768**	cytoplasmic	0	1	1. Ortholog; 2. pfam
**254780640**	cytoplasmic	0	1	Function
**254780543**	cytoplasmic	0	1	Function
**254780227**	cytoplasmic	3	1	function as metabolic enzyme
**254780724**	cytoplasmic	0	1	Function
**254780470**	cytoplasmic	0	1	1. function; 2. structure prediction
**254780920**	cytoplasmic	2	1	Function
**peg_1087**	cytoplasmic	0	1	
**254780132**	cytoplasmic	0	1	Function
**254780156**	cytoplasmic	0	1	Function
**254781097**	cytoplasmic	5	1	1. structure prediction; 2. tiger say this step occur in cytoplasm
**254780154**	cytoplasmic	0	1	
**254781099**	cytoplasmic	0	1	1. structure prediction; 2. ortholog can only be verified by Phobius;<br>3. PRK says this step is cytoplasmic
**254780787**	cytoplasmic	0	1	function, translation initiation factor IF-2
**254781036**	cytoplasmic	0	1	1. function; 2. pfam
**254781031**	cytoplasmic	2	1	1. Function; 2. structure prediction
**254780439**	cytoplasmic	2	1	1. function; 2. pfam
**254780434**	cytoplasmic	0	1	1. function; 2. pfam
**peg_473**	cytoplasmic	0	1	function
**254780322**	cytoplasmic	0	1	1. function; 2. structure prediction
**254780742**	cytoplasmic	0	1	1. function; 2. pfam
**254780219**	cytoplasmic	0	1	
**254780261**	cytoplasmic	0	1	function, ribosome protein
**254780266**	cytoplasmic	0	1	function, ribosome protein
**254780361**	cytoplasmic	0	1	function, ribosome protein
**254780687**	cytoplasmic	0	1	Function, flagellar cytoplasmic component
**254780682**	cytoplasmic?	0	1	
**254780510**	cytoplasmic?	0	1	
**254781039**	cytoplasmic	3	2	function as metabolic enzyme
**peg_887**	cytoplasmic	0	2	function in tRNA processing
**254781015**	cytoplasmic	0	2	this does not look like a complete protein, ortholog is a Multi-TMHs protein, but this one lost most of the transmembrane part.
**peg_1064**	cytoplasmic	0	2	ortholog not predicted to have SP by both modes of SignalP and Phobius
**254781048**	cytoplasmic	3	2	fuction in TCA cycle
**254780319**	cytoplasmic	0	3	function, ribosome protein
**255764476**	transmembrane, SP not likely real	6	1	1. function; 2. multi-TMHs
**255764499**	transmembrane	6	1	1. Single-TMH, major part cytoplasmic<br>2. ortholog cannot be verified by either mode of signalP or phobius
**254780140**	Transmembrane, SP not likely true	6	1	1. single-TMH, major part periplasmic<br>2. ortholog can be proved by only one mode of signalp
**254780519**	transmembrane, SP not likely real	6	1	1. Function, flagellar transmembrane component; 2. multi-TMHs
**254780695**	transmembrane, SP not likely real	6	1	1. Function, flagellar transmembrane component; 2. double-TMH
**255764488**	transmembrane, SP not likely real	6	1	1. Function, flagellar transmembrane component; 2. double-TMH
**254780507**	transmembrane, SP not likely true	6	1	1. Function, flagellar transmembrane component;

				2. Single-TMH, major part cytoplasmic
**254780545**	transmembrane, but SP not likely real	6	1	Single-TMH, major part cytoplasmic
**254780752**	transmembrane, SP not likely real	6	1	1. single-TMH, major part periplasmic; 2. ortholog
**254780577**	transmembrane, SP not likely real	6	1	1. function; 2. single-TMH, major part periplasmic
**254780623**	transmembrane, SP not likely real	6	1	1. Single-TMH at C-terminal, major part periplasmic; 2. ortholog
**254780436**	transmembrane, SP not likely real	4	1	single-TMH, major part periplasmic
**254780961**	transmembrane, SP not likely real?	6	1	1. single-TMH, major part periplasmic; 2. ortholog do not have SP by neither mode of SignalP or Phobius; 3. start point is wrong, should add "MSFNVRNFVLWI" in front, and it has one SP, one TMH
**255764508**	transmembrane, SP might be right	6	1	double-TMH
**254780367**	transmembrane, SP not likely real	6	1	multi-TMHs
**254780866**	transmembrane, SP not likely real	6	1	multi-TMHs
**254780568**	transmembrane, SP not likely real	6	1	multi-TMHs
**255764466**	transmembrane, SP not likely real	6	1	multi-TMHs
**254780402**	transmembrane, SP not likely real	6	1	double-TMH
**254780228**	transmembrane, SP not likely real	6	1	1. double-TMHs; 2. start wrong, should add "MMLILFI", then it has double TMHs
**254780852**	transmembrane, SP not likely real	6	1	multi-TMHs
**254780917**	transmembrane, SP not likely real	6	1	1. function; 2. multi-TMHs
**254780708**	transmembrane, SP not likely real	6	1	multi-TMHs
**254780705**	transmembrane, SP not likely real	6	1	1. function; 2. multi-TMHs
**254781033**	transmembrane, SP not likely real	6	1	multi-TMHs
**254780450**	transmembrane, SP not likely real	3	1	double-TMH
**254780774**	transmembrane, SP not likely real	6	1	multi-TMHs
**254781079**	transmembrane, SP not likely real	5	1	multi-TMHs
**254780793**	transmembrane, SP not likely real	6	1	multi-TMHs
**254780171**	transmembrane, SP not likely real	6	1	1. function; 2. multi-TMHs
**254780483**	transmembrane, SP not likely real	6	1	1. function; 2. multi-TMHs
**255764497**	transmembrane, SP not likely real	6	1	multi-TMHs
**255764468**	transmembrane, SP not likely real	6	1	1. multi-TMHs; 2. start point is wrong, should add "MSRKVRKFDTVFPFTNYI RYSIISLPVVFLGVVCCLLAIILIIFKDSLDNSISFGLFG" in front
**254780860**	transmembrane, SP not likely real	6	1	multi-TMHs
**255764512**	transmembrane, SP not likely real	6	1	1. Single-TMH, major part cytoplasmic<br>2. ortholog cannot be verified by either mode of signalP or phobius
**254781169**	transmembrane, SP not likely real	6	1	multi-TMHs
**254780799**	transmembrane, SP not likely real	6	1	multi-TMHs
**254780425**	transmembrane, SP not likely real	6	1	Multi-TMHs
**254780494**	transmembrane, SP not likely real	6	1	1. function; 2. Multi-TMHs
**254780335**	transmembrane, SP not likely real	6	1	Multi-TMHs
**254780721**	transmembrane, SP not likely real	6	1	Multi-TMHs
**254780298**	transmembrane, SP not likely real	6	1	1. function; 2. Multi-TMHs
**254780291**	transmembrane, SP not likely real	6	1	1. Single-TMH, major part periplasmic<br>2. ortholog predicted to have SP by only one mode of SignalP
**254780593**	transmembrane, SP not likely real	6	1	Multi-TMHs
**254780576**	transmembrane, SP not likely real	6	1	1. function; 2. Multi-TMHs
**254780841**	transmembrane, SP not likely real	6	1	Multi-TMHs
**254780924**	transmembrane, SP not likely real	6	1	Multi-TMHs
**254780925**	transmembrane, SP not likely real	6	1	1. function; 2. Multi-TMHs
**254781178**	transmembrane, SP not likely real	6	1	1. Multi-TMHs; 2. Ortholog
**254780719**	transmembrane, SP not likely real	6	1	1. function; 2. Multi-TMHs
**254781002**	transmembrane, SP not likely real	6	1	Multi-TMHs
**254780323**	transmembrane, SP not likely real	5	1	1. function; 2. multi-TMHs
**254780967**	transmembrane, SP not likely real	6	1	Multi-TMHs
**254780815**	transmembrane, SP not likely real	5	1	1. Single-TMH, major part cytoplasmic; 2. Pfam;<br>3. ortholog predicted to have SP only by Phobius
**254780749**	transmembrane, SP not likely real	6	1	Multi-TMHs
**254781189**	transmembrane, SP not likely real	6	1	Multi-TMHs
**254781042**	transmembrane, SP not likely real	6	1	Multi-TMHs
**254780558**	transmembrane, SP not likely real	6	1	1. function; 2. Pfam; 3.single TMH at N-terminus and mainly periplasmic,
**254781158**	transmembrane, SP not likely real	6	1	1. single TMH at N-terminus and mainly periplasmic;<br>2.ortholog has SP by Phobius and one mode of SignalP
**254780421**	transmembrane, SP not likely real	6	1	single TMH at N-terminus and mainly periplasmic,
**254780386**	transmembrane, SP not likely real	5	1	single TMH at N-terminus and mainly periplasmic,
**254780572**	transmembrane, SP not likely real	6	1	1. Function, pilus transmembrane component; 2. single-TMH, major part periplasmic
**254780679**	transmembrane, SP not likely real	6	2	might be single transmembrane protein, and the C-terminal is cytoplasmic
**254780671**	transmembrane, SP not likely real	6	2	1. function; 2. might be single-TMH after cleaving SP, major part is cytoplasmic
**254780532**	transmembrane, SP not likely real	5	2	1. single-TMH, major part periplasmic;

				2. ortholog can be proved by only one mode of signalp
**peg_237**	transmembrane, SP not likely real	6	2	single-TMH in the middle
**peg_789**	transmembrane, SP not likely real	6	2	multi-TMHs
**254781098**	transmembrane, SP not likely real	6	2	Multi-TMHs
**254780896**	transmembrane, but SP is probably real	6	2	1. function; 2. multi-TMHs
**254780372**	transmembrane, but SP is probably real	6	3	1. function, flagellar transmembrane component; 2. Multi-TMHs
**254780729**	trasmembrane, but SP might be true	6	3	1. function; 2. Multi-TMHs
**254780196**	trasmembrane, but SP might be true	6	3	1. function; 2. after cleaveage of SP, single-TMH, major cytoplasmic
**254780468**	transmembrane, but SP is probably real	6	4	Multi-TMHs

N1: number of methods (out of 6) that predict the protein to have transmembrane helix (helices)
N2: number of methods (out of 4) that predict the protein to have a signal peptide
SP: signal peptide
TMH: transmembrane helix

Cell color:
Yellow: periplamic or extracellular proteins that are without signal peptides and that are secreted in Sec-independent pathways;
Light green: proteins with signal peptides and they are likely function in the periplasmic or extracellular space;
Pink: cytoplasmic proteins
Light blue: transmembrane proteins that are located in the inner membrane of this Gram-negative bacterium.
